# Supplementary material for: Cluefish: mining the dark matter of transcriptional data series with over-representation analysis enhanced by aggregated biological prior knowledge
Source: NAR Genom Bioinform. 2025 Jul 30;7(3):lqaf103. doi: 10.1093/nargab/lqaf103 (PMC12309373; doi:10.1093/nargab/lqaf103)
Supplement: lqaf103_Supplemental_Files [file lqaf103_supplemental_files.zip › supp-1.pdf]

SUPPLEMENTARY DATA

Cluefish: mining the dark matter of transcriptional data series with over-representation analysis enhanced by aggregated biological prior knowledge

AUTHORS

Ellis FRANKLIN[1,2,*], Elise BILLOIR[1], Philippe VEBER[2], Jérémie OHANESSIAN[1], Marie Laure DELIGNETTE-MULLER[2], Sophie Martine PRUD'HOMME[1,*]

[1] Université de Lorraine, CNRS, LIEC, F-57000 Metz, France

[2] Université Claude Bernard Lyon 1, LBBE, UMR 5558, CNRS, VAS, F-69622 Villeurbanne, France

* To whom correspondence should be addressed. Email: ellis.franklin@univ-lorraine.fr. Correspondence may also be addressed to Sophie Martine PRUD'HOMME. Email: sophie.prud-homme@univ-lorraine.fr.

Present Address: [Ellis FRANKLIN], Université de Lorraine, CNRS, LIEC, F-57000 Metz, France

Table of Contents

Text S1. Parameter choices for Cluefish applied to the external datasets 3

Figure S1. Principal Component Analysis (PCA) plots showing the data before (A) and after (B) removal of one control sample (replicate). 4

Figure S2. Plots of the raw dose-response data for the transcripts of three Cyp26 genes: (A) cyp26a1 (ENSDART00000041728.7), (B) cyp26b1 (ENSDART00000110347.3), and (C) cyp26c1 (ENSDART00000077809.5). Grey dashed vertical lines represent the experimental doses. 5

Figure S3. Dose-response fitted curve and computed BMD point for the rxraa gene transcript (ENSDART00000080481.6). Grey dashed vertical lines represent the experimental doses. 6

Text S1. Parameter choices for Cluefish applied to the external datasets

Rat liver dataset

For the rat liver (*Rattus norvegicus*) dataset, we downloaded the TF and CoTF gene lists (Step 1). We extracted gene names from the dataset's original custom identifiers, then converted these to their corresponding Ensembl gene IDs (*Step 3*). We maintained the default confidence score (**0.9**) in STRING for the protein-protein interaction network and the inflation parameter (**4**) for MCL clustering, similar to the zebrafish dataset, due to the high-quality annotation and interaction data available for this species (*Step 5*). The lower cluster size filter was kept at **4** to maintain consistency in cluster definition (*Step 6*). The main adjustments were made to the functional enrichment parameters in *Step 7* to account for the tissue-specific nature of this dataset. As by default, we tested against GO:BP, KEGG and WP pathways. As we were working with liver-specific expression data rather than whole-organism transcriptomics, we applied a more restrictive biological function size filter (lower and upper limits of **10** and **130** genes, respectively) to focus on liver-relevant processes and exclude overly broad or tissue-irrelevant functions. The enrichment gene count filter was increased to **4** to align with the higher lower-limit of biological function size and to avoid enrichments driven by only one or two genes. The friendliness parameter was set to **2** to prevent overly promiscuous genes (such as central metabolic regulators) from being incorporated into multiple clusters, which could dilute cluster specificity (*Step 9*).

Poplar root dataset

For this dataset concerning *Populus trichocarpa* (to which the studied *Populus canadensis* was mapped), several parameter adjustments were necessary due to its status as a less-referenced organism. No TF or CoTF gene lists were available, therefor we skipped the regulatory status annotation step (*Step 1*). We converted the initial Ensembl gene IDs to UniProtKB/TrEMBL IDs, required in this case for the protein-correspondance in the STRING database (*Step 3*). To compensate for the more limited data on protein interaction in this non-model organism, we reduced the STRING confidence score to **0.6**, allowing more predicted interactions to be included in the network. We also adjusted the MCL inflation parameter to **2** (versus the default 4) to create fewer but larger clusters, preventing excessive fragmentation of the network into many small clusters with low confidence (*Step 5*). The lower cluster size filter was maintained at **4** (*Step 6*). For functional enrichment analysis, we tested against GO:BP and KEGG only, as the WP database lacked sufficient coverage for this organism. We retained a broad range for the biological function size filter (**5**-**800** genes) to accommodate the whole-transcriptome background and the less comprehensive functional annotation of poplar genes (*Step 7*). The enrichment gene count filter was reduced to **2**, allowing enrichment to be detected even with fewer genes per function, which is particularly important for smaller clusters in this less-annotated species. As with the rat dataset, the friendliness parameter was set to **2** to maintain cluster specificity while fishing lonely (unassigned) genes (*Step 9*).

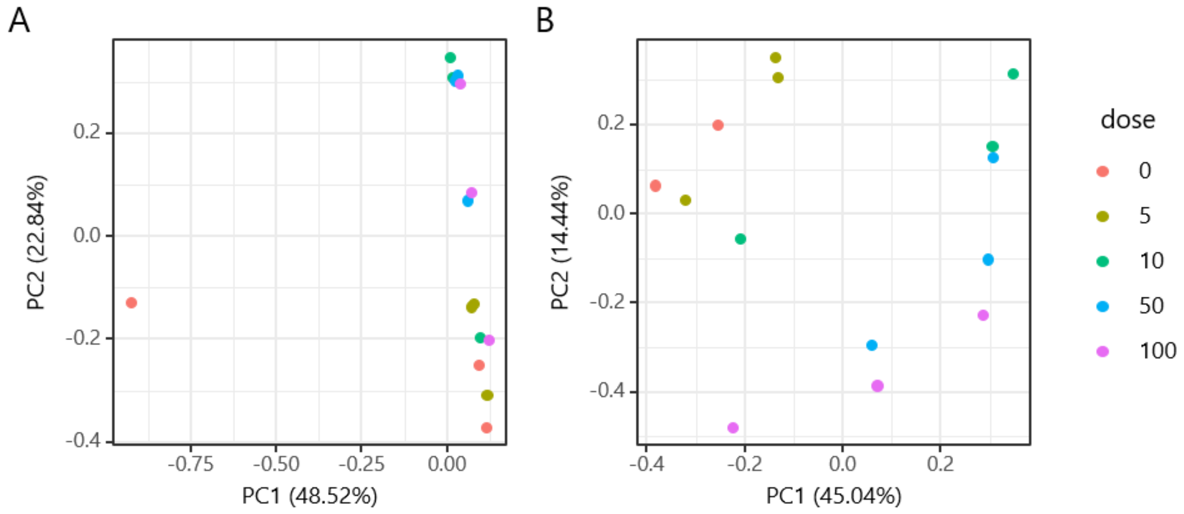

Figure S1. Principal Component Analysis (PCA) plots showing the data before (A) and after (B) removal of one control sample (replicate).

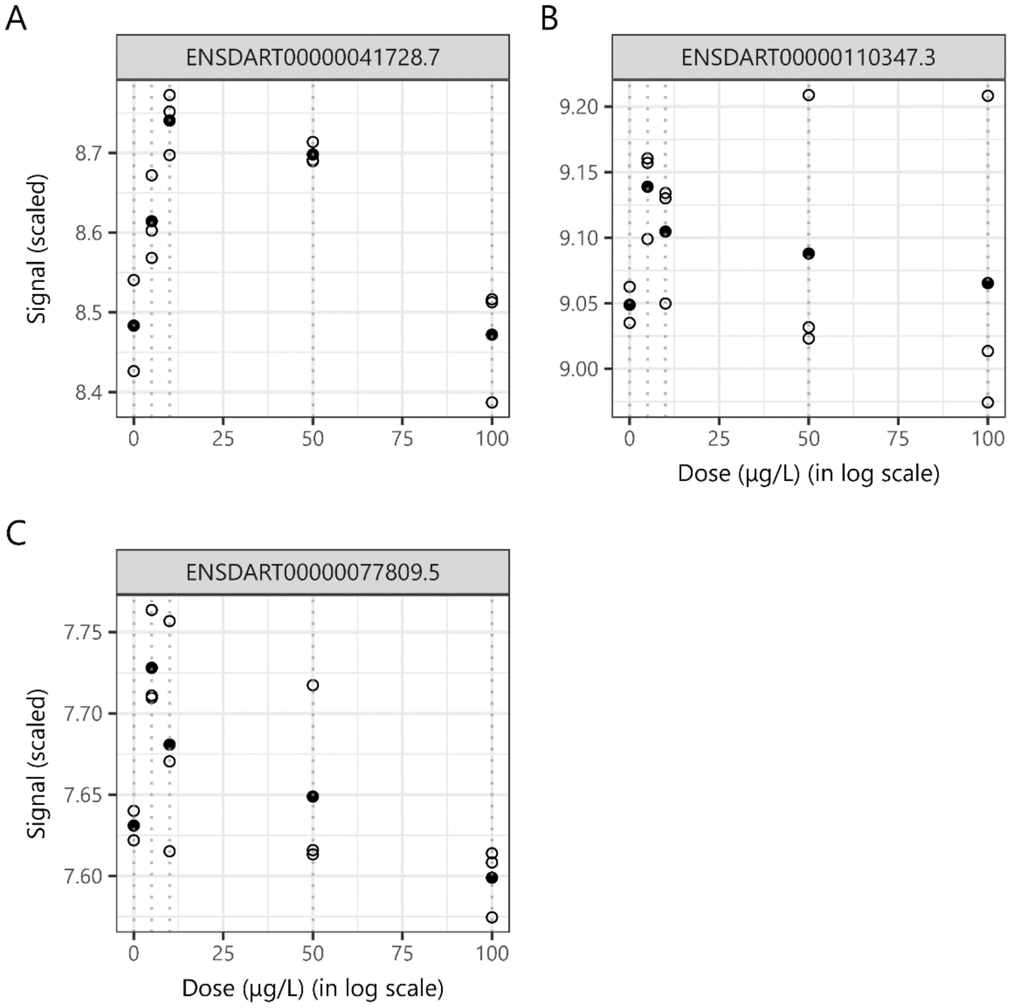

Figure S2. Plots of the raw dose-response data for the transcripts of three Cyp26 genes: (A) cyp26a1 (ENSDART00000041728.7), (B) cyp26b1 (ENSDART00000110347.3), and (C) cyp26c1 (ENSDART00000077809.5). Grey dashed vertical lines represent the experimental doses.

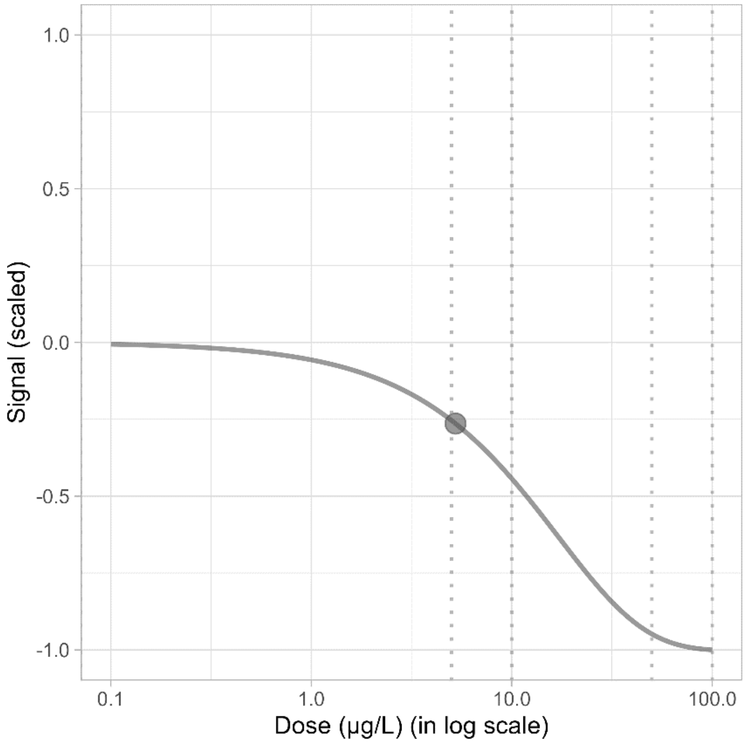

Figure S3. Dose-response fitted curve and computed BMD point for the rxraa gene transcript (ENSDART00000080481.6). Grey dashed vertical lines represent the experimental doses.
