# Supplementary material for: Cluefish: mining the dark matter of transcriptional data series with over-representation analysis enhanced by aggregated biological prior knowledge
Source: NAR Genom Bioinform. 2025 Jul 30;7(3):lqaf103. doi: 10.1093/nargab/lqaf103 (PMC12309373; doi:10.1093/nargab/lqaf103)
Supplement: lqaf103_Supplemental_Files [file lqaf103_supplemental_files.zip › supp-3.pdf]

Report of the workflow results

AUTHOR
Ellis Franklin

PUBLISHED
2024-08-19

1 Introduction

This document is a comprehensive report, meticulously encapsulating the pivotal results derived from the DRomics analysis while detailing the workflow. It succinctly summarizes the impacts and outputs of each significant step within the pipeline, supported by visual representations like plots depicting the summary of BMD values per cluster, aiding in prioritization. Moreover, additional specific visualizations for each cluster, such as plots of fitted curves, empirical cumulative distribution function (ECDF) plots of BMD values, and summary tables, enable a more focused exploration.

2 Results from the DRomics analysis

The total number of transcripts derived from this experiment : **39890**

Step	Number of transcripts concerned
Selection of deregulated transcripts	2449
Computation of BMD-1SD values	2449
Defined confidence interval around BMD	2433

Table 1: Summary table of DRomics analysis

3 Summary of the workflow

Identifier retrieval

Type of identifier	Background count	Deregulated count
Ensembl transcript	39890	2433
Ensembl gene	27720	2365

Table 2: Summary table of indentifier mapping

> **Note**
>
> The external_gene_name identifiers, which depict human-readable gene names, have been adjusted to address redundancy between Ensembl transcript or gene identifiers. As a result, the count of external_gene_name IDs matches the number of transcripts: 2433.

Cluster Gene Set Size Filtering

Clusters containing at least **4** Ensembl genes are kept for subsequent steps. Genes in the remaining clusters are designated as "lonely genes", signifying their absence from any cluster.

Step	Ensembl Gene Count	Cluster Count
Before clustrfiltr()	750	204
After clustrfiltr()	399	53

Table 3: Summary table of cluster and gene count before and after cluster filtering step

Cluster Functional Enrichment

Functional enrichment analysis is conducted individually for each cluster and data source. The parameters used in the `clustrenrich()` function to generate this output are as follows:

- background type (`bg_type`) = **custom_annotated**
- data sources (`sources`) = **GO:BP, KEGG, WP**
- p-value (`user_theshold`) = **0.05**
- multiple testing correction method (`correction_method`) = **fdr**
- minimum term size (`min_term_size`) = **5**
- maximum term size (`max_term_size`) = **500**
- choice of keeping only highlighted GO terms (`only_highlighted_GO`) = **TRUE**
- Number of genes required to keep enrichment (`ngenes_enrich_filtr`) = **3**

Metric	GO:BP	KEGG	Wikipathways
Background size	15468	5714	1383

Table 4: Summary of background gene lists involved in functional enrichment for each source

Metric	Query Ensembl Gene Count	Cluster Count	Total Term Count
Enrichment results before term filtering	316	52	218
Enrichment results after term filtering	286	47	97

Table 5: Summary table of gene, cluster and total term count during the cluster enrichment step

Metric	GO:BP	KEGG	Wikipathways
All terms associated with deregulated genes	3450	164	43

Metric	GO:BP	KEGG	Wikipathways
Enriched terms	114	91	18
Filtered enriched terms	41	51	10

Table 6: Summary table of term count per source and per characteristic

Cluster Fusion

Clusters merge when they share identical enriched terms.

Metric	Cluster count
Before fusion	53
After fusion	44

Table 7: Summary table of the cluster count before and after cluster fusion

old_clustr	after_GO_fusion	after_KEGG_fusion	after_WP_fusion
2	2	1	1
6	6	1	1
8	8	8	4
26	16	16	16
30	13	13	13
44	44	44	16
45	45	10	10
49	49	23	23
53	53	14	14

Table 8: Summary table of cluster fusion process

Lonely fishing

Lonely genes are incorporated into clusters if they share a common term with any given cluster. The friendly limit (friendly_limit) was set to **0** .

Metric	All	Annotated
Lonely genes before fishing	1966	1334
Lonely genes	505	505

Metric	All	Annotated
fished		
Lonely genes after fishing	1461	829

Table 9: Summary table of all and only biologically annotated genes involved in the lonely fishing

4 The results as an interactive table

Show 10 entries Search:

	transcript_id	gene_id	gene_name	NewCluster	Friendliness	Term_name	Source
	All	All	All	All	All	All	All
1	ENSDART00000000069.8	ENSDARG00000000068	slc9a3r1a_g2t1	Lonely	1		
2	ENSDART00000000070.7	ENSDARG00000000069	dap_g2t1	Lonely	1		
3	ENSDART00000001691.8	ENSDARG00000001463	tdh2	Lonely	1	Glycine, serine and threonine metabolism	KEGG
4	ENSDART00000001691.8	ENSDARG00000001463	tdh2	Lonely	1	Metabolic pathways	KEGG
5	ENSDART00000002053.11	ENSDARG00000000476	pms1_t2	27	1	DNA metabolic process	GO:BI
6	ENSDART00000002053.11	ENSDARG00000000476	pms1_t2	27	1	mismatch repair	GO:BI
7	ENSDART00000002128.7	ENSDARG00000009215	zgc:112437	47	1	Cell adhesion molecules	KEGG
8	ENSDART00000002128.7	ENSDARG00000009215	zgc:112437	47	1	Tight junction	KEGG
9	ENSDART00000002164.9	ENSDARG00000008433	unc45b_t2	Lonely	1		
10	ENSDART00000002279.8	ENSDARG00000021442	cdh11	Lonely	1		

Showing 1 to 10 of 5,916 entries Previous 1 2 3 4 5 … 592 Next

Table 10: Summary table of the workflow results

5 The results as sensitivity plots

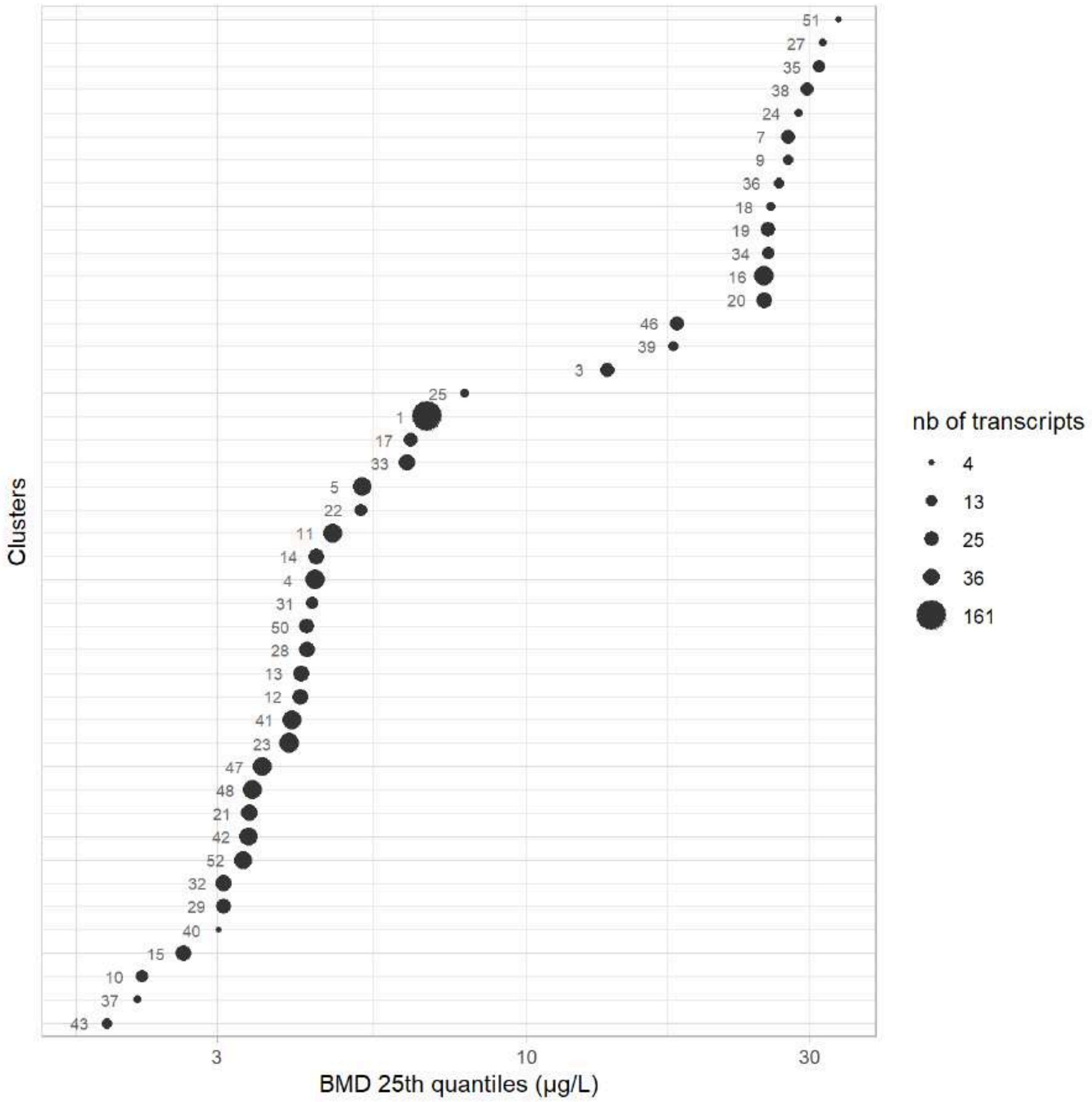

Figure 1: Sensitivity plot of clusters summarised by BMD first quartile

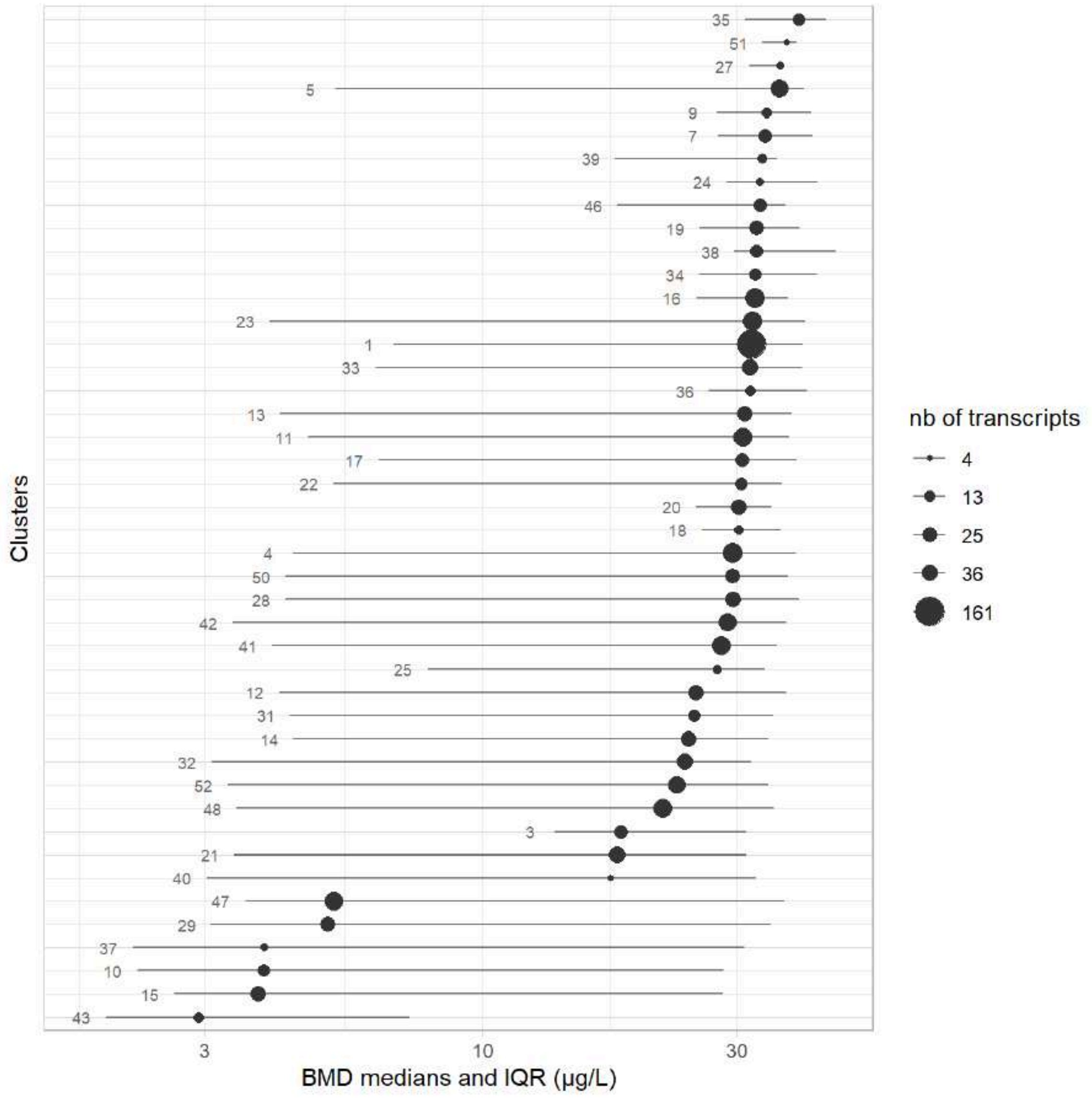

Figure 2: Sensitivity plot of clusters summarised by BMD median with the interquartile range as an interval

6 The final 44 clusters from highest to lowest sensitivity

Each cluster is represented by an interactive :

- a **curvesplot** representing the fitted curves (*with x-axis the dose in log scale and the y-axis the scaled signal*)
- a **BMDplot** representing an ECDF plot with confidence intervals on each BMD value (*with the x-axis the dose in log scale and the y-axis the empirical cumulative distribution*)
- a **table** of the cluster content with some columns of interest:
    - **id**: the gene name associated to the transcript
    - **BMD.zSD**: the computed BMD for the transcript with the color of the cell associated to the BMD value, following the *viridis* color palette.
    - **TF**: the status of the transcripts' gene being either a transcription or co-transcription factor
    - **Trend**: the shape of the dose-response curve fitted
    - **Friendliness**: the total number of clusters that the transcript is associated to

Cluster 43 - 9 transcripts (BMD q25 = 1.95 µg/L)

Driver GO terms :

KEGG pathways : Retinol metabolism

Wikipathways :

Curvesplot BMDplot

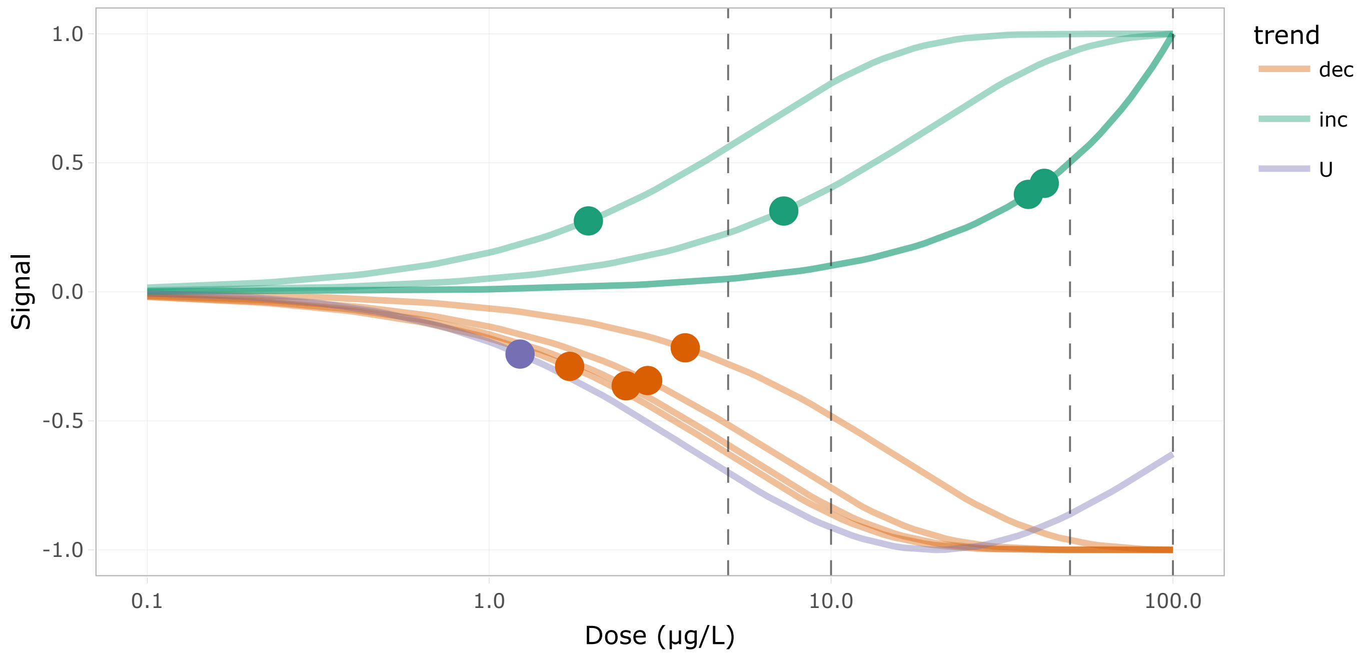

Figure 3: DR curves for the 1st cluster

Show 10 entries Search:

	id	BMD.zSD	TF	trend	friendliness
	All	All	All	All	All
1	cyp27c1	7.273	false	inc	1
2	cyp3c1_t2	42.047	false	inc	1
3	cyp3c3_g2t1	1.231	false	U	1
4	lrata	37.765	false	inc	1
5	lratb.2	1.951	false	inc	1
6	ugt1b1_t2	3.745	false	dec	1
7	ugt2a7_g2t2	2.516	false	dec	1
8	ugt2a7_g3t1	1.72	false	dec	1
9	zgc:77938	2.909	false	dec	1

Showing 1 to 9 of 9 entries Previous 1 Next

Table 11: Table of the 1st cluster content

Cluster 37 - 5 transcripts (BMD q25 = 2.19 µg/L)

Driver GO terms :

KEGG pathways :

Wikipathways :

Curvesplot BMDplot

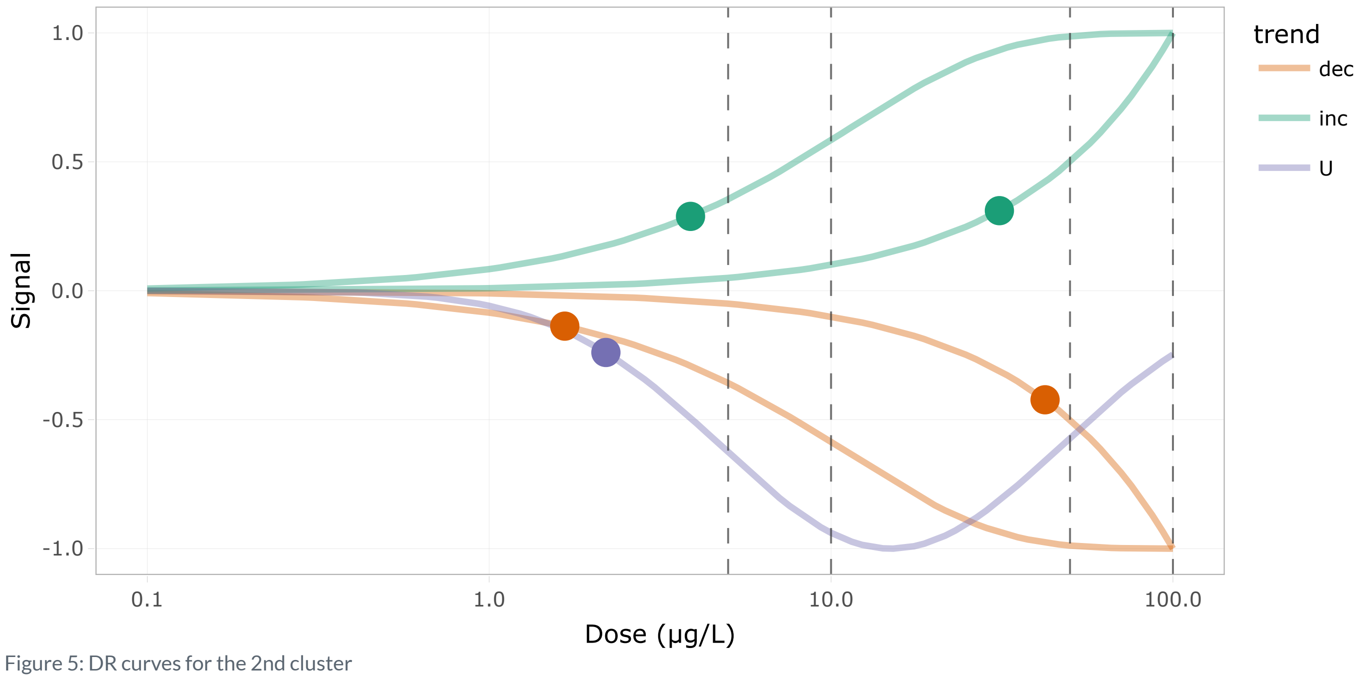

Figure 5: DR curves for the 2nd cluster

Show 10 entries Search:

	id	BMD.zSD	TF	trend	friendliness
	All	All	All	All	All
1	atg12_t2	31.073	false	inc	1
2	atg3	2.195	false	U	1
3	pi4kaa	42.264	false	dec	1
4	pik3c3_t2	3.881	false	inc	1
5	rab5ab	1.664	false	dec	1

Showing 1 to 5 of 5 entries Previous 1 Next

Table 12: Table of the 2nd cluster content

Cluster 10 - 16 transcripts (BMD q25 = 2.23 µg/L)

Driver GO terms : sphingolipid metabolic process

KEGG pathways : Sphingolipid metabolism

Wikipathways :

Curvesplot | BMDplot

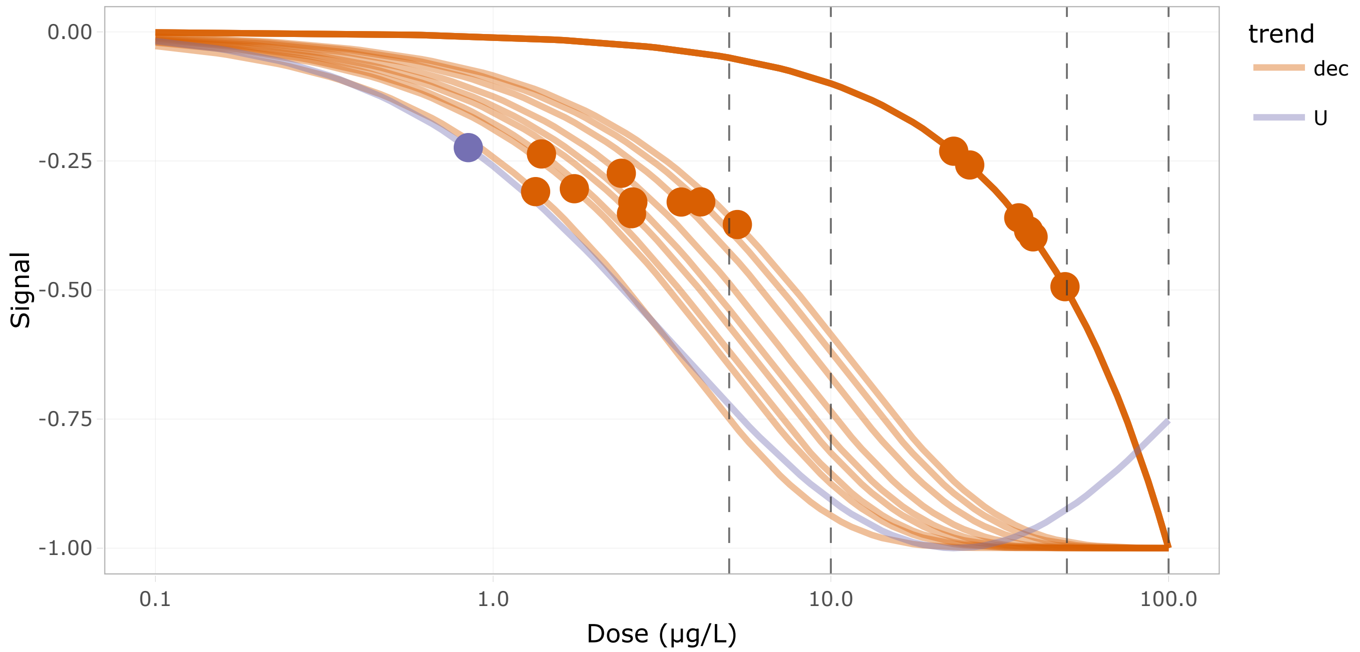

Figure 7: DR curves for the 3rd cluster

Show 10 entries

Search:

	id	BMD.zSD	TF	trend	friendliness
	All	All	All	All	All
1	SPHK1	36.011	false	dec	1
2	asah2	1.336	false	dec	1
3	b4galt6	38.515	false	dec	1
4	cers2a_t2	25.775	false	dec	1
5	cers3a_t2	1.74	false	dec	1
6	cers5	2.592	false	dec	2
7	enpp7.1_g3t1	0.844	false	U	1
8	gba2_t2	1.39	false	dec	1
9	hexb_t3	39.699	false	dec	3
10	kdsr_t2	2.396	false	dec	1

Showing 1 to 10 of 16 entries

Previous 1 2 Next

Table 13: Table of the 3rd cluster content

Cluster 15 - 31 transcripts (BMD q25 = 2.62 µg/L)

Driver GO terms : lipid biosynthetic process

KEGG pathways : Steroid biosynthesis

Wikipathways : Cholesterol biosynthesis

Curvesplot | BMDplot

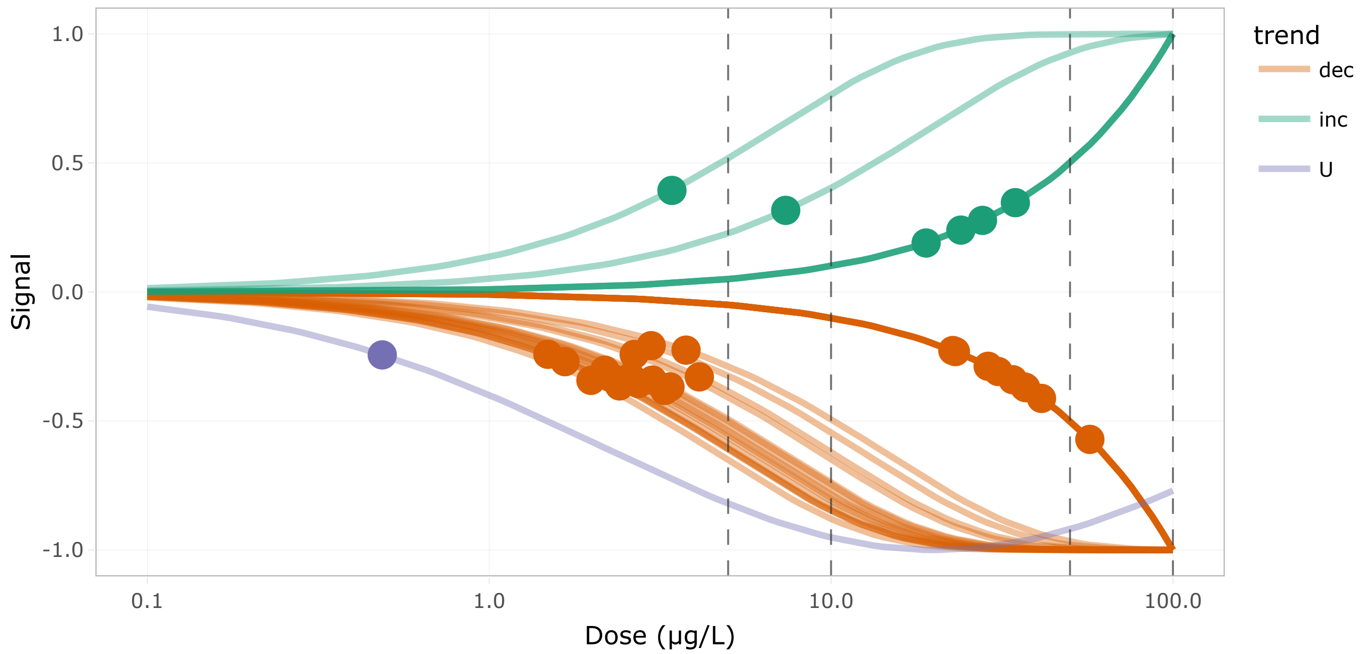

Figure 9: DR curves for the 4th cluster

Show 10 entries Search:

	id	BMD.zSD	TF	trend	friendliness
	All	All	All	All	All
1	ERG28	41.262	false	dec	1
2	PYURF_t1	7.369	false	inc	1
3	acsl1b	2.177	false	dec	3
4	alox12	3.381	false	dec	3
5	cers5	2.592	false	dec	2
6	crppa	18.967	false	inc	2
7	cyp17a1	2.731	false	dec	1
8	cyp27b1	3.005	false	dec	1
9	dhcr7	2.405	false	dec	1
10	fa2h_t3	1.666	false	dec	3

Showing 1 to 10 of 31 entries

Previous 1 2 3 4 Next

Table 14: Table of the 4th cluster content

Cluster 40 - 4 transcripts (BMD q25 = 3.01 μg/L)

Driver GO terms :

KEGG pathways :

Wikipathways :

Curvesplot | BMDplot

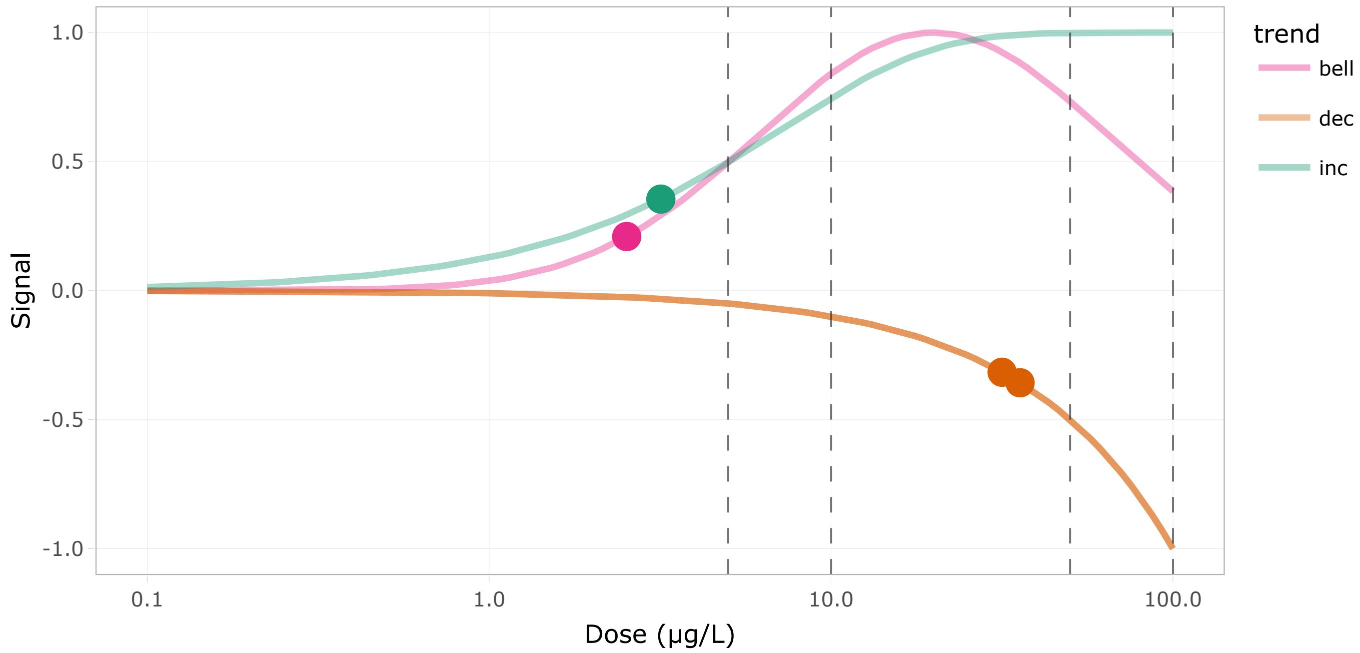

Figure 11: DR curves for the 5th cluster

Show 10 entries   Search:

	id	BMD.zSD	TF	trend	friendliness
	All	All	All	All	All
1	cbfa2t3_t2	2.525	false	bell	1
2	ldb1b_t2	31.648	true	dec	1
3	lmo1_t1	3.178	true	inc	1
4	tcf3a_t2	35.716	true	dec	1

Showing 1 to 4 of 4 entries   Previous 1 Next

Table 15: Table of the 5th cluster content

Cluster 29 - 27 transcripts (BMD q25 = 3.07 µg/L)

Driver GO terms : myofibril assembly, striated muscle contraction

KEGG pathways : Motor proteins

Wikipathways :

Curvesplot | BMDplot

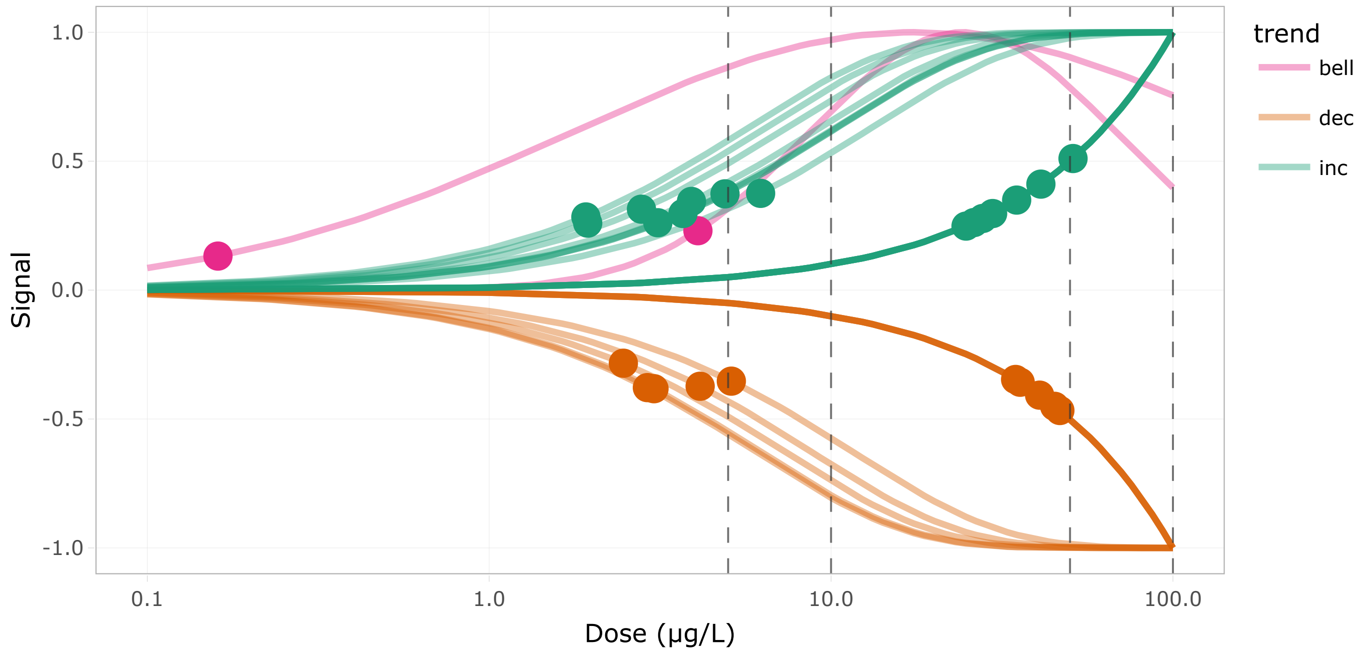

Figure 13: DR curves for the 6th cluster

Show 10 entries

Search:

	id	BMD.zSD	TF	trend	friendliness
	All	All	All	All	All
1	MYO1D	4.141	false	dec	1
2	chchd10_t2	41.092	false	inc	1
3	cx39.9	27.779	false	inc	2
4	dctn1a_t2	35.737	false	dec	2
5	dctn4_t3	4.079	false	bell	2
6	dynll2b	3.689	false	inc	3
7	erp44	3.034	false	dec	3
8	kif1b_t1	5.109	false	dec	3
9	kifc1_t2	26.202	false	inc	2
10	kifc3_t5	45.079	false	dec	2

Showing 1 to 10 of 27 entries

Previous 1 2 3 Next

Table 16: Table of the 6th cluster content

Cluster 32 - 33 transcripts (BMD q25 = 3.08 μg/L)

Driver GO terms : carboxylic acid metabolic process

KEGG pathways : Alanine, aspartate and glutamate metabolism, Biosynthesis of amino acids, Cysteine and methionine metabolism

Wikipathways :

Curvesplot BMDplot

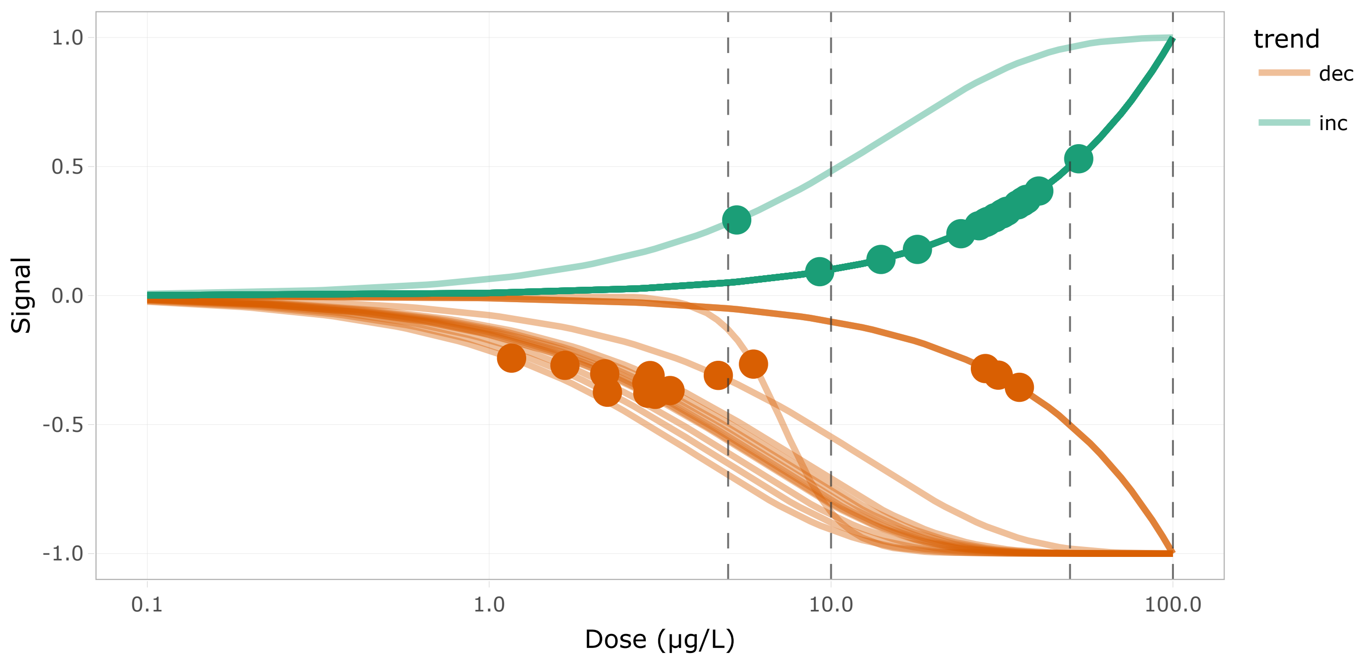

Figure 15: DR curves for the 7th cluster

Show 10 entries

Search:

	id	BMD.zSD	TF	trend	friendliness
	All	All	All	All	All
1	HPDL	28.323	false	inc	2
2	abcd1_t2	4.68	false	dec	2
3	acox3	3.06	false	dec	2
4	acsl1b	2.177	false	dec	3
5	adi1	28.749	false	inc	3
6	aldh1l1_g2t1	2.912	false	dec	2
7	alox12	3.381	false	dec	3
8	apip	37.287	false	inc	2
9	asdurf_t2	40.519	false	inc	2
10	aspa_t2	28.279	false	dec	1

Showing 1 to 10 of 33 entries

Previous 1 2 3 4 Next

Table 17: Table of the 7th cluster content

Cluster 52 - 37 transcripts (BMD q25 = 3.31 µg/L)

Driver GO terms : protein glycosylation

KEGG pathways : N-Glycan biosynthesis, Protein processing in endoplasmic reticulum, Various types of N-glycan biosynthesis

Wikipathways :

Curvesplot BMDplot

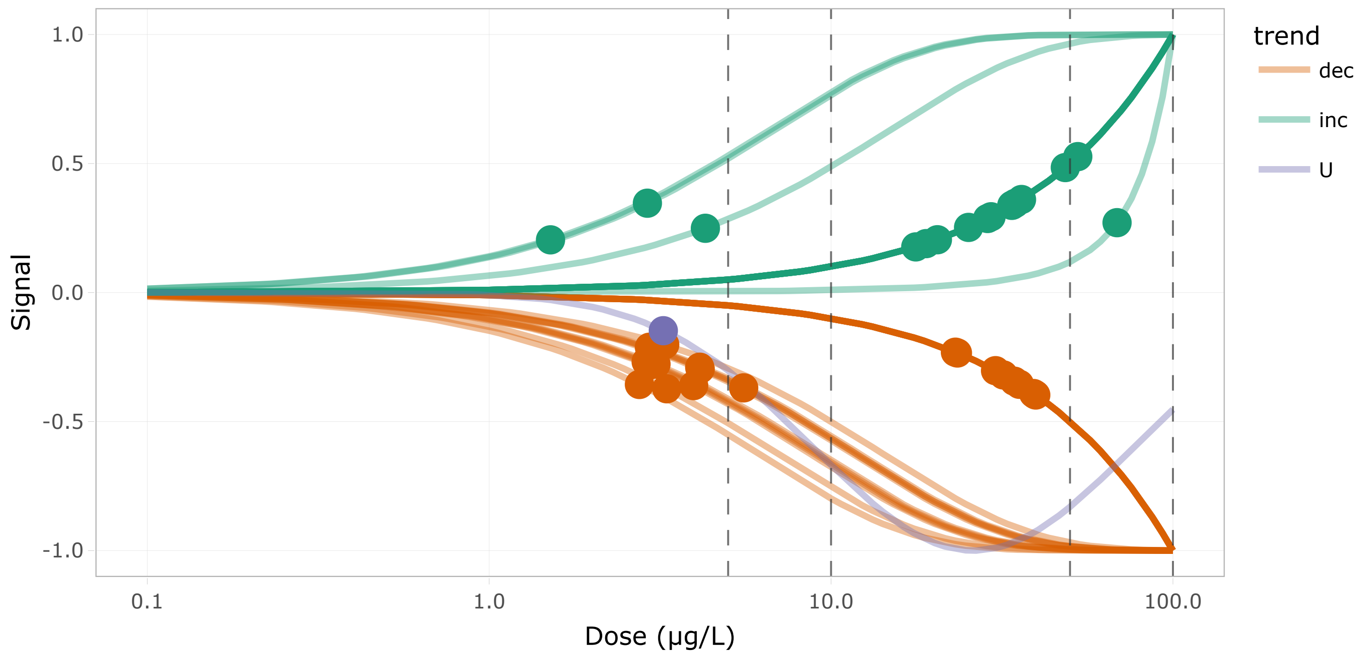

Figure 17: DR curves for the 8th cluster

Show 10 entries Search:

	id	BMD.zSD	TF	trend	friendliness
	All	All	All	All	All
1	RNF5	31.927	false	dec	4
2	alg11_t2	23.422	false	dec	1
3	atf6	30.276	true	dec	4
4	calr3b	4.129	false	dec	6
5	canx	4.142	false	dec	5
6	crppa	18.967	false	inc	2
7	dnajb11	2.879	false	dec	5
8	dnajc1	33.784	true	inc	4
9	dnajc5aa	39.493	false	dec	5
10	eif2s1b	34.593	false	inc	8

Showing 1 to 10 of 37 entries Previous 1 2 3 4 Next

Table 18: Table of the 8th cluster content

Cluster 42 - 43 transcripts (BMD q25 = 3.38 µg/L)

Driver GO terms : canonical NF-kappaB signal transduction

KEGG pathways : Apoptosis, C-type lectin receptor signaling pathway, MAPK signaling pathway

Wikipathways :

Curvesplot | BMDplot

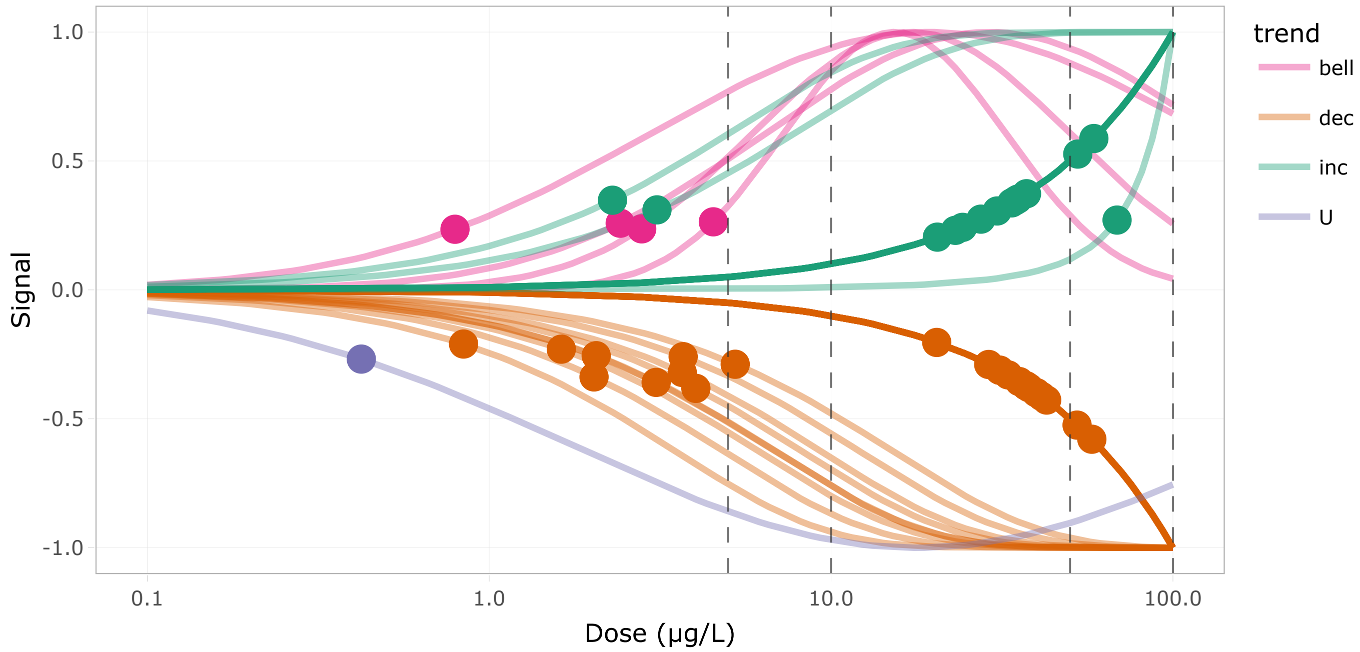

Figure 19: DR curves for the 9th cluster

Show 10 entries

Search:

	id	BMD.zSD	TF	trend	friendliness
	All	All	All	All	All
1	akt3a_t2	37.907	false	dec	3
2	apaf1_t2	0.423	false	U	2
3	arf2b_g1t2	3.693	false	dec	1
4	braf_t2	2.794	false	bell	4
5	cacng1a	30.612	false	inc	2
6	cts12	52.433	false	dec	1
7	ctsh	33.747	false	inc	1
8	ctss2.2	58.704	false	inc	1
9	cylda_t2	2.295	false	inc	3
10	eif2s1b	34.593	false	inc	8

Showing 1 to 10 of 43 entries

Previous 1 2 3 4 5 Next

Table 19: Table of the 9th cluster content

Cluster 21 - 35 transcripts (BMD q25 = 3.4 µg/L)

Driver GO terms : organic acid metabolic process

KEGG pathways : 2-Oxocarboxylic acid metabolism, Biosynthesis of amino acids, Carbon metabolism, Citrate cycle (TCA cycle), Glyoxylate and dicarboxylate metabolism

Wikipathways : TCA cycle

Curvesplot BMDplot

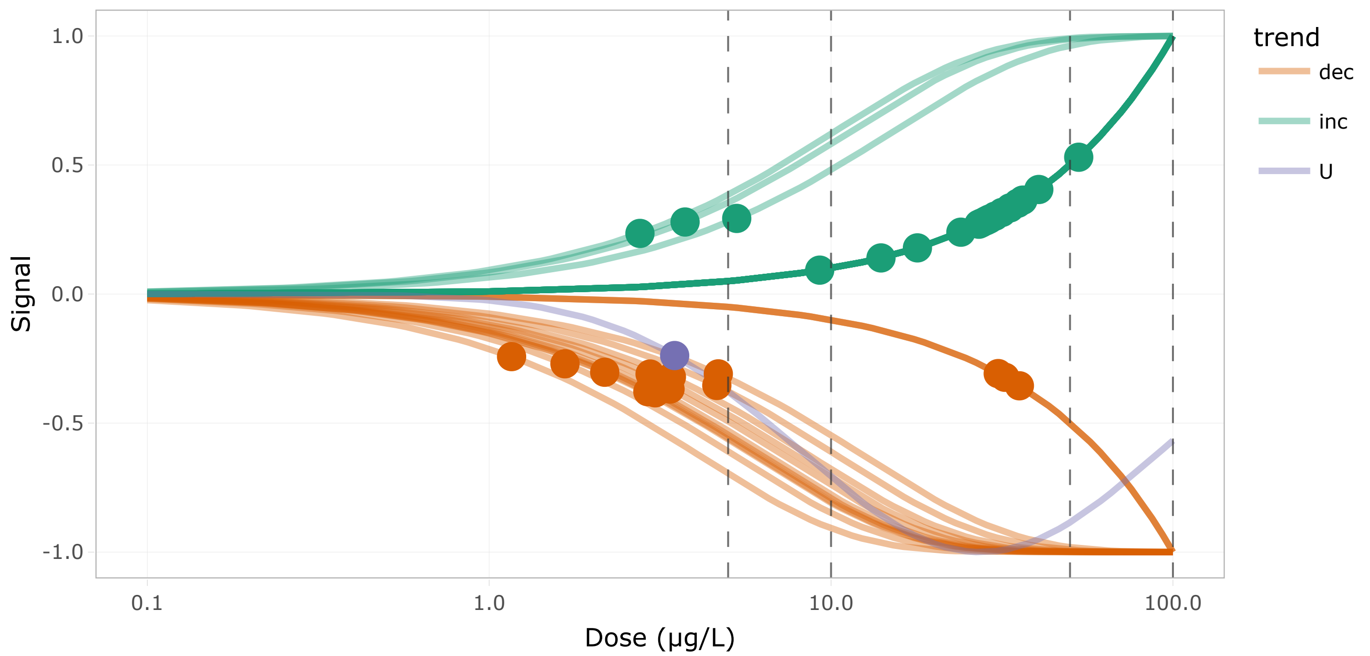

Figure 21: DR curves for the 10th cluster

Show 10 entries Search:

	id	BMD.zSD	TF	trend	friendliness
	All	All	All	All	All
1	HPDL	28.323	false	inc	2
2	abcd1_t2	4.68	false	dec	2
3	aco1	3.412	false	dec	1
4	acox3	3.06	false	dec	2
5	acsl1b	2.177	false	dec	3
6	acss2l	27.531	false	inc	1
7	adi1	28.749	false	inc	3
8	aldh1l1_g2t1	2.912	false	dec	2
9	alox12	3.381	false	dec	3
10	asdurf_t2	40.519	false	inc	2

Showing 1 to 10 of 35 entries

Previous 1 2 3 4 Next

Table 20: Table of the 10th cluster content

Cluster 48 - 50 transcripts (BMD q25 = 3.44 µg/L)

Driver GO terms : carbohydrate metabolic process

KEGG pathways : N-Glycan biosynthesis, Protein processing in endoplasmic reticulum

Wikipathways :

Curvesplot BMDplot

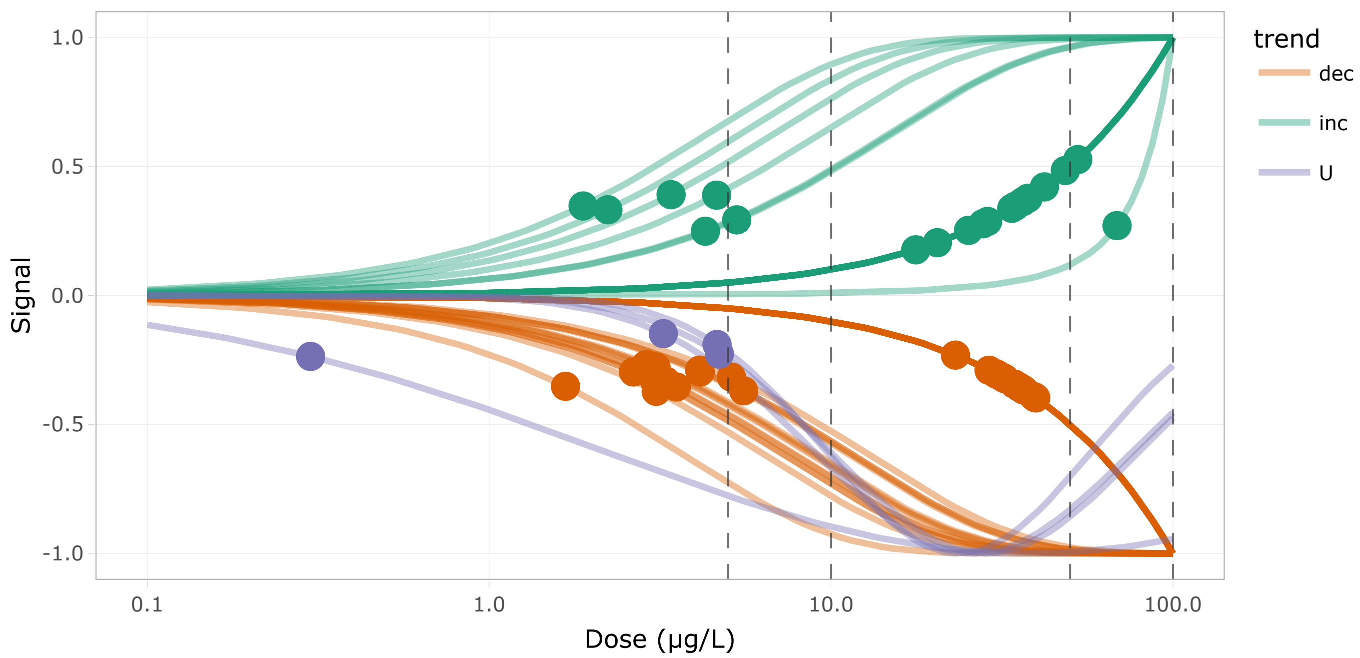

Figure 23: DR curves for the 11th cluster

Show 10 entries Search:

	id	BMD.zSD	TF	trend	friendliness
	All	All	All	All	All
1	CABZ01079192.1	1.672	false	dec	1
2	CR759923.1_t4	2.225	false	inc	1
3	CR774178.2	5.113	false	dec	1
4	GANAB_t2	2.643	false	dec	1
5	MAN1C1_t2	28.988	false	dec	1
6	RNF5	31.927	false	dec	4
7	agla_t1	3.046	false	dec	2
8	atf6	30.276	true	dec	4
9	calr3b	4.129	false	dec	6
10	canx	4.142	false	dec	5

Showing 1 to 10 of 50 entries Previous 1 2 3 4 5 Next

Table 21: Table of the 11th cluster content

Cluster 47 - 52 transcripts (BMD q25 = 3.57 µg/L)

Driver GO terms :

KEGG pathways : Regulation of actin cytoskeleton, Salmonella infection, Tight junction

Wikipathways :

Curvesplot BMDplot

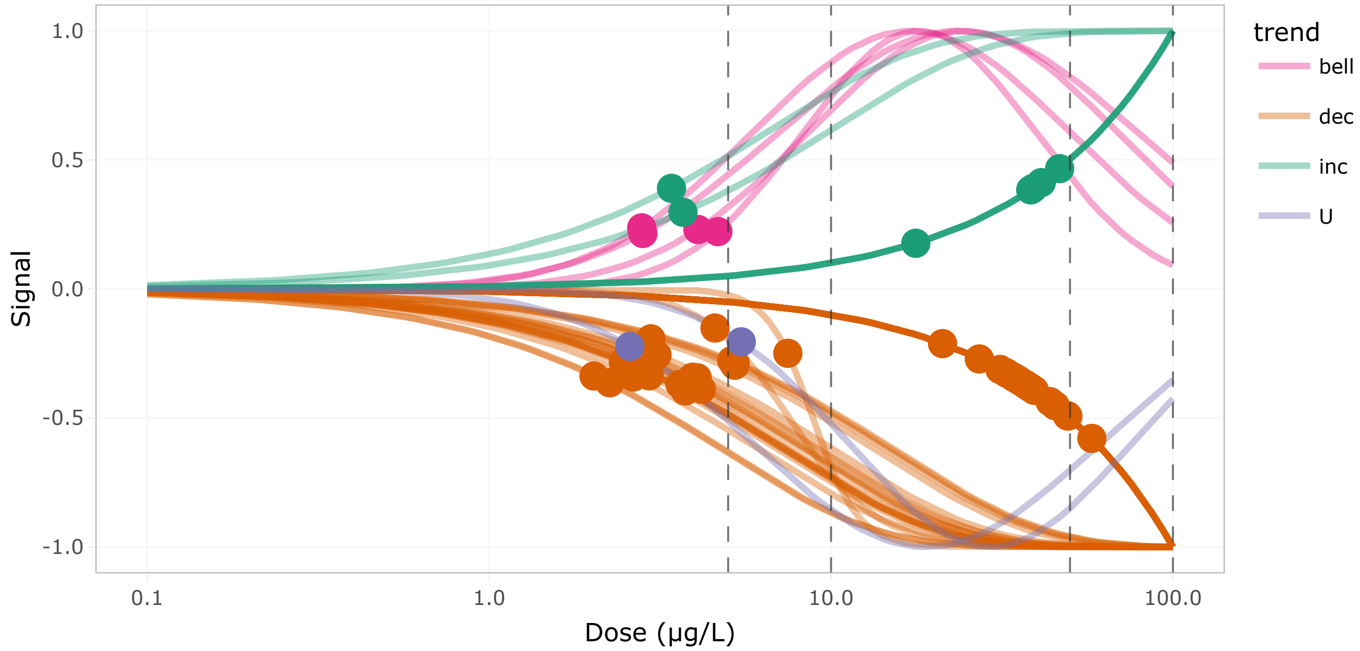

Figure 25: DR curves for the 12th cluster

Show 10 entries Search:

	id	BMD.zSD	TF	trend	friendliness
	All	All	All	All	All
1	ACBD3	2.638	false	dec	1
2	abi1b_t2	49.282	false	dec	1
3	acbd3	27.141	false	dec	1
4	actb2_t2	3.748	true	dec	1
5	actr2a	4.577	false	dec	1
6	actr2b	5.231	false	dec	1
7	afdna_t1	45.241	false	dec	2
8	akt3a_t2	37.907	false	dec	3
9	arf2a_t1	2.579	false	U	1
10	arf2a_t2	2.973	false	dec	1

Showing 1 to 10 of 52 entries

Previous 1 2 3 4 5 6 Next

Table 22: Table of the 12th cluster content

Cluster 23 - 56 transcripts (BMD q25 = 3.96 µg/L)

Driver GO terms : RNA splicing, via transesterification reactions, RNA splicing

KEGG pathways : Spliceosome

Wikipathways :

Curvesplot BMDplot

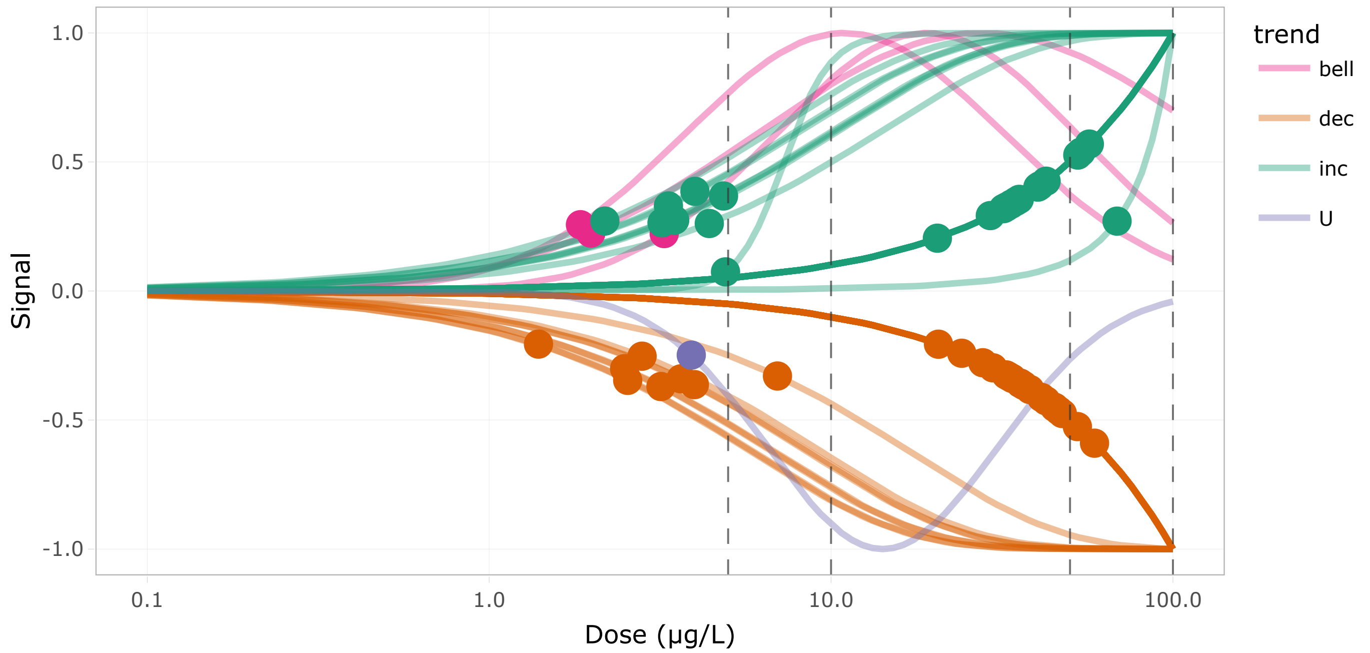

Figure 27: DR curves for the 13th cluster

Show 10 entries Search:

	id	BMD.zSD	TF	trend	friendliness
	All	All	All	All	All
1	LSM2_t4	3.18	false	dec	1
2	clk2a_t1	6.97	false	dec	2
3	ddx5_t1	2.49	true	dec	1
4	ddx5_t4	3.998	true	inc	1
5	gemin2	32.258	false	inc	3
6	gemin8_g2t1	42.542	false	dec	3
7	hmga1a_t1	3.64	true	dec	1
8	hmga1a_t2	2.801	true	dec	1
9	hmga1a_t3	4.912	true	inc	1
10	hnrnpa1b_t2	2.179	false	inc	2

Showing 1 to 10 of 56 entries

Previous 1 2 3 4 5 6 Next

Table 23: Table of the 13th cluster content

Cluster 41 - 51 transcripts (BMD q25 = 4.01 µg/L)

Driver GO terms : mRNA processing

KEGG pathways : mRNA surveillance pathway

Wikipathways : mRNA processing

Curvesplot   BMDplot

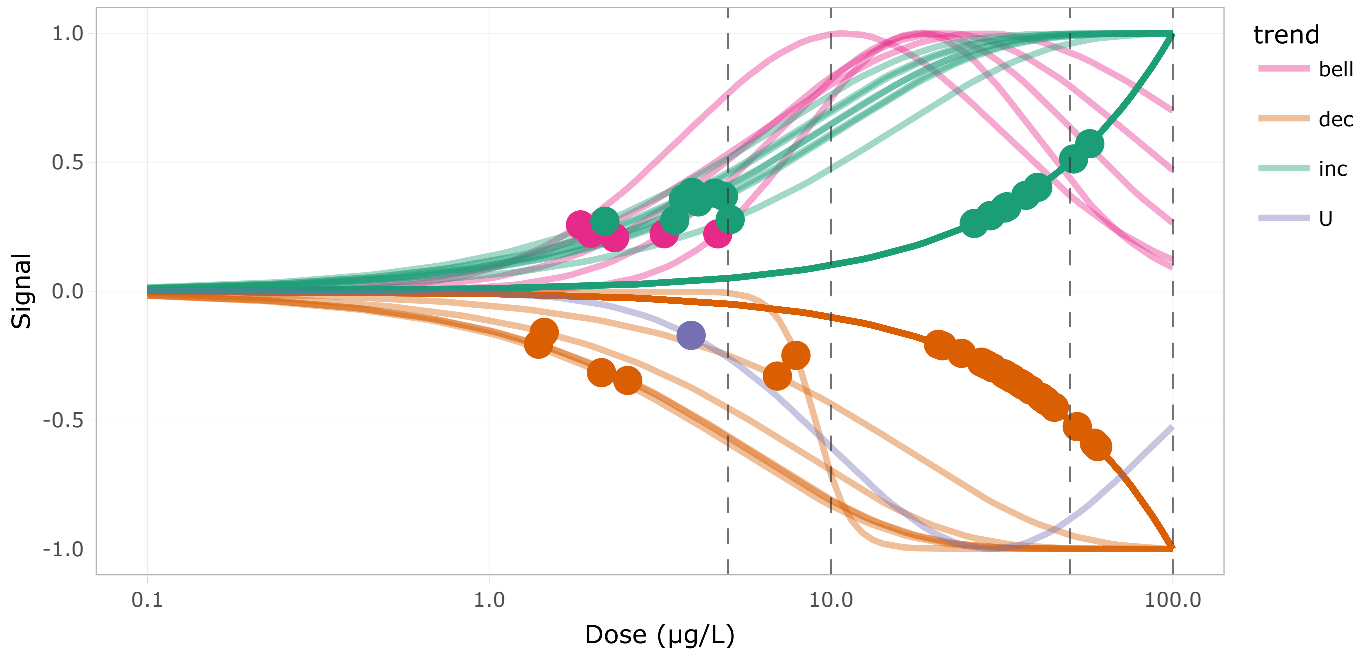

Figure 29: DR curves for the 14th cluster

Show 10 entries   Search:

	id	BMD.zSD	TF	trend	friendliness
	All	All	All	All	All
1	cdc73_t2	3.897	true	U	2
2	celf2_t2	32.673	false	dec	1
3	celf2_t4	27.548	false	dec	1
4	clk2a_t1	6.97	false	dec	2
5	cpsf3	57.18	false	inc	1
6	cpsf6_t2	2.13	false	dec	1
7	cstf2_t2	3.702	false	inc	1
8	ern1	60.306	true	dec	1
9	gemin2	32.258	false	inc	3
10	gemin8_g2t1	42.542	false	dec	3

Showing 1 to 10 of 51 entries

Previous 1 2 3 4 5 6 Next

Table 24: Table of the 14th cluster content

Cluster 12 - 30 transcripts (BMD q25 = 4.13 µg/L)

Driver GO terms : protein folding

KEGG pathways :

Wikipathways :

Curvesplot BMDplot

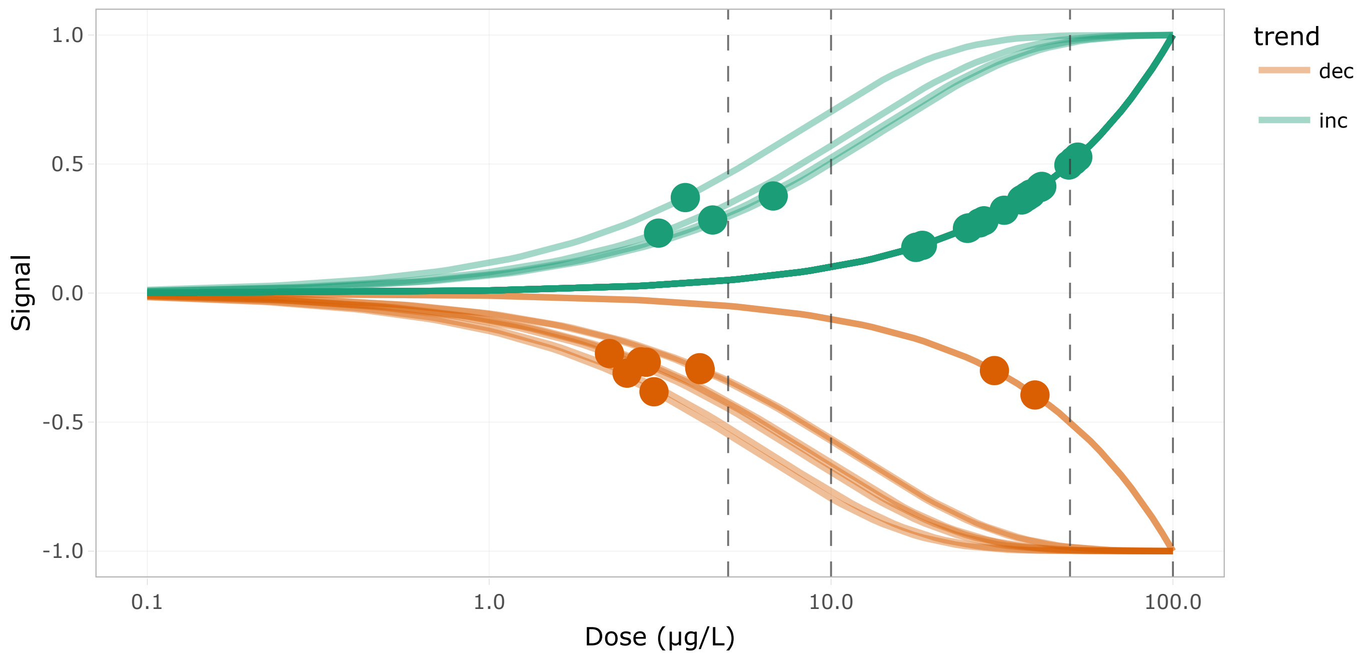

Figure 31: DR curves for the 15th cluster

Show 10 entries Search:

	id	BMD.zSD	TF	trend	friendliness
	All	All	All	All	All
1	CABZ01080568.1	17.799	false	inc	1
2	ahsa1a_t3	4.506	false	inc	2
3	calr3b	4.129	false	dec	6
4	canx	4.142	false	dec	5
5	cct2_g1t1	6.773	false	inc	1
6	cct3	36.076	false	inc	1
7	cct4	36.427	false	inc	1
8	cct5	38.401	false	inc	1
9	cct6a_t3	37.452	false	inc	1
10	cct7	49.62	false	inc	1

Showing 1 to 10 of 30 entries

Previous 1 2 3 Next

Table 25: Table of the 15th cluster content

Cluster 13 - 28 transcripts (BMD q25 = 4.15 µg/L)

Driver GO terms : translational initiation

KEGG pathways : Herpes simplex virus 1 infection

Wikipathways :

Curvesplot BMDplot

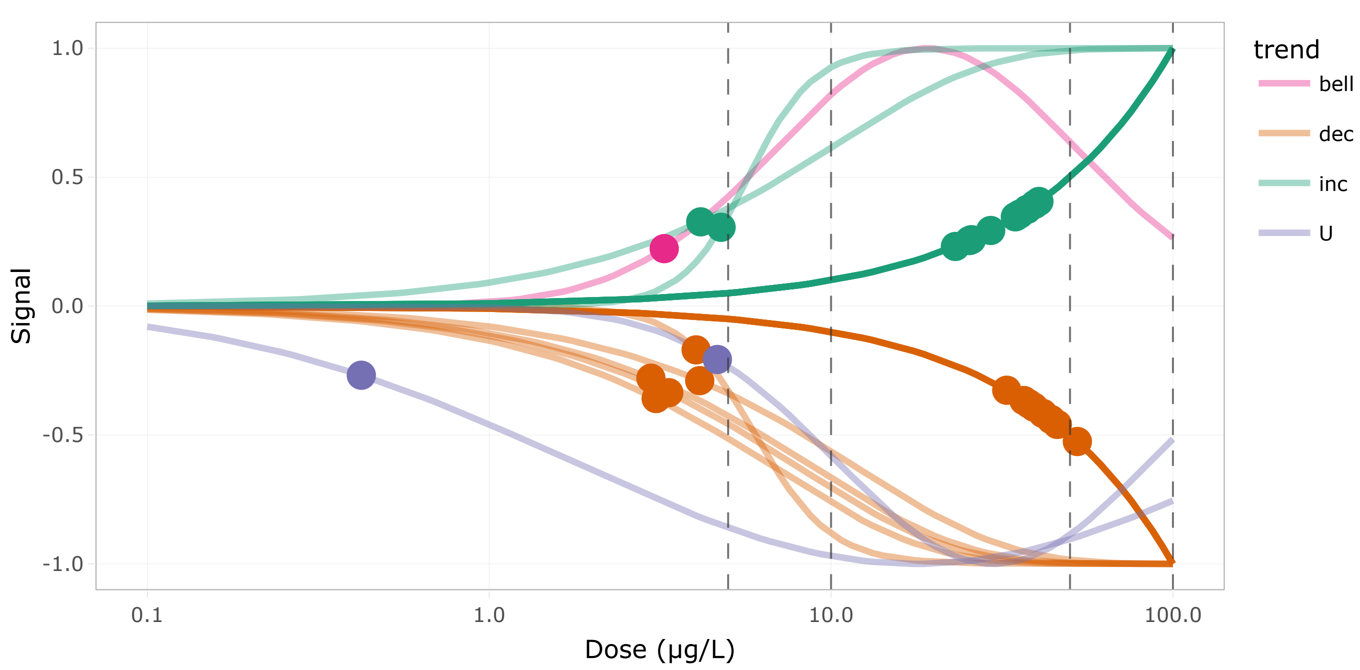

Figure 33: DR curves for the 16th cluster

Show 10 entries Search:

	id	BMD.zSD	TF	trend	friendliness
	All	All	All	All	All
1	akt3a_t2	37.907	false	dec	3
2	apaf1_t2	0.423	false	U	2
3	calr3b	4.129	false	dec	6
4	cdc123_t2	34.853	false	inc	1
5	eif2b1_t2	25.738	false	inc	1
6	eif2b3_t2	37.461	false	inc	1
7	eif2b4_t2	25.392	false	inc	1
8	eif2b5	39.412	false	inc	1
9	eif2s1b	34.593	false	inc	8

	id	BMD.zSD	TF	trend	friendliness
10	eif2s2	40.557	false	inc	1

Showing 1 to 10 of 28 entries

Previous 1 2 3 Next

Table 26: Table of the 16th cluster content

Cluster 28 - 28 transcripts (BMD q25 = 4.25 µg/L)

Driver GO terms :

KEGG pathways : Protein export, Protein processing in endoplasmic reticulum

Wikipathways :

Curvesplot BMDplot

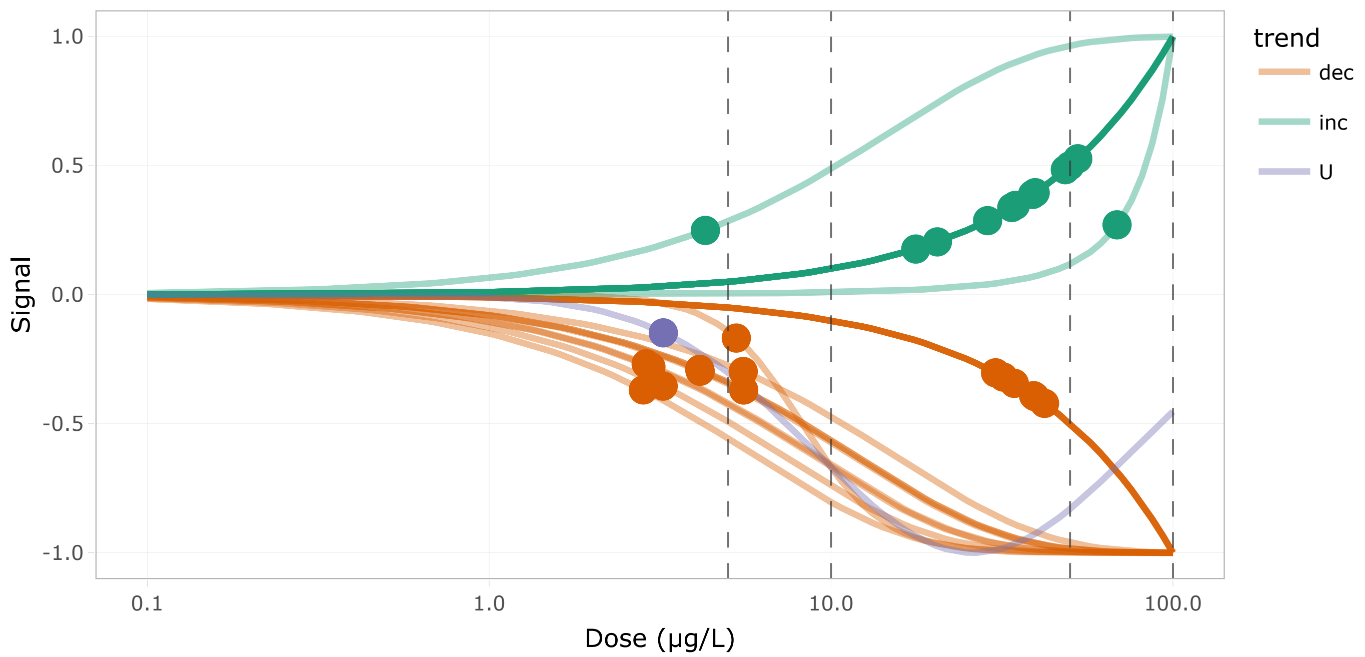

Figure 35: DR curves for the 17th cluster

Show 10 entries

Search:

	id	BMD.zSD	TF	trend	friendliness
	All	All	All	All	All
1	RNF5	31.927	false	dec	4
2	atf6	30.276	true	dec	4
3	calr3b	4.129	false	dec	6
4	canx	4.142	false	dec	5
5	dnajb11	2.879	false	dec	5
6	dnajc1	33.784	true	inc	4

	id	BMD.zSD	TF	trend	friendliness
7	dnajc5aa	39.493	false	dec	5
8	eif2s1b	34.593	false	inc	8
9	ganab	34.356	false	dec	4
10	hsp70.1_t2	52.681	false	inc	7

Showing 1 to 10 of 28 entries

Previous 1 2 3 Next

Table 27: Table of the 17th cluster content

Cluster 50 - 24 transcripts (BMD q25 = 4.25 µg/L)

Driver GO terms : COPII-coated vesicle budding

KEGG pathways : Protein processing in endoplasmic reticulum

Wikipathways :

Curvesplot BMDplot

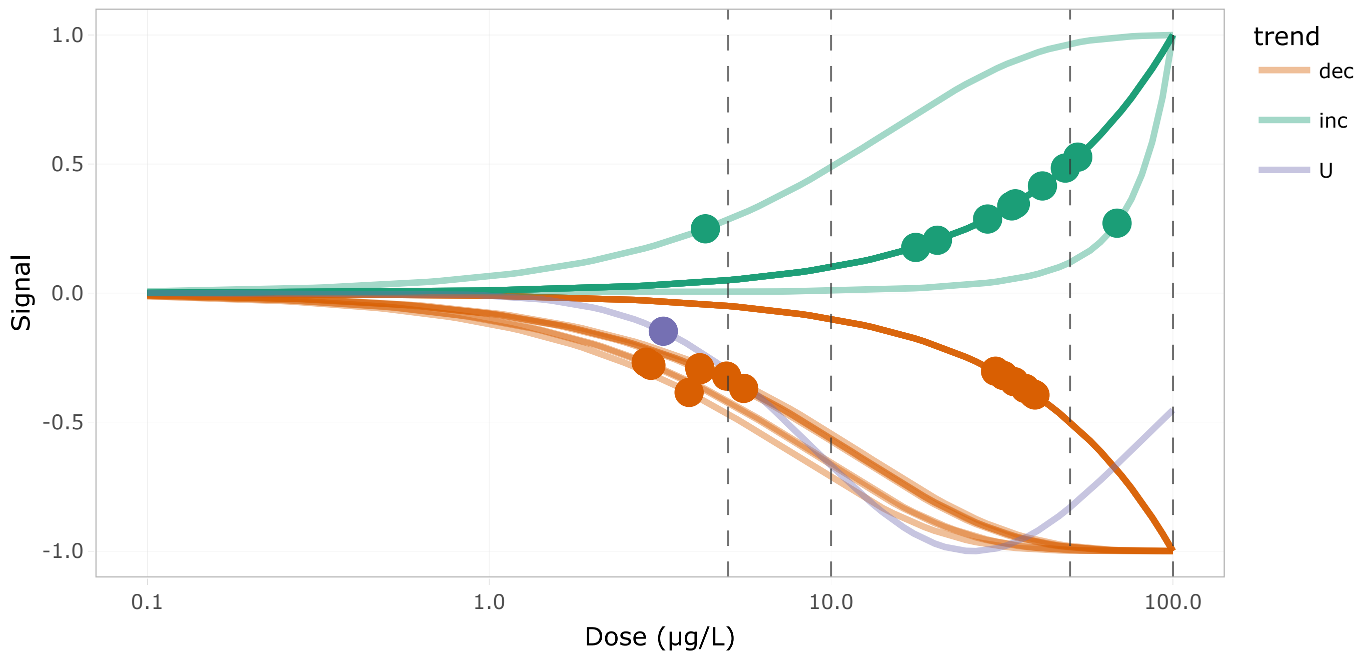

Figure 37: DR curves for the 18th cluster

Show 10 entries

Search:

	id	BMD.zSD	TF	trend	friendliness
	All	All	All	All	All
1	RNF5	31.927	false	dec	4
2	atf6	30.276	true	dec	4
3	calr3b	4.129	false	dec	6

	id	BMD.zSD	TF	trend	friendliness
4	canx	4.142	false	dec	5
5	dnajb11	2.879	false	dec	5
6	dnajc1	33.784	true	inc	4
7	dnajc5aa	39.493	false	dec	5
8	eif2s1b	34.593	false	inc	8
9	ganab	34.356	false	dec	4
10	hsp70.1_t2	52.681	false	inc	7

Showing 1 to 10 of 24 entries

Previous 1 2 3 Next

Table 28: Table of the 18th cluster content

Cluster 31 - 14 transcripts (BMD q25 = 4.33 µg/L)

Driver GO terms :

KEGG pathways : ATP-dependent chromatin remodeling

Wikipathways :

Curvesplot BMDplot

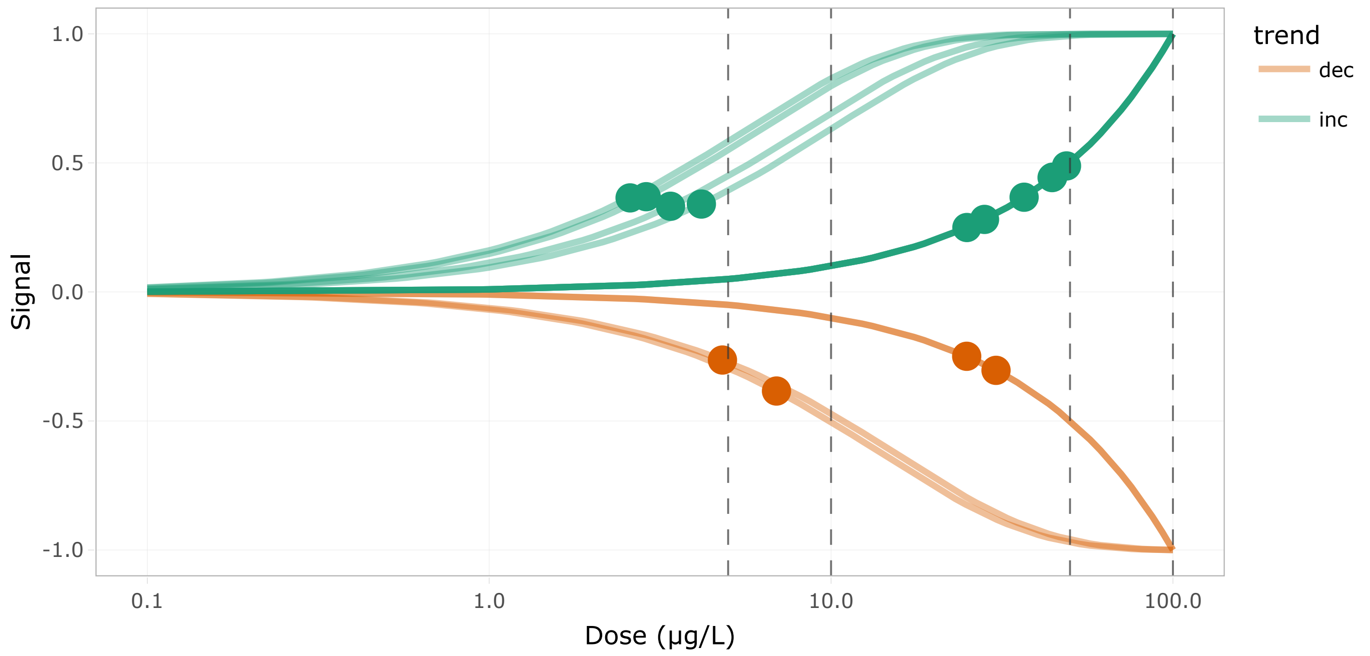

Figure 39: DR curves for the 19th cluster

Show 10 entries

Search:

	id	BMD.zSD	TF	trend	friendliness
	All	All	All	All	All
1	actr8	44.489	true	inc	1
2	brd8_t3	6.925	false	dec	1
3	brd8_t4	4.175	false	inc	1
4	chaf1a_g2t2	4.812	false	dec	1
5	chd3_g2t2	30.379	true	dec	1
6	chrac1_t2	24.966	true	inc	1
7	ep400_t2	3.392	true	inc	1
8	gatad2b	24.931	true	dec	1
9	h2ax	28.11	false	inc	1
10	lin52_t2	44.316	false	inc	1

Showing 1 to 10 of 14 entries

Previous 1 2 Next

Table 29: Table of the 19th cluster content

Cluster 4 - 54 transcripts (BMD q25 = 4.39 µg/L)

Driver GO terms : ATP synthesis coupled electron transport, generation of precursor metabolites and energy

KEGG pathways : Oxidative phosphorylation, Cardiac muscle contraction

Wikipathways : Electron transport chain, Oxidative phosphorylation

Curvesplot BMDplot

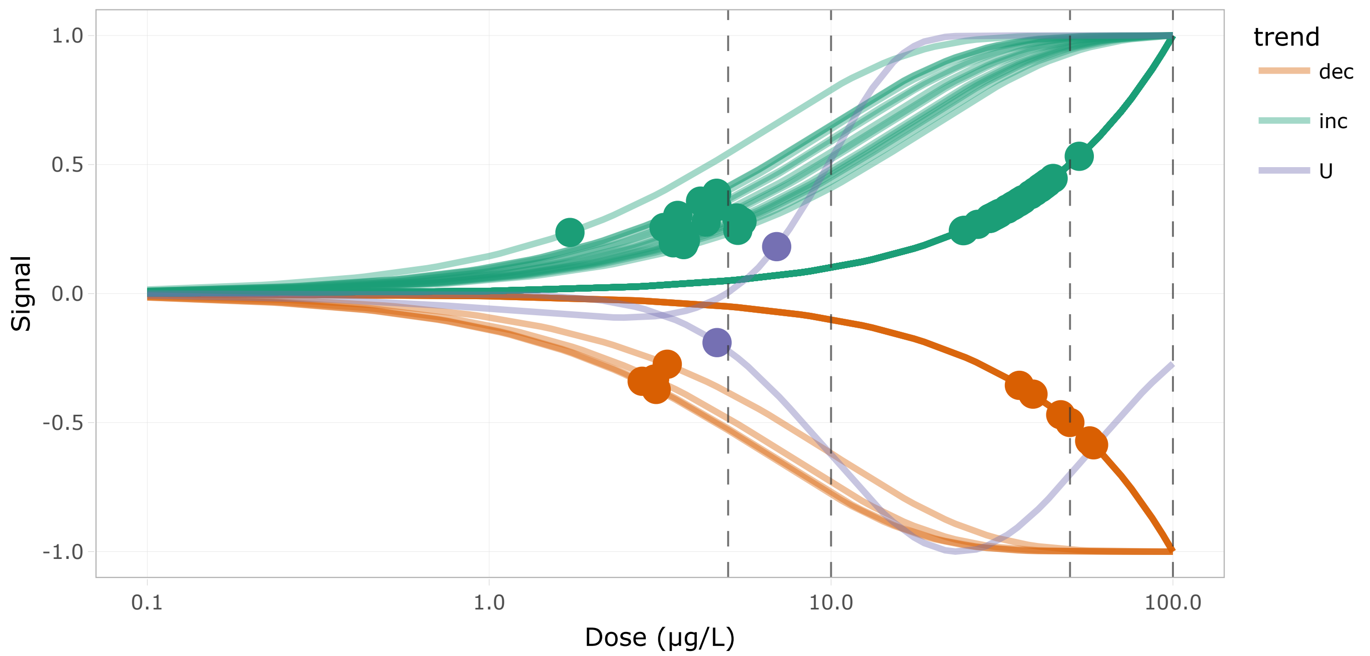

Figure 41: DR curves for the 20th cluster

Show 10 entries

Search:

	id	BMD.zSD	TF	trend	friendliness
	All	All	All	All	All
1	CABZ01102240.1_t1	4.152	false	inc	1
2	agla_t1	3.046	false	dec	2
3	atp1a3a	58.597	false	dec	1
4	atp2a1_t1	42.978	false	inc	2
5	atp2a2b	3.318	false	dec	1
6	atp2a3_t3	2.802	false	dec	1
7	atp5f1c	24.517	false	inc	1
8	cacng1a	30.612	false	inc	2
9	coa6	4.642	false	inc	1
10	cox11_t2	32.759	false	inc	1

Showing 1 to 10 of 54 entries

Previous 1 2 3 4 5 6 Next

Table 30: Table of the 20th cluster content

Cluster 14 - 30 transcripts (BMD q25 = 4.4 µg/L)

Driver GO terms : muscle system process

KEGG pathways : Vascular smooth muscle contraction

Wikipathways :

Curvesplot BMDplot

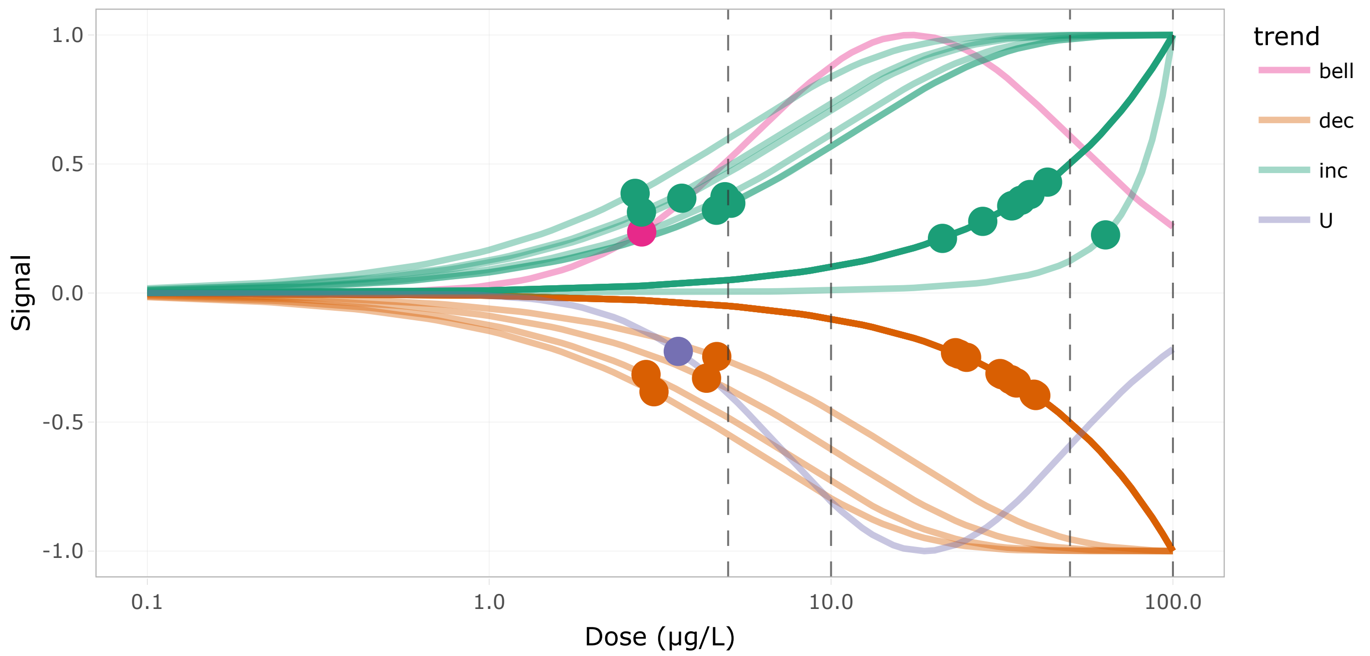

Figure 43: DR curves for the 21th cluster

Show 10 entries

Search:

	id	BMD.zSD	TF	trend	friendliness
	All	All	All	All	All
1	BX936363.1	33.564	false	dec	1
2	adma	38.185	false	inc	1
3	atp2a1_t1	42.978	false	inc	2
4	braf_t2	2.794	false	bell	4
5	cald1a	4.632	false	dec	1
6	cald1b_t2	23.697	false	dec	1
7	cx39.9	27.779	false	inc	2
8	erp44	3.034	false	dec	3
9	foxo3b	39.206	true	dec	1
10	gna11b	39.71	false	dec	1

Showing 1 to 10 of 30 entries

Previous 1 2 3 Next

Table 31: Table of the 21th cluster content

Cluster 11 - 52 transcripts (BMD q25 = 4.69 µg/L)

Driver GO terms :

KEGG pathways : Adherens junction, Mitophagy - animal, Ribosome biogenesis in eukaryotes, Wnt signaling pathway

Wikipathways :

Curvesplot BMDplot

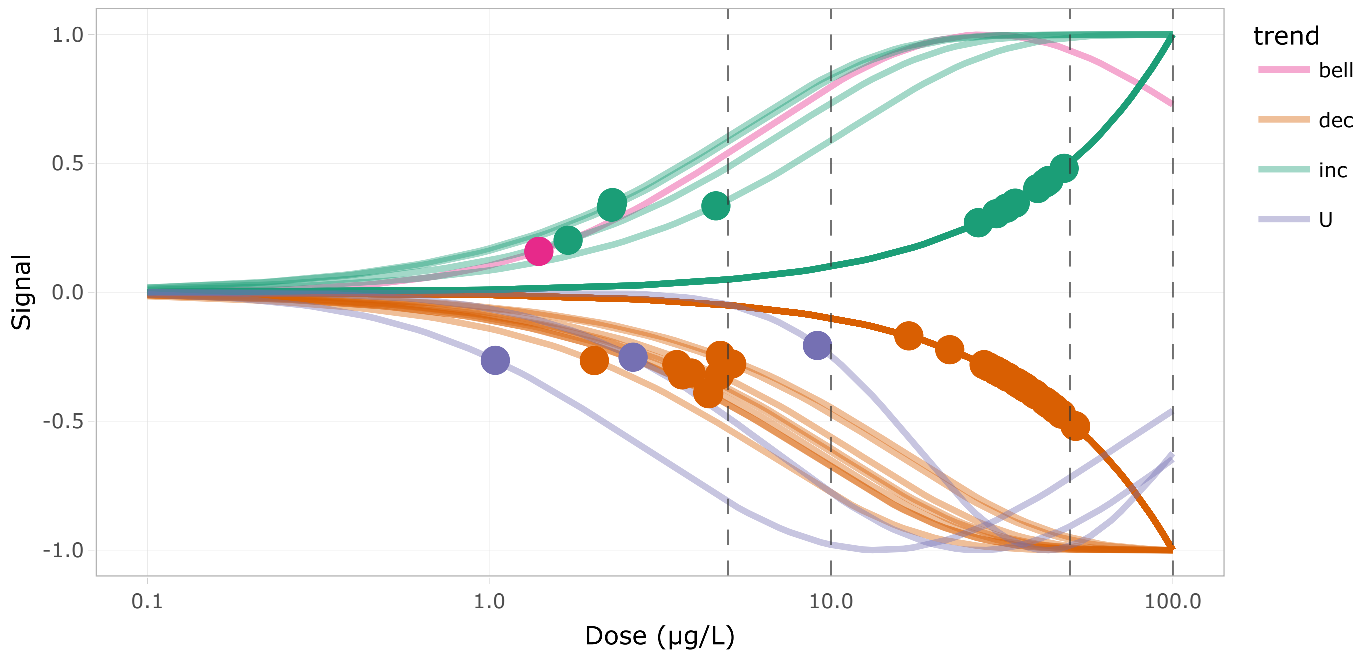

Figure 45: DR curves for the 22th cluster

Show 10 entries Search:

	id	BMD.zSD	TF	trend	friendliness
	All	All	All	All	All
1	UTP14C	51.973	false	dec	3
2	acp1_t1	32.62	false	inc	1
3	afdna_t1	45.241	false	dec	2
4	atg9a_t4	43.399	false	inc	1
5	bcl9_t2	44.947	true	dec	2
6	cacybp	26.991	false	inc	1
7	cdh1_t2	4.36	false	dec	2
8	clul1_g2t1	39.766	false	dec	1
9	csnk1g2a_t2	30.313	false	dec	1
10	csnk2a2a	4.606	false	inc	1

Showing 1 to 10 of 52 entries Previous 1 2 3 4 5 6 Next

Table 32: Table of the 22th cluster content

Cluster 22 - 15 transcripts (BMD q25 = 5.23 µg/L)

Driver GO terms : polyamine metabolic process

KEGG pathways : Arginine and proline metabolism

Wikipathways :

Curvesplot BMDplot

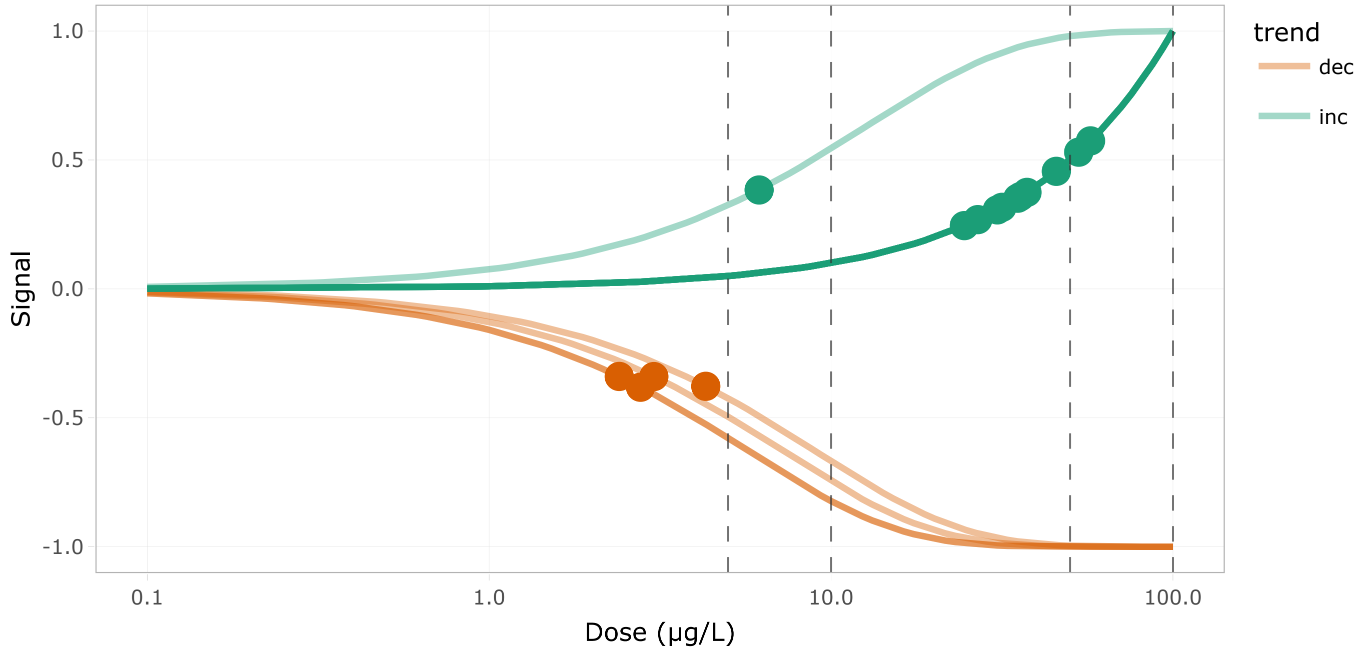

Figure 47: DR curves for the 23th cluster

Show 10 entries Search:

	id	BMD.zSD	TF	trend	friendliness
	All	All	All	All	All
1	agmat	2.771	false	dec	1
2	aldh2.1	57.408	false	inc	1
3	aldh3a2b	3.032	false	dec	1
4	arg2	24.542	false	inc	1
5	azin1b	53.019	false	inc	4
6	ckmb	30.64	false	inc	1
7	mao	2.401	false	dec	1
8	oat_t2	35.164	false	inc	3
9	oaz2a	4.302	false	dec	1
10	oaz2b	45.589	false	inc	1

Showing 1 to 10 of 15 entries Previous 1 2 Next

Table 33: Table of the 23th cluster content

Cluster 5 - 45 transcripts (BMD q25 = 5.27 µg/L)

Driver GO terms : protein catabolic process

KEGG pathways : Proteasome

Wikipathways :

Curvesplot BMDplot

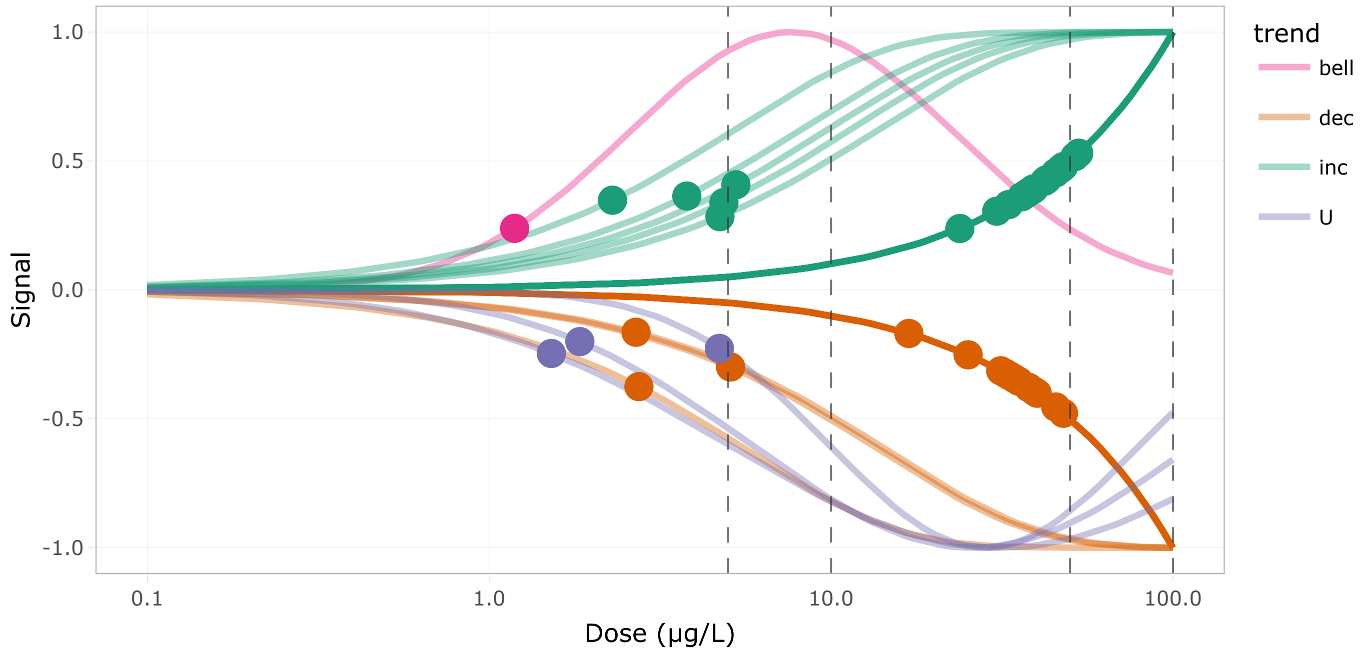

Figure 49: DR curves for the 24th cluster

Show 10 entries Search:

	id	BMD.zSD	TF	trend	friendliness
	All	All	All	All	All
1	CR788324.2	33.242	false	dec	1
2	anapc4_t2	4.732	false	inc	3
3	appbp2	40.076	false	dec	1
4	arih2_t1	2.689	false	dec	1
5	azin1b	53.019	false	inc	4
6	cul2	30.52	false	inc	2
7	cylda_t2	2.295	false	inc	3
8	desi1a	5.268	false	inc	1
9	fbxo33	37.759	false	dec	1
10	hectd3_t2	2.742	false	dec	1

Showing 1 to 10 of 45 entries

Previous 1 2 3 4 5 Next

Table 34: Table of the 24th cluster content

Cluster 33 - 32 transcripts (BMD q25 = 6.28 µg/L)

Driver GO terms : microtubule-based process

KEGG pathways :

Wikipathways :

Curvesplot | BMDplot

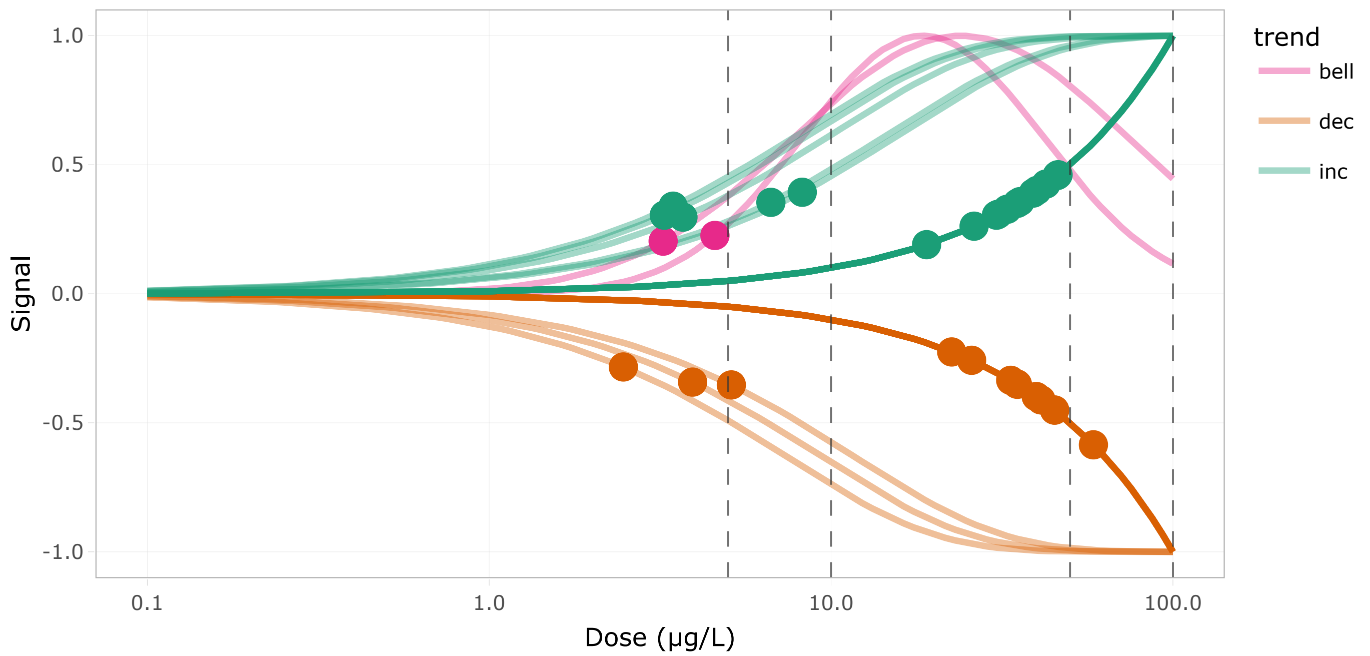

Figure 51: DR curves for the 25th cluster

Show 10 entries

Search:

	id	BMD.zSD	TF	trend	friendliness
	All	All	All	All	All
1	CU929259.1	22.537	false	dec	1
2	cep192_t2	40.049	false	inc	1
3	cep57	3.253	false	inc	1
4	cfap298	19.032	false	inc	1
5	ckap5_t2	8.236	false	inc	1
6	cpeb2_t1	3.228	false	bell	1
7	cpeb3	34.885	false	dec	1
8	dynll2b	3.689	false	inc	3
9	fyco1a_t2	3.935	false	dec	2
10	gas2l1_t2	25.772	false	dec	1

Showing 1 to 10 of 32 entries

Previous 1 2 3 4 Next

Table 35: Table of the 25th cluster content

Cluster 17 - 22 transcripts (BMD q25 = 6.37 µg/L)

Driver GO terms :

KEGG pathways : Ubiquitin mediated proteolysis

Wikipathways :

Curvesplot | BMDplot

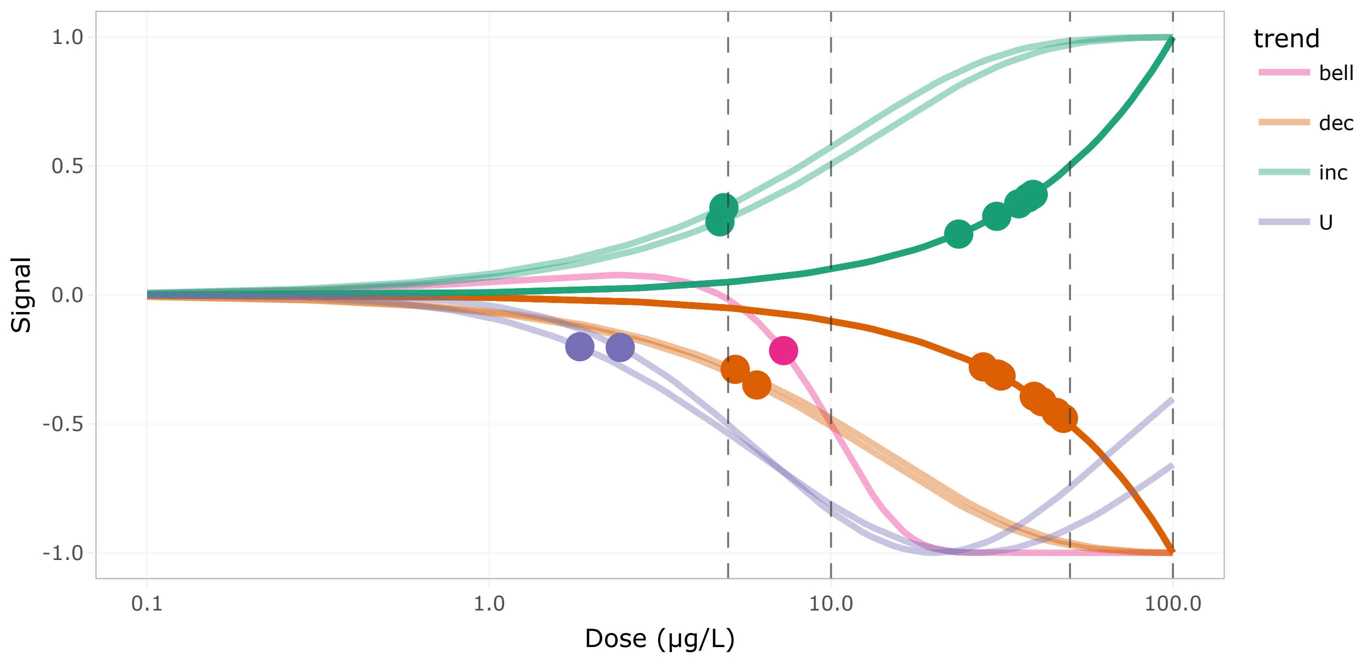

Figure 53: DR curves for the 26th cluster

Show 10 entries | Search:

	id	BMD.zSD	TF	trend	friendliness
	All	All	All	All	All
1	UBE2M_t4	35.376	false	inc	1
2	UBE2M_t5	30.787	false	dec	1
3	anapc4_t2	4.732	false	inc	3
4	anapc5	23.627	false	inc	2
5	cbl	41.388	true	dec	1
6	cdc34b	31.425	false	dec	1
7	cul2	30.52	false	inc	2
8	itcha	47.842	false	dec	2
9	itchb	39.285	true	dec	2
10	map3k1	5.243	false	dec	3

Showing 1 to 10 of 22 entries

Previous 1 2 3 Next

Table 36: Table of the 26th cluster content

Cluster 1 - 161 transcripts (BMD q25 = 6.79 µg/L)

Driver GO terms : embryo development ending in birth or egg hatching, myeloid cell differentiation, ribonucleoprotein complex biogenesis, translation, ribosomal small subunit assembly

KEGG pathways : Ribosome

Wikipathways : Cytoplasmic ribosomal proteins

Curvesplot | BMDplot

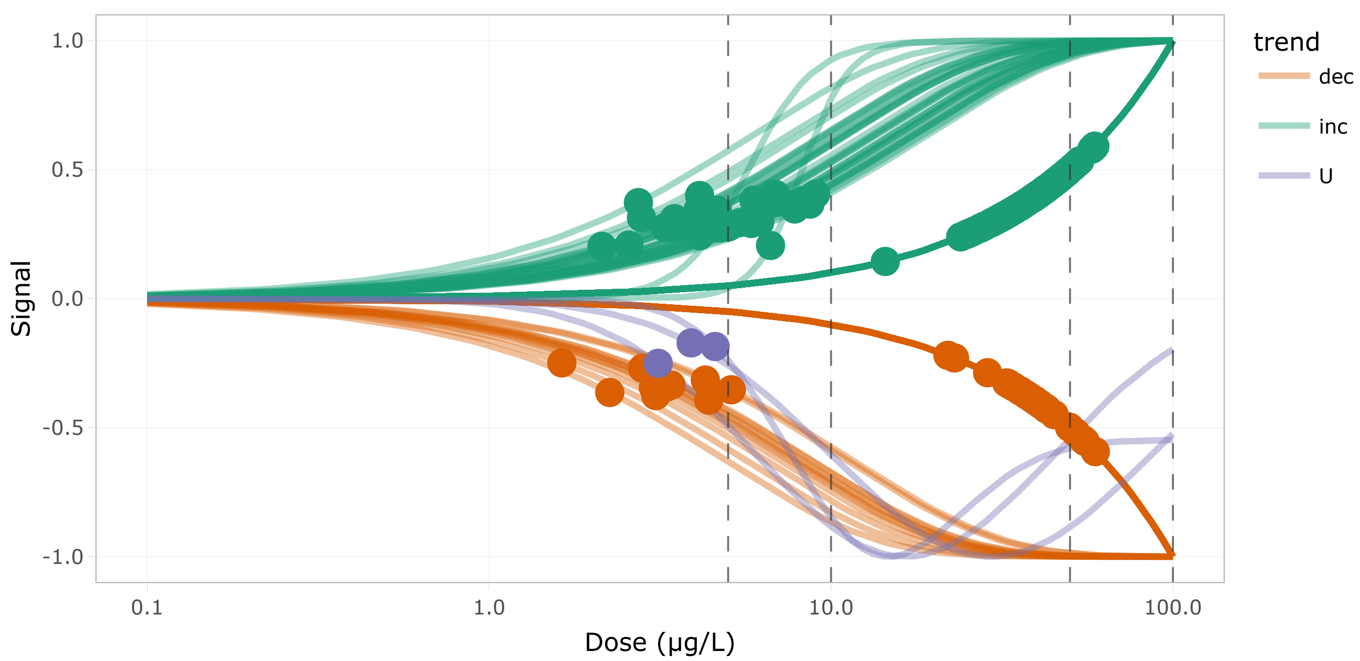

Figure 55: DR curves for the 27th cluster

Show 10 entries | Search:

	id	BMD.zSD	TF	trend	friendliness
	All	All	All	All	All
1	UTP14C	51.973	false	dec	3
2	aars1_g2t2	14.411	false	inc	2
3	ahsa1a_t3	4.506	false	inc	2
4	bcl9_t2	44.947	true	dec	2
5	btf3	27.546	false	inc	1
6	btf3l4	25.572	false	inc	1
7	cdc73_t2	3.897	true	U	2
8	chd7_t3	54.656	true	dec	1
9	cnbpa_t1	47.732	false	inc	1
10	cnot1_g2t1	39.652	false	dec	1

Showing 1 to 10 of 161 entries

Previous 1 2 3 4 5 … 17 Next

Table 37: Table of the 27th cluster content

Cluster 25 - 6 transcripts (BMD q25 = 7.85 µg/L)

Driver GO terms :

KEGG pathways :

Wikipathways :

Curvesplot BMDplot

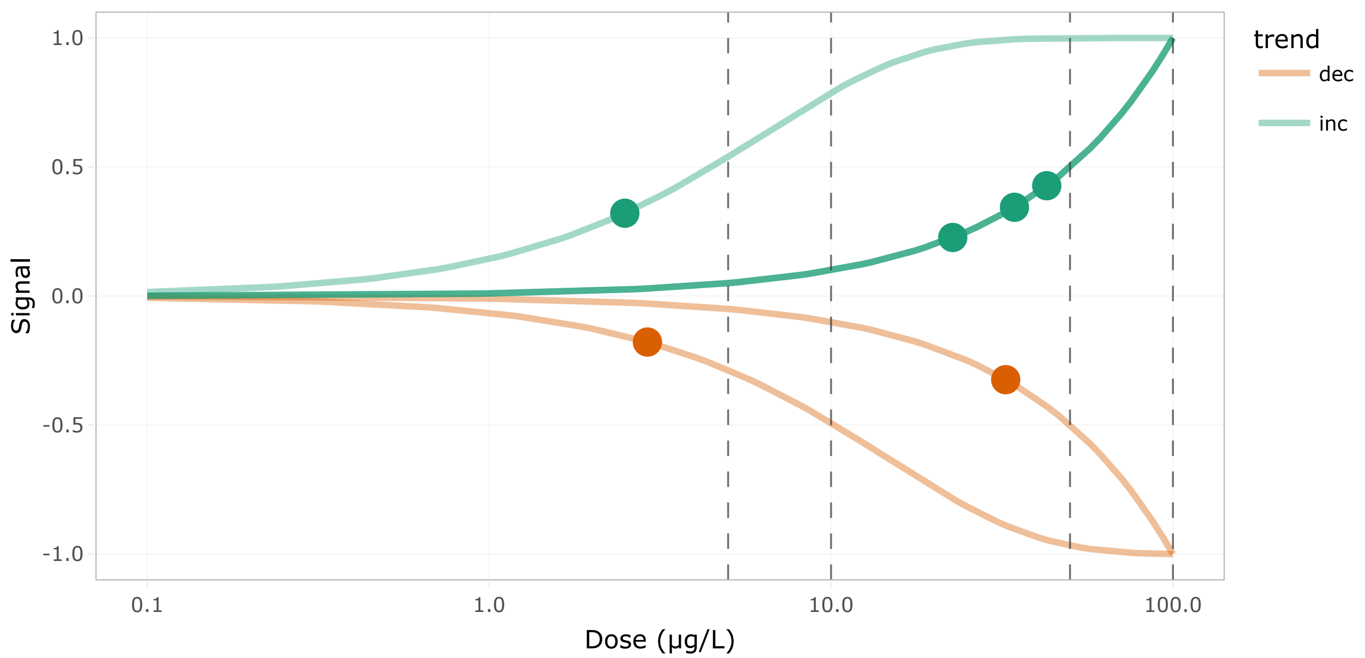

Figure 57: DR curves for the 28th cluster

Show 10 entries Search:

	id	BMD.zSD	TF	trend	friendliness
	All	All	All	All	All
1	bms1_g2t1	2.903	false	dec	1
2	glmna	34.388	false	inc	1
3	ncoa6_t2	2.494	false	inc	1
4	ncoa6_t3	32.422	false	dec	1
5	ngdn	42.746	false	inc	1
6	pno1	22.695	false	inc	1

Showing 1 to 6 of 6 entries Previous 1 Next

Table 38: Table of the 28th cluster content

Cluster 3 - 23 transcripts (BMD q25 = 13.65 µg/L)

Driver GO terms : tRNA aminoacylation

KEGG pathways : Aminoacyl-tRNA biosynthesis

Wikipathways :

Curvesplot BMDplot

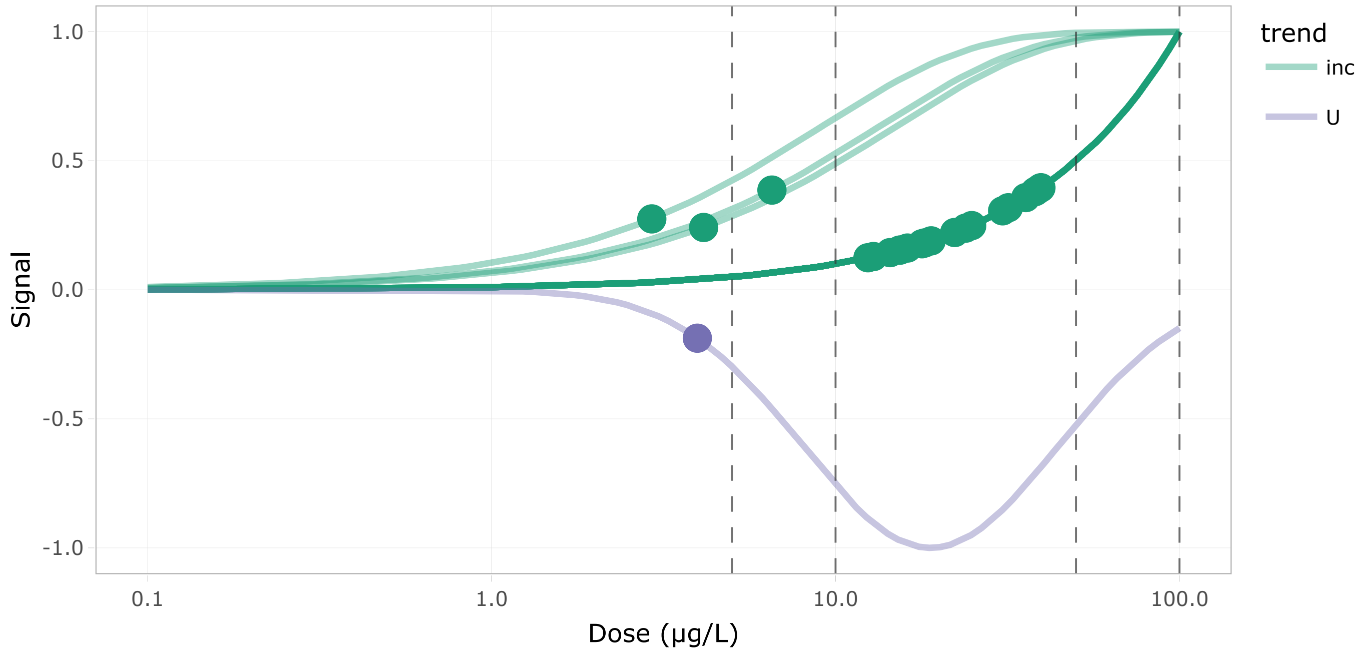

Figure 59: DR curves for the 29th cluster

Show 10 entries Search:

	id	BMD.zSD	TF	trend	friendliness
	All	All	All	All	All
1	LO017852.1	6.532	false	inc	1
2	WARS1	39.552	false	inc	1
3	aars1_g1t2	15.339	false	inc	1
4	aars1_g2t2	14.411	false	inc	2
5	aimp1a	35.732	false	inc	1
6	aimp1b_t4	4.136	false	inc	1
7	aimp2_t2	38.006	false	inc	1
8	dars1	17.833	false	inc	1
9	eprs1_t2	15.442	false	inc	1
10	farsa	16.181	false	inc	1

Showing 1 to 10 of 23 entries Previous 1 2 3 Next

Table 39: Table of the 29th cluster content

Cluster 39 - 7 transcripts (BMD q25 = 17.7 µg/L)

Driver GO terms : mitotic chromosome condensation

KEGG pathways :

Wikipathways :

Curvesplot | BMDplot

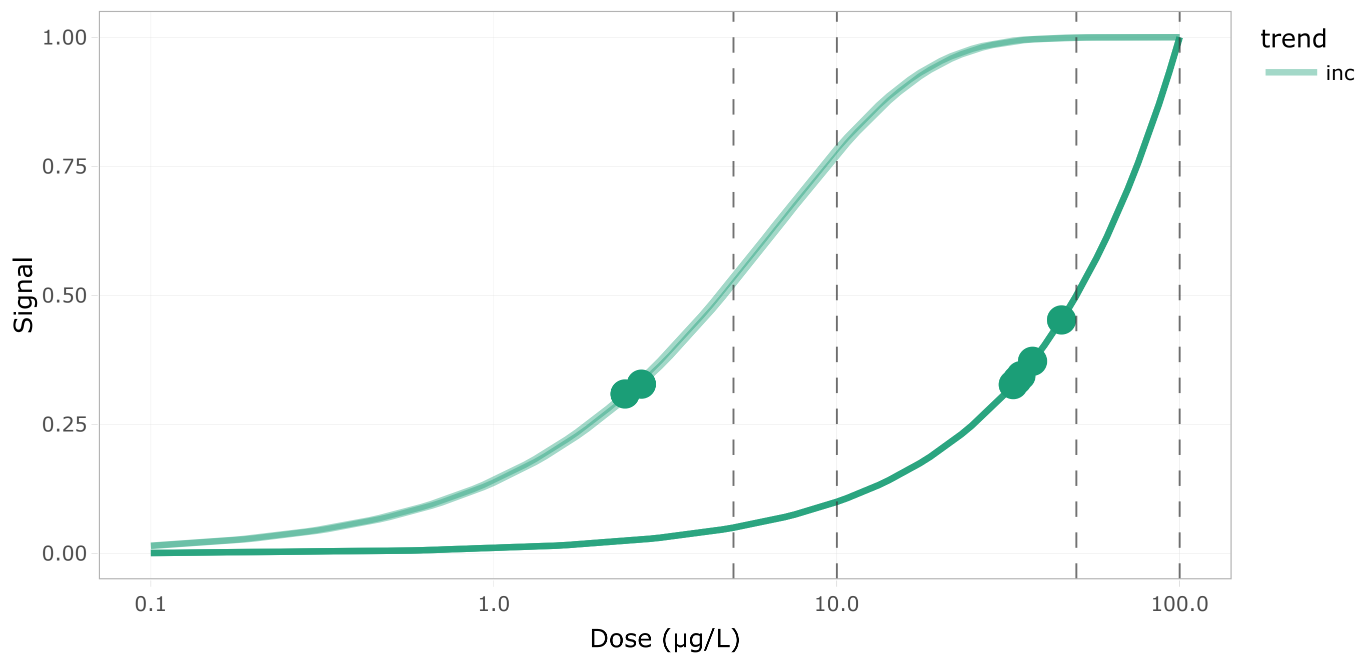

Figure 61: DR curves for the 30th cluster

Show 10 entries   Search:

	id	BMD.zSD	TF	trend	friendliness
	All	All	All	All	All
1	ncapd3_g1t1	2.693	false	inc	1
2	ncapd3_g2t1	45.242	false	inc	1
3	ncapg2_t2	37.233	false	inc	1
4	ncapg_g1t2	2.413	false	inc	1
5	ncapg_g3t1	32.715	false	inc	1
6	ncaph_g2t1	33.606	false	inc	1
7	smc2	34.447	false	inc	1

Showing 1 to 7 of 7 entries   Previous 1 Next

Table 40: Table of the 30th cluster content

Cluster 46 - 21 transcripts (BMD q25 = 17.89 µg/L)

Driver GO terms : DNA alkylation, methylation

KEGG pathways : Cysteine and methionine metabolism

Wikipathways :

Curvesplot BMDplot

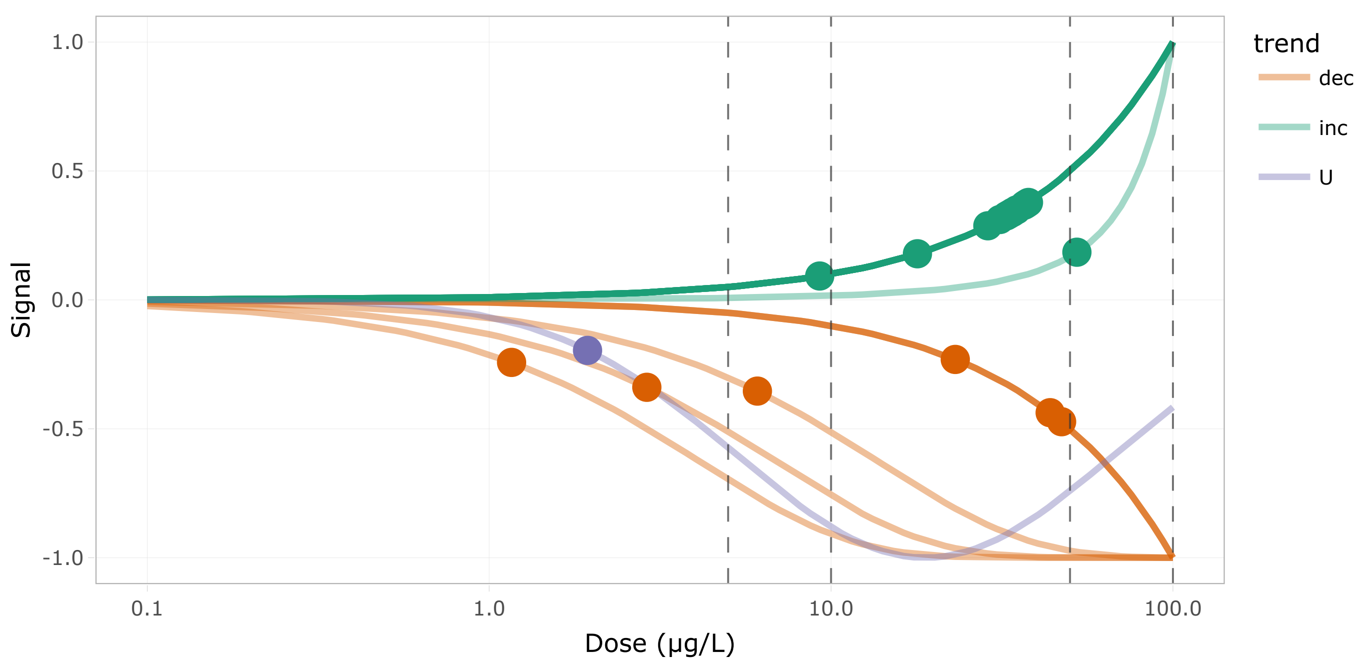

Figure 63: DR curves for the 31th cluster

Show 10 entries Search:

	id	BMD.zSD	TF	trend	friendliness
	All	All	All	All	All
1	adi1	28.749	false	inc	3
2	apip	37.287	false	inc	2
3	dnmt1	34.061	true	inc	1
4	dnmt3aa_t2	43.738	false	dec	1
5	dnmt3ab_t2	23.084	false	dec	1
6	dnmt3bb.1_g2t1	6.089	false	dec	2
7	dnmt3bb.3_g1t2	36.719	false	inc	2
8	dot1l_t3	47.243	false	dec	3
9	gclm	2.892	false	dec	2
10	mto1	52.371	false	inc	1

Showing 1 to 10 of 21 entries Previous 1 2 3 Next

Table 41: Table of the 31th cluster content

Cluster 20 - 31 transcripts (BMD q25 = 25.13 µg/L)

Driver GO terms : DNA geometric change, DNA replication

KEGG pathways : Cell cycle, DNA replication

Wikipathways : Cell cycle, DNA replication, G1 to S cell cycle control

Curvesplot | BMDplot

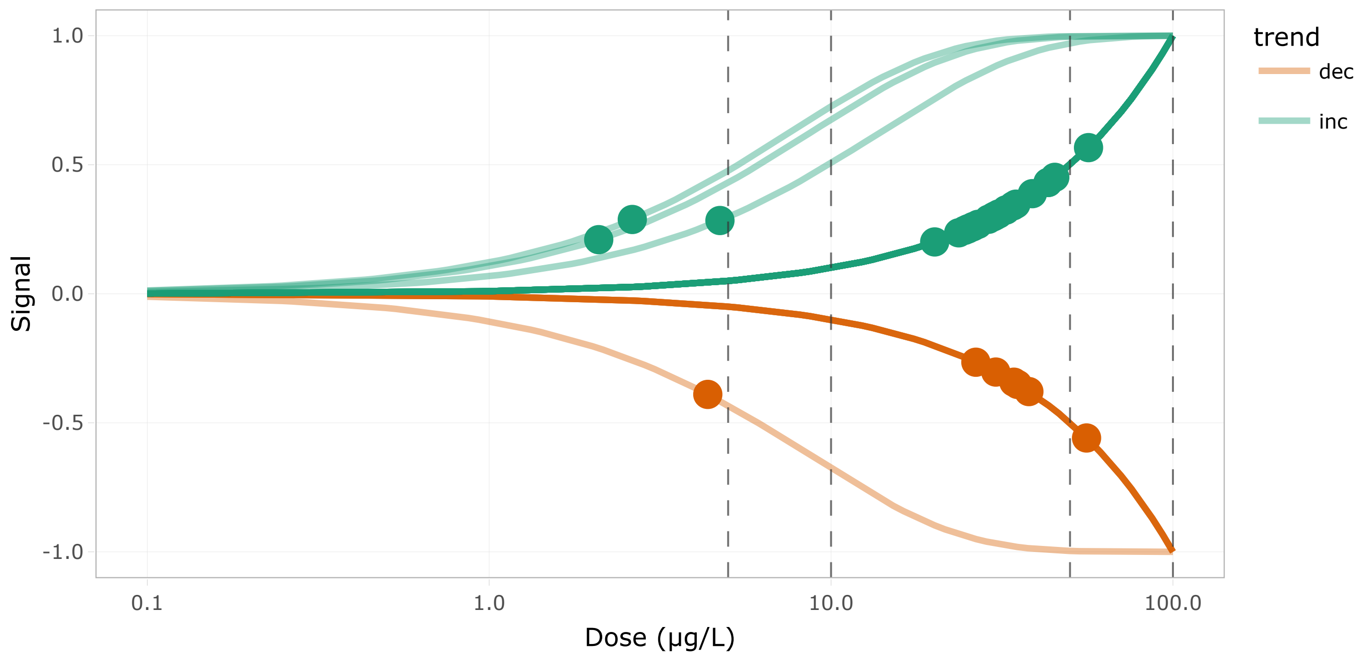

Figure 65: DR curves for the 32th cluster

Show 10 entries | Search:

	id	BMD.zSD	TF	trend	friendliness
	All	All	All	All	All
1	anapc4_t2	4.732	false	inc	3
2	anapc5	23.627	false	inc	2
3	atrx_g2t1	55.886	true	dec	2
4	ccnb1_t2	24.594	false	inc	2
5	ccne2_t3	32.539	false	inc	2
6	cdh1_t2	4.36	false	dec	2
7	cdk2	25.841	true	inc	2
8	cdkn1ca	45.124	false	inc	2
9	cenps_g2t1	34.058	false	inc	2
10	dna2	34.726	false	inc	2

Showing 1 to 10 of 31 entries

Previous 1 2 3 4 Next

Table 42: Table of the 32th cluster content

Cluster 16 - 54 transcripts (BMD q25 = 25.18 µg/L)

Driver GO terms : DNA metabolic process, DNA recombination

KEGG pathways : DNA replication, Base excision repair, Mismatch repair, Nucleotide excision repair, Fanconi anemia pathway, Homologous recombination

Wikipathways : DNA replication, G1 to S cell cycle control

Curvesplot   BMDplot

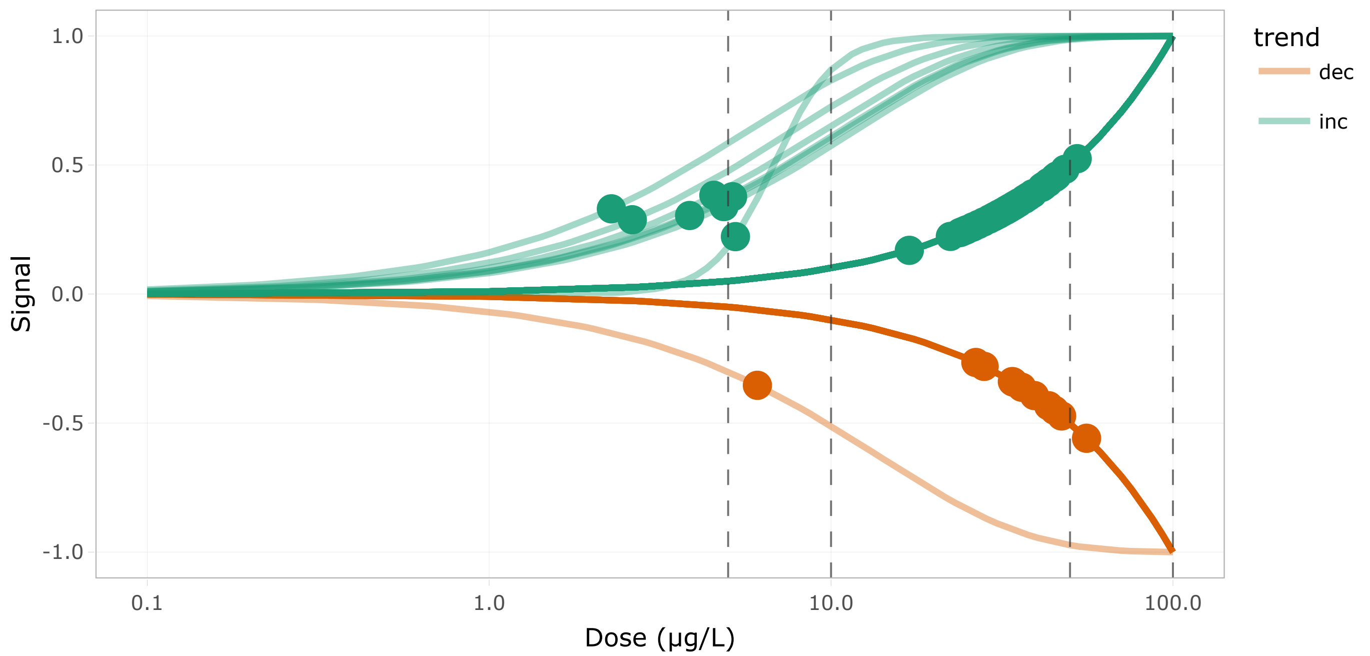

Figure 67: DR curves for the 33th cluster

Show 10 entries   Search:

	id	BMD.zSD	TF	trend	friendliness
	All	All	All	All	All
1	FO834799.1	5.151	false	inc	1
2	atrx_g2t1	55.886	true	dec	2
3	brca2	16.94	true	inc	1
4	ccdc36	4.542	false	inc	1
5	ccnb1_t2	24.594	false	inc	2
6	ccne2_t3	32.539	false	inc	2
7	cdca7a	31.664	false	inc	1
8	cdk2	25.841	true	inc	2
9	cdkn1ca	45.124	false	inc	2
10	cenps_g2t1	34.058	false	inc	2

Showing 1 to 10 of 54 entries   Previous 1 2 3 4 5 6 Next

Table 43: Table of the 33th cluster content

Cluster 34 - 15 transcripts (BMD q25 = 25.53 µg/L)

Driver GO terms :

KEGG pathways : Nucleotide metabolism, Pyrimidine metabolism

Wikipathways :

Curvesplot BMDplot

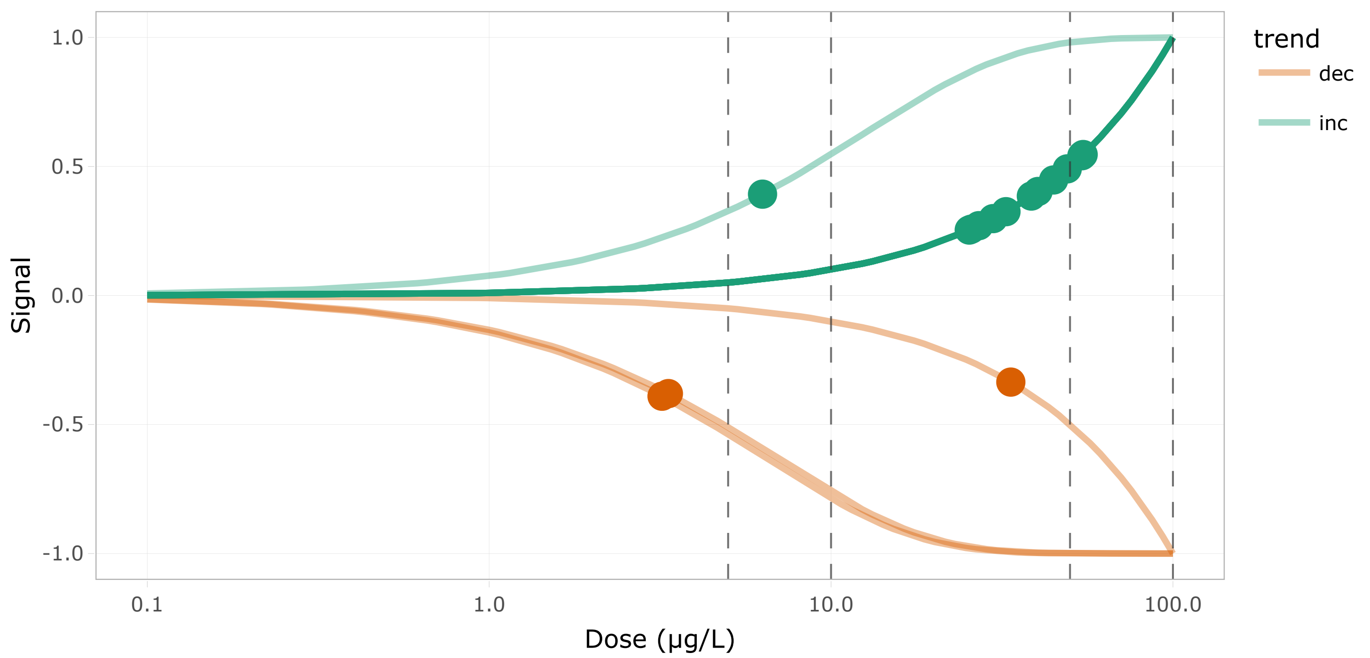

Figure 69: DR curves for the 34th cluster

Show 10 entries Search:

	id	BMD.zSD	TF	trend	friendliness
	All	All	All	All	All
1	adka_t2	3.344	false	dec	1
2	adkb_t2	54.629	false	inc	1
3	ak1	6.303	false	inc	1
4	cad_t2	27.057	false	inc	3
5	dut	32.481	false	inc	1
6	gldc	25.707	false	inc	1
7	impdh1b_t2	33.561	false	dec	1
8	mthfd1a_t2	3.204	false	dec	1
9	nme6	38.532	false	inc	1
10	nt5c2a	49.076	false	inc	1

Showing 1 to 10 of 15 entries

Previous 1 2 Next

Table 44: Table of the 34th cluster content

Cluster 19 - 26 transcripts (BMD q25 = 25.56 µg/L)

Driver GO terms : transmembrane receptor protein serine/threonine kinase signaling pathway

KEGG pathways : Cytokine-cytokine receptor interaction, TGF-beta signaling pathway

Wikipathways :

Curvesplot BMDplot

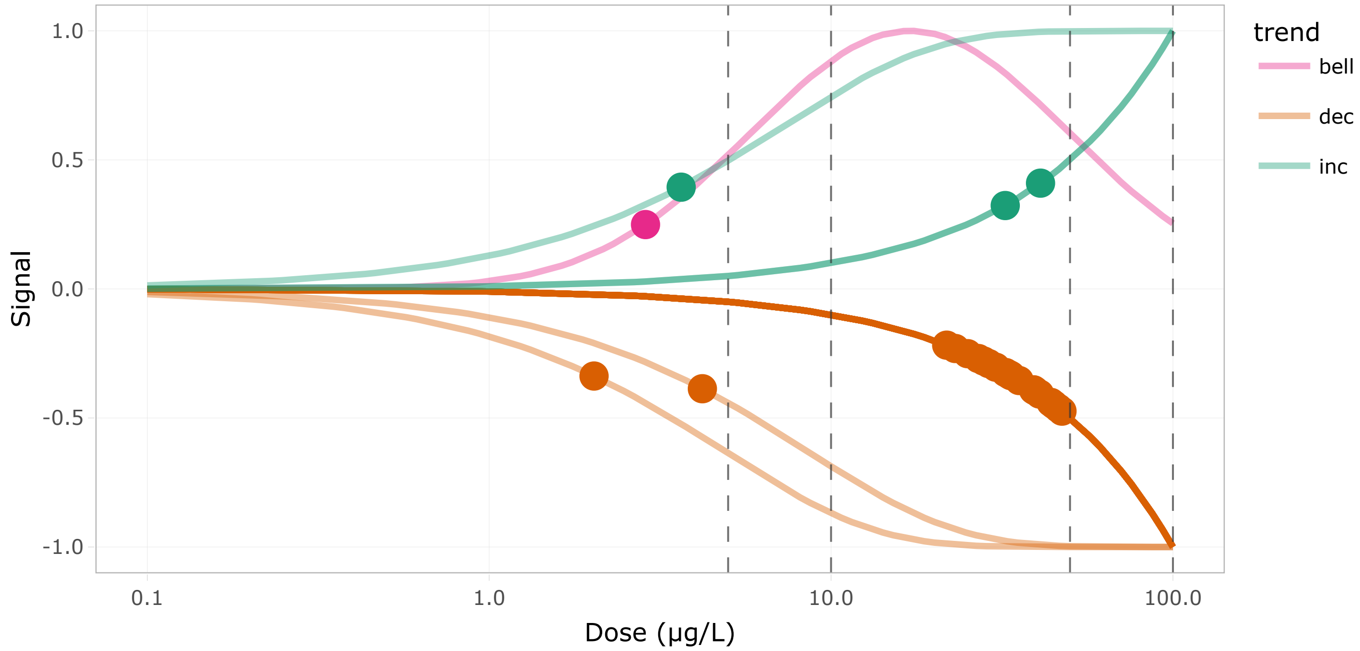

Figure 71: DR curves for the 35th cluster

Show 10 entries Search:

	id	BMD.zSD	TF	trend	friendliness
	All	All	All	All	All
1	acvr1ba	39.099	false	dec	1
2	acvr1bb_t2	26.966	false	dec	1
3	acvr2ba_t3	25.085	false	dec	1
4	acvr2bb	39.523	false	dec	1
5	bmpr2a	46.195	false	dec	1
6	bmpr2b	39.152	false	dec	1
7	flcn_t2	3.644	false	inc	1
8	hdr_t2	2.026	false	dec	3
9	hjv_t2	32.319	false	inc	1
10	il10rb_t2	40.99	false	inc	1

Showing 1 to 10 of 26 entries

Previous 1 2 3 Next

Table 45: Table of the 35th cluster content

Cluster 18 - 6 transcripts (BMD q25 = 25.82 µg/L)

Driver GO terms :

KEGG pathways :

Wikipathways :

Curvesplot | BMDplot

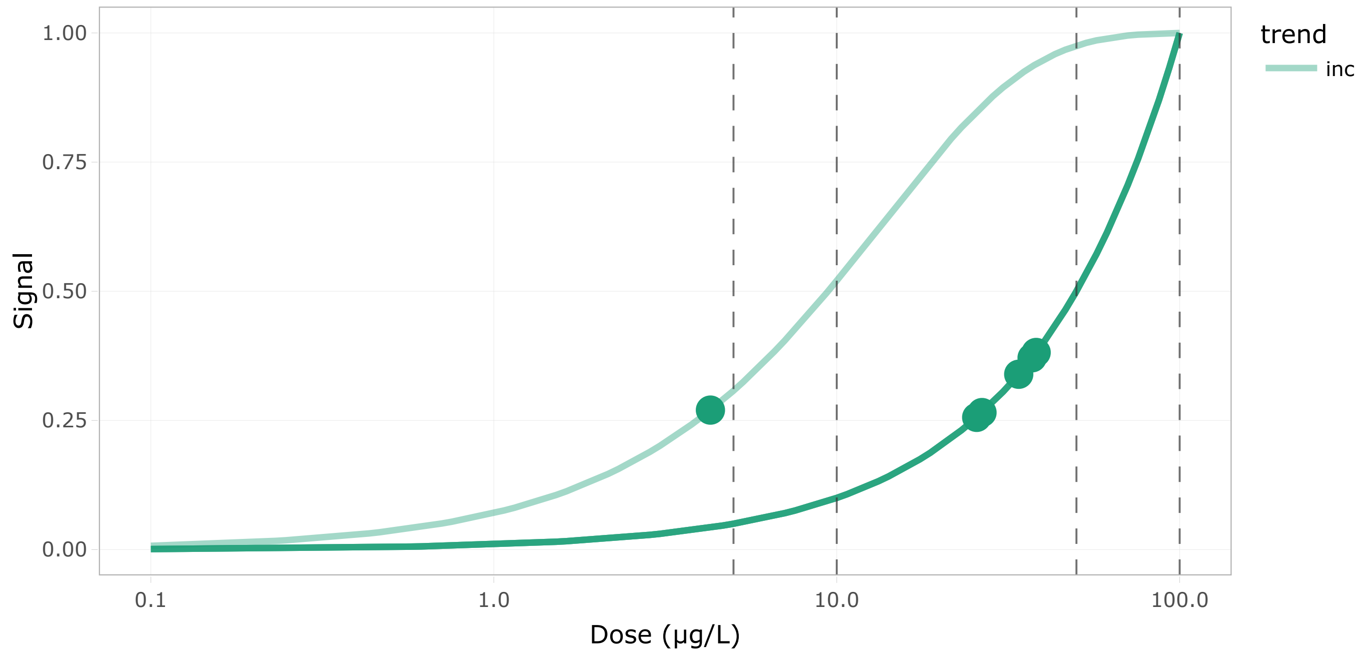

Figure 73: DR curves for the 36th cluster

Show 10 entries | Search:

	id	BMD.zSD	TF	trend	friendliness
	All	All	All	All	All
1	HTRA2_g1t2	4.282	false	inc	1
2	birc5a	25.592	true	inc	1
3	cdca8	33.936	false	inc	1
4	melk	26.522	false	inc	1
5	nusap1	37.128	false	inc	1
6	ube2c_g2t1	38.154	false	inc	1

Showing 1 to 6 of 6 entries | Previous 1 Next

Table 46: Table of the 36th cluster content

Cluster 36 - 10 transcripts (BMD q25 = 26.62 µg/L)

Driver GO terms : regulatory ncRNA-mediated gene silencing

KEGG pathways :

Wikipathways :

Curvesplot | BMDplot

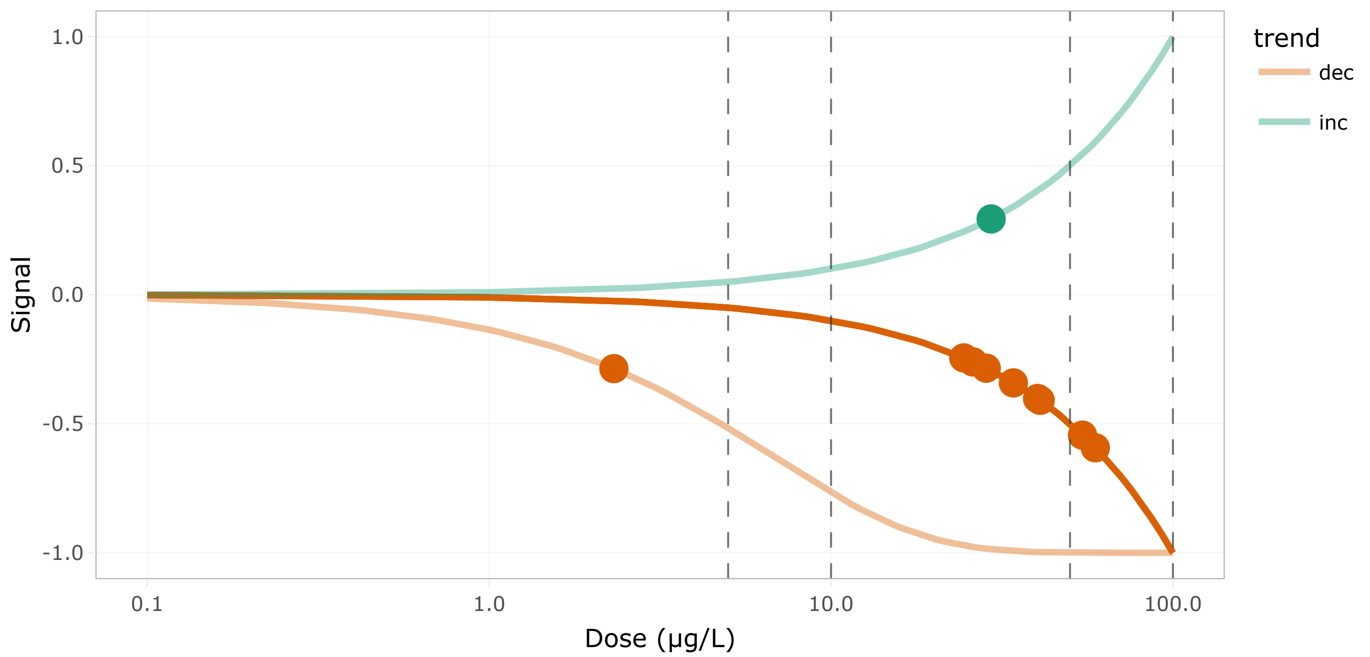

Figure 75: DR curves for the 37th cluster

Show 10 entries | Search:

	id	BMD.zSD	TF	trend	friendliness
	All	All	All	All	All
1	ago1	40.176	true	dec	1
2	ago2	28.433	true	dec	1
3	ago4	54.387	false	dec	1
4	dicer1_g1t1	26.013	false	dec	1
5	eri1_g2t1	29.392	false	inc	3
6	pum1_t2	34.146	false	dec	1
7	si:dkey-46g23.1_t1	40.9	false	dec	2
8	snd1_t2	2.317	true	dec	1
9	tarbp2	24.448	false	dec	1
10	tnrc6c1_t4	59.285	false	dec	2

Showing 1 to 10 of 10 entries

Previous 1 Next

Table 47: Table of the 37th cluster content

Cluster 9 - 10 transcripts (BMD q25 = 27.62 μg/L)

Driver GO terms : transcription by RNA polymerase I, transcription by RNA polymerase III

KEGG pathways : Cytosolic DNA-sensing pathway, RNA polymerase

Wikipathways :

Curvesplot BMDplot

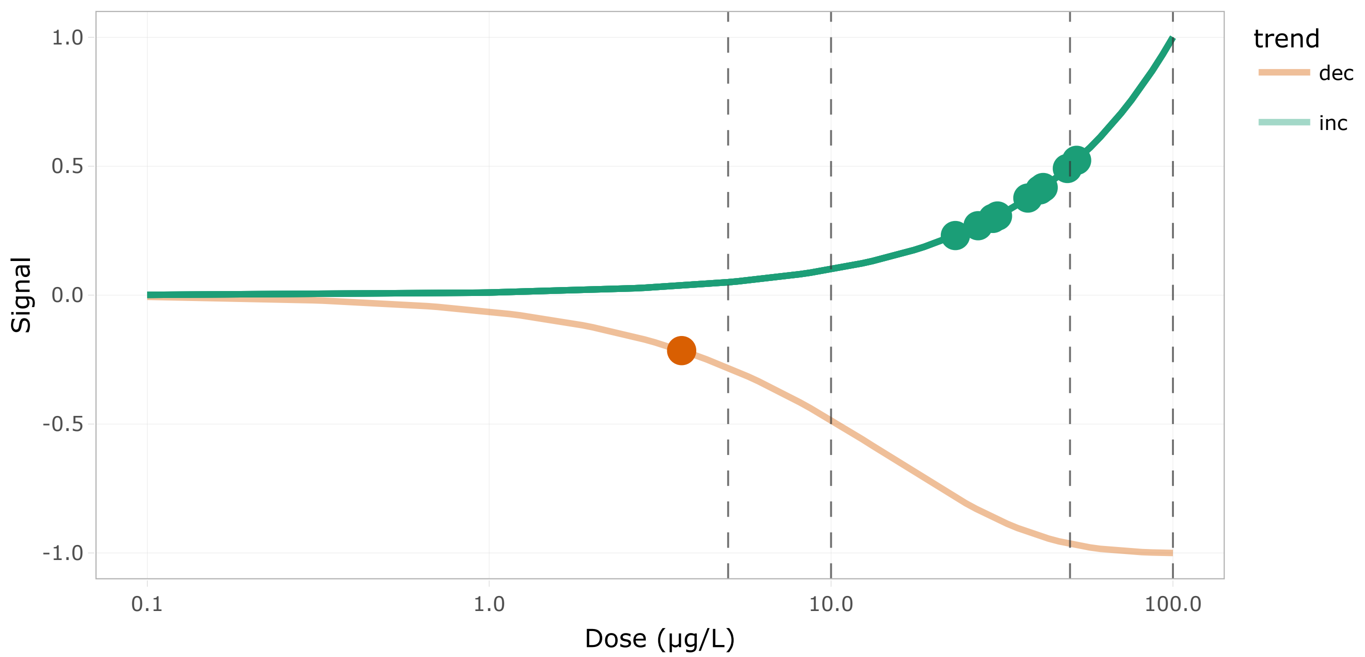

Figure 77: DR curves for the 38th cluster

Show 10 entries Search:

	id	BMD.zSD	TF	trend	friendliness
	All	All	All	All	All
1	irf7_t2	23.102	true	inc	3
2	polr1c_t2	40.827	false	inc	1
3	polr2c_g2t1	30.679	false	inc	1
4	polr2eb	52.268	false	inc	1
5	polr2f_g1t1	3.656	false	dec	1
6	polr2h	29.745	false	inc	1
7	polr2l	41.724	false	inc	1
8	polr3k	26.916	false	inc	1
9	rrn3_t2	37.641	true	inc	1
10	snapc1b	49.114	false	inc	1

Showing 1 to 10 of 10 entries Previous 1 Next

Table 48: Table of the 38th cluster content

Cluster 7 - 23 transcripts (BMD q25 = 27.65 µg/L)

Driver GO terms : nucleotide-excision repair

KEGG pathways : Nucleotide excision repair

Wikipathways : Estrogen signaling

Curvesplot BMDplot

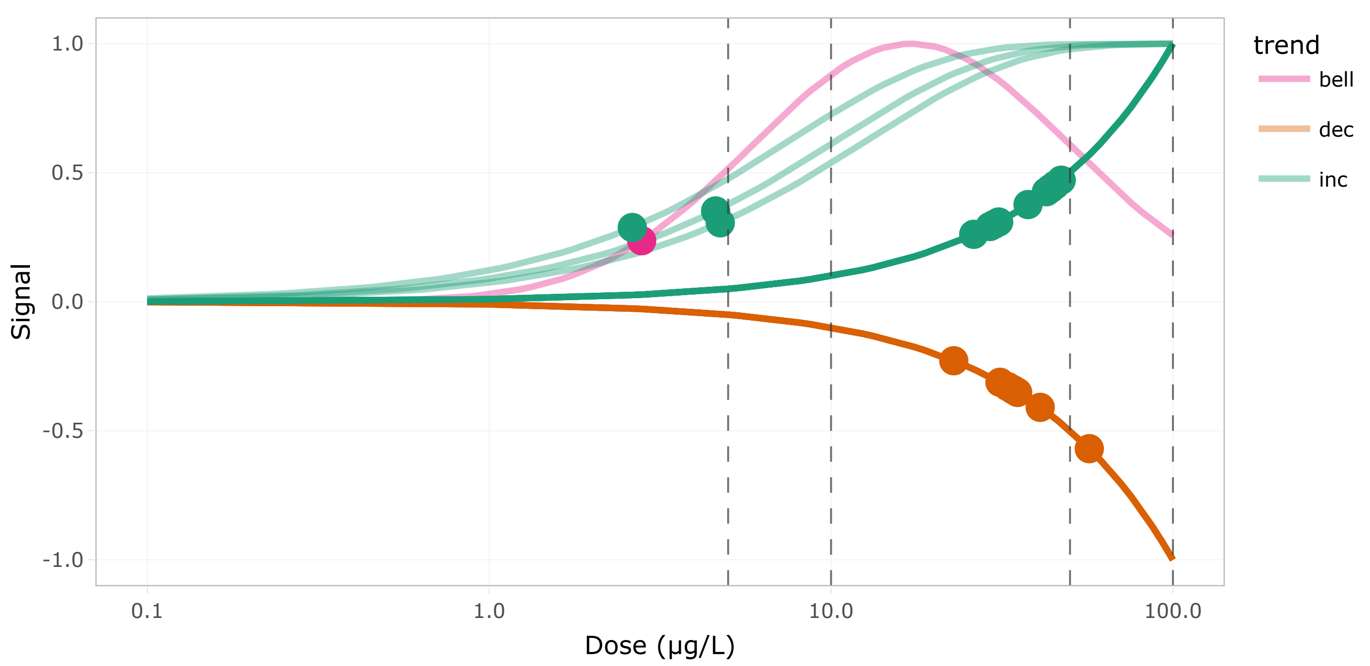

Figure 79: DR curves for the 39th cluster

Show 10 entries Search:

	id	BMD.zSD	TF	trend	friendliness
	All	All	All	All	All
1	braf_t2	2.794	false	bell	4
2	cdk12_t3	22.853	false	dec	1
3	cdk8_g2t1	40.907	true	dec	1
4	ercc3	37.679	true	inc	1
5	ercc8_g1t2	4.596	false	inc	1
6	gnas_t2	34.857	false	dec	2
7	gnb1a	56.928	false	dec	1
8	gtf2e2_g2t3	43.667	false	inc	1
9	gtf2h3	29.163	true	inc	1
10	gtf2h4_t1	33.911	false	dec	2

Showing 1 to 10 of 23 entries Previous 1 2 3 Next

Table 49: Table of the 39th cluster content

Cluster 24 - 5 transcripts (BMD q25 = 28.76 µg/L)

Driver GO terms :

KEGG pathways :

Wikipathways :

Curvesplot BMDplot

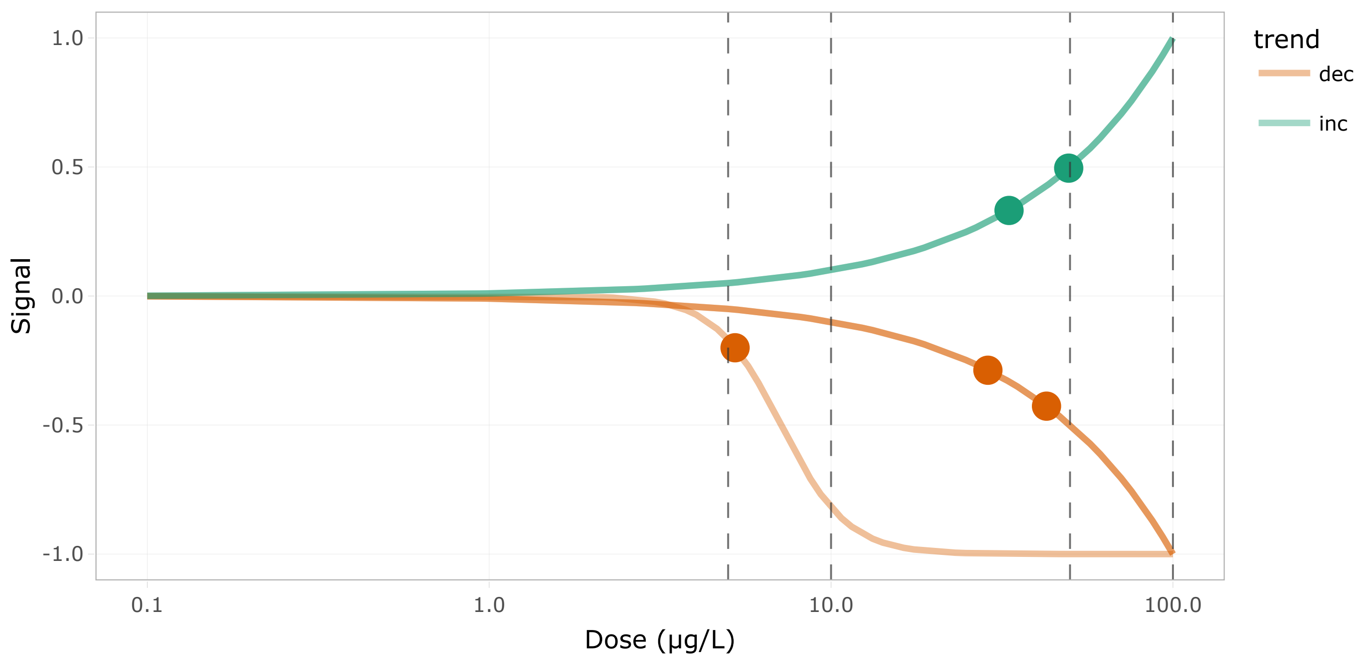

Figure 81: DR curves for the 40th cluster

Show 10 entries Search:

	id	BMD.zSD	TF	trend	friendliness
	All	All	All	All	All
1	acta1b	28.758	false	dec	1
2	cttn_t2	5.238	false	dec	1
3	pleca_t5	42.677	false	dec	1
4	scinla	33.157	false	inc	1
5	tpma_t3	49.555	false	inc	1

Showing 1 to 5 of 5 entries Previous 1 Next

Table 50: Table of the 40th cluster content

Cluster 38 - 17 transcripts (BMD q25 = 29.77 µg/L)

Driver GO terms :

KEGG pathways : Nucleocytoplasmic transport

Wikipathways :

Curvesplot | BMDplot

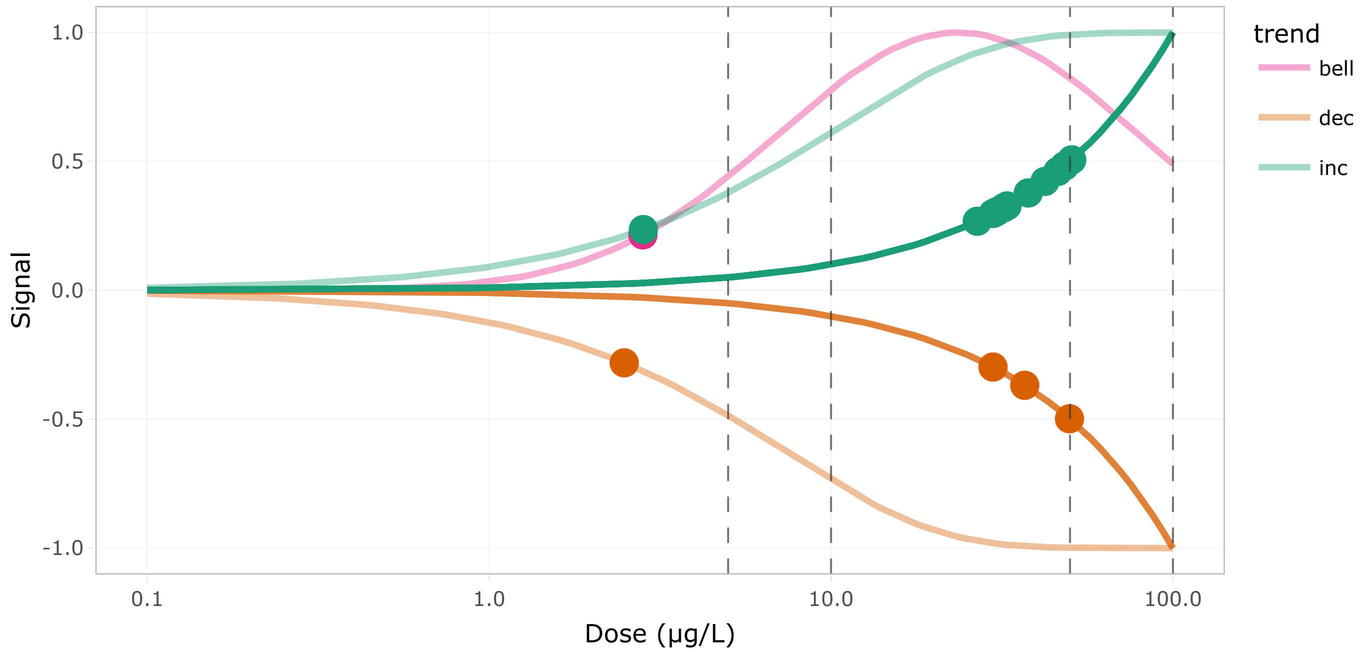

Figure 83: DR curves for the 41th cluster

Show 10 entries

Search:

	id	BMD.zSD	TF	trend	friendliness
	All	All	All	All	All
1	ahctf1_t2	48.302	true	inc	1
2	eef1a1l1_t2	29.782	false	inc	2
3	eef1a1l1_t3	49.818	false	dec	2
4	kpna1	2.816	false	bell	2
5	kpna3	2.483	false	dec	2
6	kpna6_t2	36.855	false	dec	1
7	nmd3	48.099	false	inc	3
8	nup205_g1t3	42.231	false	inc	1
9	nup37_t2	2.825	false	inc	1
10	nup43_t3	37.734	false	inc	1

Showing 1 to 10 of 17 entries

Previous 1 2 Next

Table 51: Table of the 41th cluster content

Cluster 35 - 15 transcripts (BMD q25 = 31.17 µg/L)

Driver GO terms : ribosome biogenesis

KEGG pathways : Ribosome biogenesis in eukaryotes

Wikipathways :

Curvesplot | BMDplot

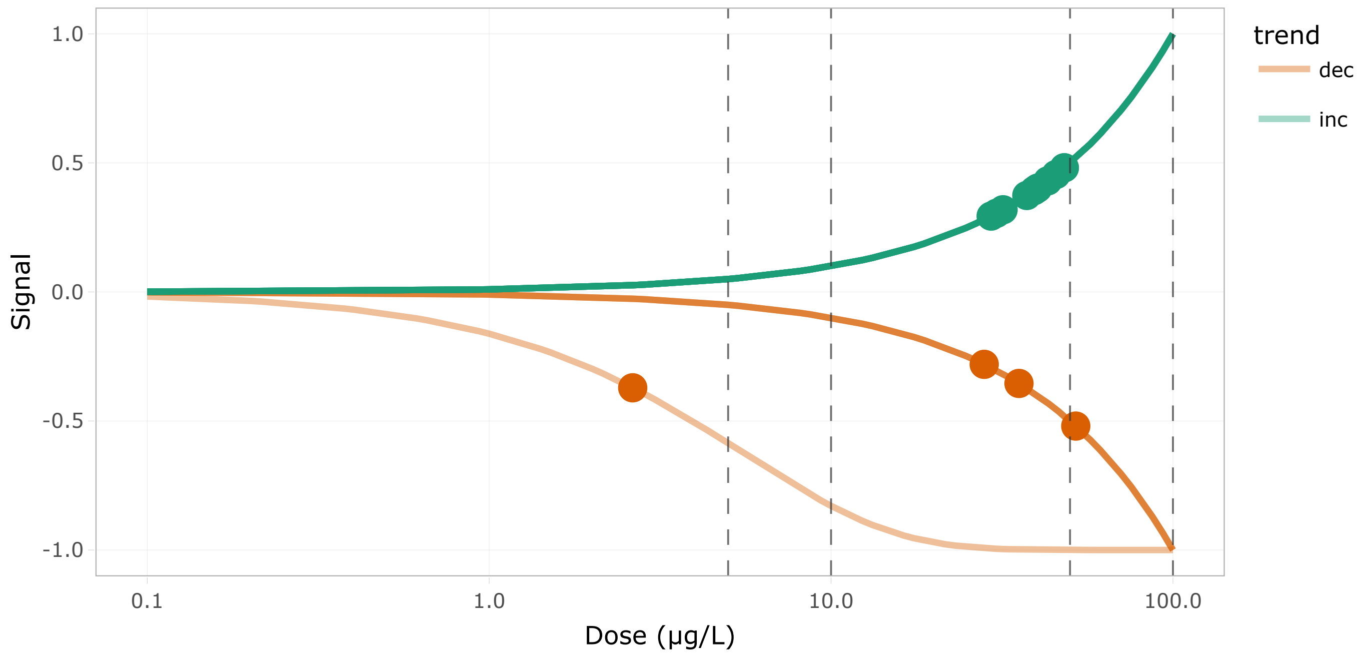

Figure 85: DR curves for the 42th cluster

Show 10 entries

Search:

	id	BMD.zSD	TF	trend	friendliness
	All	All	All	All	All
1	UTP14C	51.973	false	dec	3
2	emg1	48.122	false	inc	1
3	eri1_g2t1	29.392	false	inc	3
4	nip7	45.505	false	inc	2
5	nmd3	48.099	false	inc	3
6	noc4l	43.046	false	inc	1
7	nop58	2.629	false	dec	1
8	pin4	37.377	false	inc	2
9	ran	30.488	true	inc	3
10	rrp7a	39.912	false	inc	1

Showing 1 to 10 of 15 entries

Previous 1 2 Next

Table 52: Table of the 42th cluster content

Cluster 27 - 5 transcripts (BMD q25 = 31.66 µg/L)

Driver GO terms : mismatch repair

KEGG pathways :

Wikipathways :

Curvesplot BMDplot

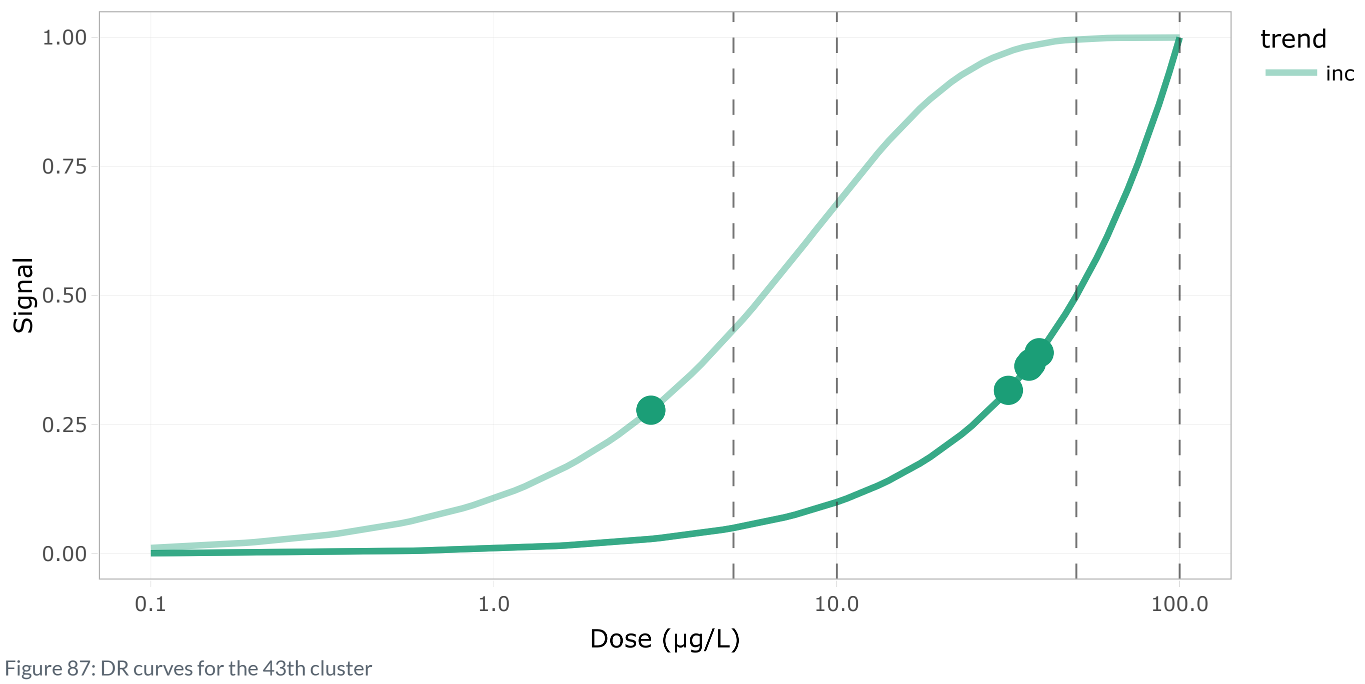

Figure 87: DR curves for the 43th cluster

Show 10 entries Search:

	id	BMD.zSD	TF	trend	friendliness
	All	All	All	All	All
1	mlh1_t2	2.871	false	inc	1
2	msh2	36.922	false	inc	1
3	msh6	38.941	false	inc	1
4	pms1_t2	36.337	true	inc	1
5	recql4	31.657	false	inc	1

Showing 1 to 5 of 5 entries Previous 1 Next

Table 53: Table of the 43th cluster content

Cluster 51 - 4 transcripts (BMD q25 = 33.6 µg/L)

Driver GO terms :

KEGG pathways :

Wikipathways :

Curvesplot BMDplot

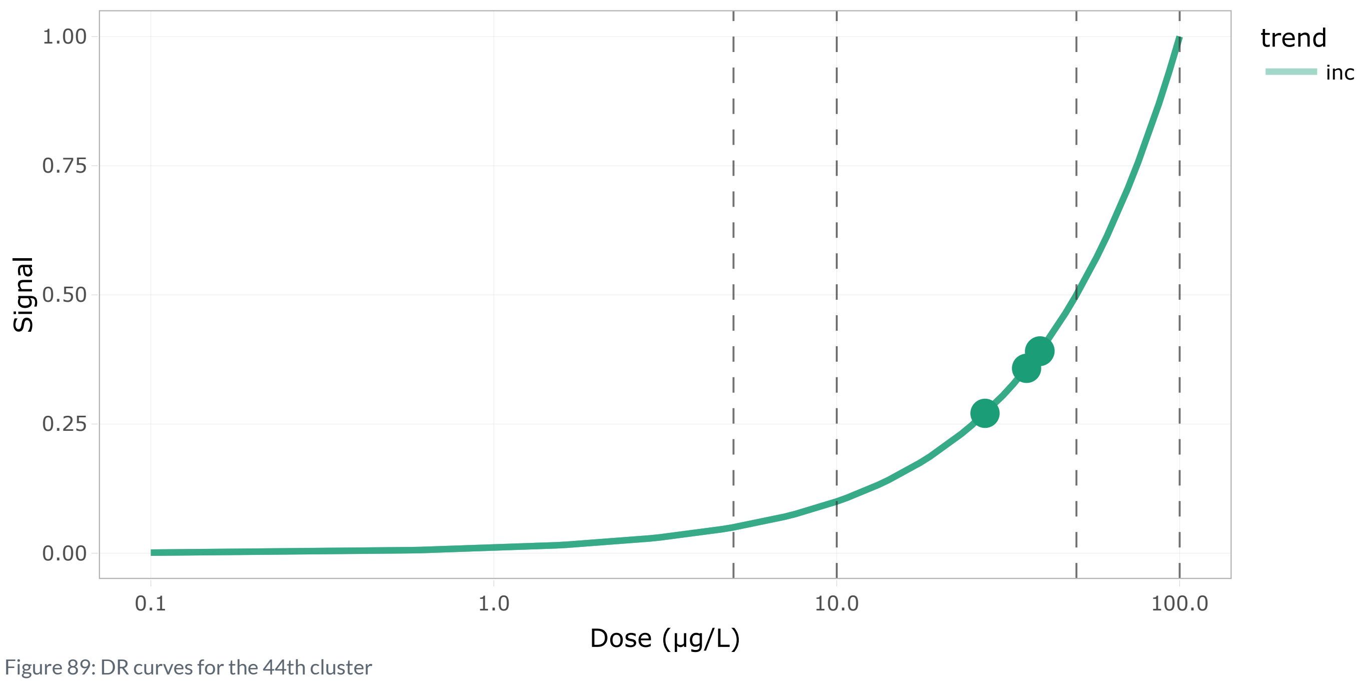

Figure 89: DR curves for the 44th cluster

Show 10 entries Search:

	id	BMD.zSD	TF	trend	friendliness
	All	All	All	All	All
1	tomm22	39.032	false	inc	1
2	tomm5	27.065	false	inc	1
3	tomm6_t1	35.774	false	inc	1
4	tomm7_t2	39.152	false	inc	1

Showing 1 to 4 of 4 entries

Previous 1 Next

Table 54: Table of the 44th cluster content
