# Supplementary material for: Cluefish: mining the dark matter of transcriptional data series with over-representation analysis enhanced by aggregated biological prior knowledge
Source: NAR Genom Bioinform. 2025 Jul 30;7(3):lqaf103. doi: 10.1093/nargab/lqaf103 (PMC12309373; doi:10.1093/nargab/lqaf103)
Supplement: lqaf103_Supplemental_Files [file lqaf103_supplemental_files.zip › supp-4.pdf]

Report of the lonely cluster results

AUTHOR
Ellis Franklin

PUBLISHED
2024-08-07

1 Introduction

This document is a comprehensive report, meticulously encapsulating the pivotal results derived from the DRomics analysis while detailing the workflow. It succinctly summarizes the impacts and outputs of each significant step within the pipeline, supported by visual representations like plots depicting the summary of BMD values per cluster, aiding in prioritization. Moreover, additional specific visualizations for each cluster, such as plots of fitted curves, empirical cumulative distribution function (ECDF) plots of BMD values, and summary tables, enable a more focused exploration.

This document serves as a supplementary report to the workflow_results_report, focusing on the Lonely cluster. The Lonely cluster comprises genes that remain unassociated with any other cluster throughout the workflow process.

The lonely cluster is composed of a total of 1493.

Functional enrichment analysis is conducted to link the cluster with biological processes, facilitating deeper exploration of aspects potentially overlooked in the analysis.

2 The Lonely cluster as an interactive table

Table 1: Summary table of the lonely cluster

Show 10 entries Search:

	transcript_id	gene_id	gene_name	NewCluster	Friendliness	Term_name	Source
1	ENSDART00000000069.8	ENSDARG00000000068	slc9a3r1a_g2t1	Lonely	1		
2	ENSDART00000000070.7	ENSDARG00000000069	dap_g2t1	Lonely	1		
3	ENSDART00000001691.8	ENSDARG00000001463	tdh2	Lonely	1	Glycine, serine and threonine metabolism	KEGG
4	ENSDART00000001691.8	ENSDARG00000001463	tdh2	Lonely	1	Metabolic pathways	KEGG
5	ENSDART00000002164.9	ENSDARG00000008433	unc45b_t2	Lonely	1		
6	ENSDART00000002279.8	ENSDARG00000021442	cdh11	Lonely	1		
7	ENSDART00000002398.7	ENSDARG00000018264	trim101	Lonely	1		
8	ENSDART00000002469.7	ENSDARG00000018989	hspa4b_g2t1	Lonely	1		
9	ENSDART00000003133.10	ENSDARG00000021399	yipf2_t2	Lonely	1		
10	ENSDART00000003170.5	ENSDARG00000018145	mid1ip1l	Lonely	1		

Showing 1 to 10 of 1,667 entries Previous 1 2 3 4 5 … 167 Next

3 Characterizing the Lonely cluster by functional enrichment

Source	Query size	Background size
GO:BP	763	15468
KEGG	177	5714
WP	35	1383

Table 2: Number of genes involved in the query and background of the ORA

Show 10 entries

Search:

	term_name	term_id	source	term_size	intersection_size	query_size	effective_domain_
1	lens development in camera-type eye	GO:0002088	GO:BP	70	26	763	
2	visual perception	GO:0007601	GO:BP	139	26	763	
3	transition metal ion transport	GO:0000041	GO:BP	41	9	763	
4	regulation of small GTPase mediated signal transduction	GO:0051056	GO:BP	106	15	763	
5	positive regulation of leukocyte migration	GO:0002687	GO:BP	15	5	763	
6	regulation of synapse organization	GO:0050807	GO:BP	23	6	763	
7	Endocytosis	KEGG:04144	KEGG	268	22	177	
8	Lysosome	KEGG:04142	KEGG	143	14	177	
9	Polycomb repressive complex	KEGG:03083	KEGG	72	9	177	
10	Ferroptosis	KEGG:04216	KEGG	46	6	177	

Showing 1 to 10 of 12 entries

Previous 1 2 Next

4 The results as an interactive curvesplot

Cluster Lonely - 1493 transcripts

Curvesplot colored by trend | BMDplot colored by trend

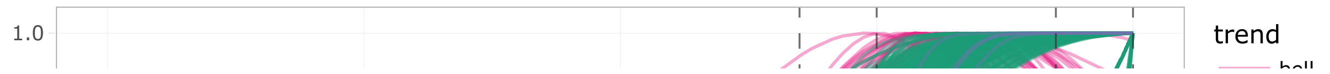

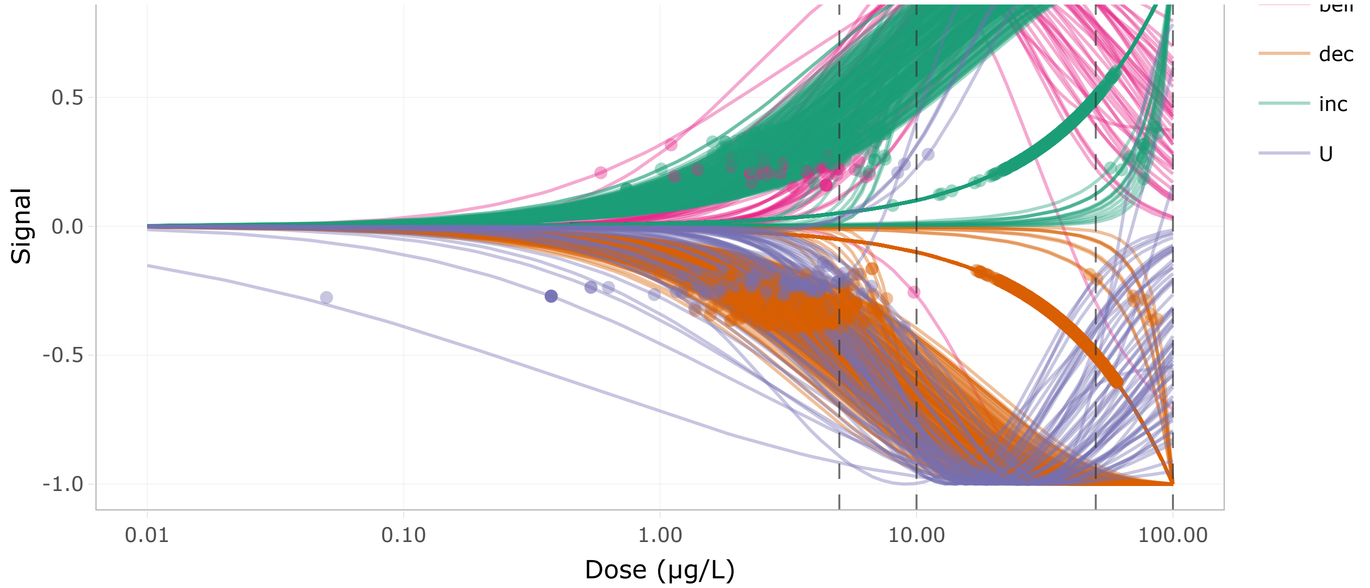

Figure 1: DR curves for the Lonely cluster colored by trend

Curvesplot colored by enriched term

BMDplot colored by enriched term

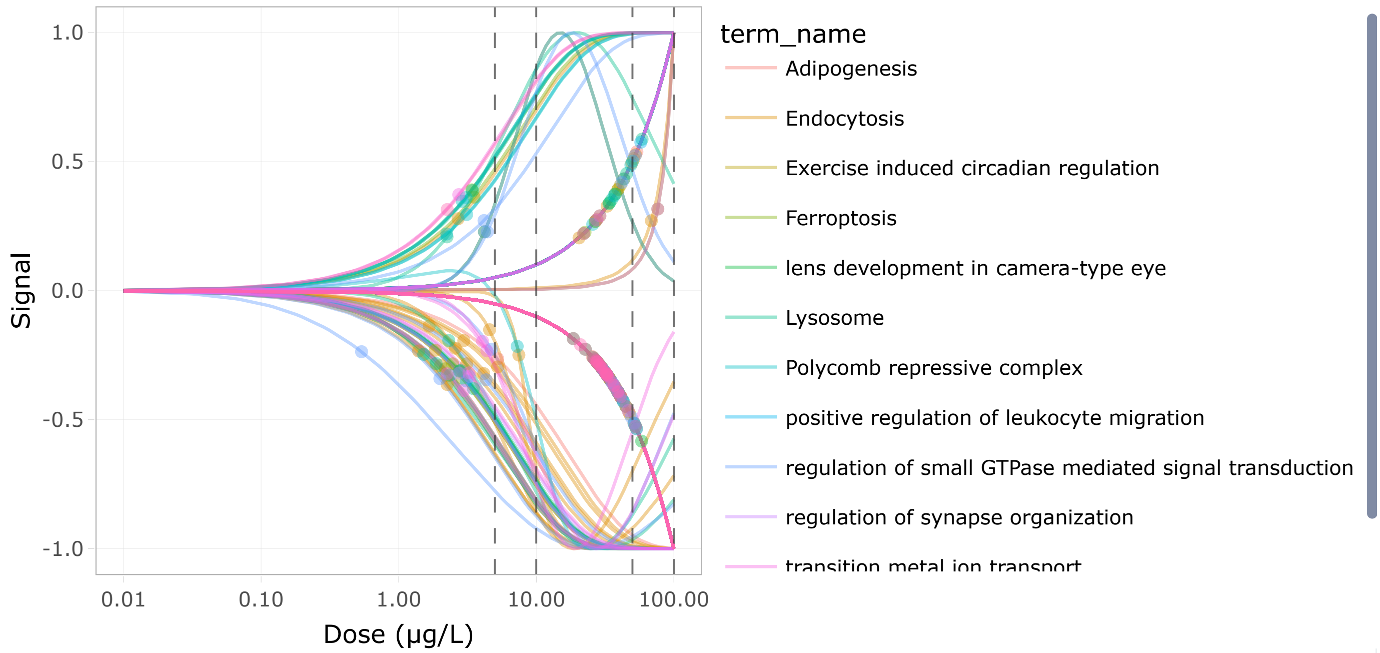

Figure 3: DR curves for all genes associated to the terms enriched by the lonely cluster
