# Supplementary material for: Cluefish: mining the dark matter of transcriptional data series with over-representation analysis enhanced by aggregated biological prior knowledge
Source: NAR Genom Bioinform. 2025 Jul 30;7(3):lqaf103. doi: 10.1093/nargab/lqaf103 (PMC12309373; doi:10.1093/nargab/lqaf103)
Supplement: lqaf103_Supplemental_Files [file lqaf103_supplemental_files.zip › supp-5.pdf]

Report comparison of standard and cluefish workflow

AUTHOR
Ellis Franklin

PUBLISHED
2024-08-07

1 Introduction

The cluefish workflow seeks to enhance the biological interpretation of transcriptomic dose-response modeling data, particularly following the DRomics analysis. Its primary goal is to comprehensively characterize the data while ensuring an unbiased and non-targeted approach. By doing so, we aim to not only maximize the depth of the analysis but also maintain objectivity throughout.

To evaluate the efficacy of this workflow, we can contrast its outcomes with those of a standard workflow, which typically involves functional enrichment analysis on the single list of deregulated transcript genes. This comparative analysis will provide insights into the strengths and potential advantages of cluefish.

2 Comparing initial enriched term content

Here we exclusively compare the enriched terms identified by the two distinct workflows: the standard workflow, which uses the entire gene list as query for the functional enrichment, and the cluefish workflow, which uses all clusters as individual queries. This comparison is conducted separately for each data source.

> **Important**
> This comparison solely focuses on the enriched terms and without any filtering performed. Thus, the results for the cluefish workflow are derived specifically from the **clustrenrich$gostres** output of the **clustrenrich()** function.

Standard workflow results

Show 5 entries   Search:

	term_name	term_id	term_size	query_size	intersection_size	p_value	e
1	peptide biosynthetic process	GO:0043043	355	1509	113	5.555665588728927e-28	
2	translation	GO:0006412	351	1509	111	1.870374801556167e-27	
3	amide biosynthetic process	GO:0043604	408	1509	121	2.2841852128196e-27	

	term_name	term_id	term_size	query_size	intersection_size	p_value	e[illegible]
4	peptide metabolic process	GO:0006518	408	1509	119	2.916188044323696e-26	
5	amide metabolic process	GO:0043603	529	1509	133	9.255574026569427e-23	

Showing 1 to 5 of 95 entries Previous 1 2 3 4 5 … 19 Next

Table 1: Standard workflow functional enrichment results

cluefish workflow results

Show 5 entries Search:

	query	term_name	term_id	term_size	query_size	intersection_size	p_[illegible]
1	1	translation	GO:0006412	351	31	22	8.5805442[illegible]
2	1	ribonucleoprotein complex biogenesis	GO:0022613	175	31	8	2.18651280[illegible]
3	1	SRP-dependent cotranslational protein targeting to membrane	GO:0006614	14	31	2	0.002636779
4	1	embryo development ending in birth or egg hatching	GO:0009792	180	31	4	0.00309650
5	1	myeloid cell differentiation	GO:0030099	89	31	3	0.004890742

Showing 1 to 5 of 289 entries Previous 1 2 3 4 5 … 58 Next

Table 2: cluefish workflow functional enrichment results

Venn diagrams between both workflow per source

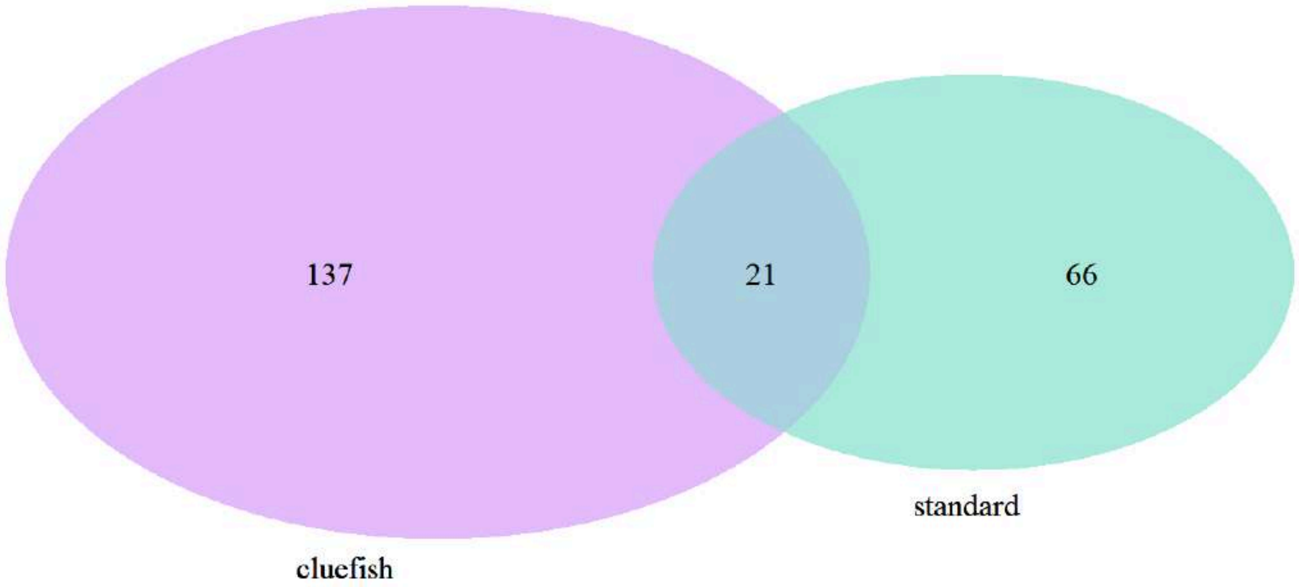

Figure 1: Venn diagram of highlighted enriched GO terms between the standard and cluefish workflow

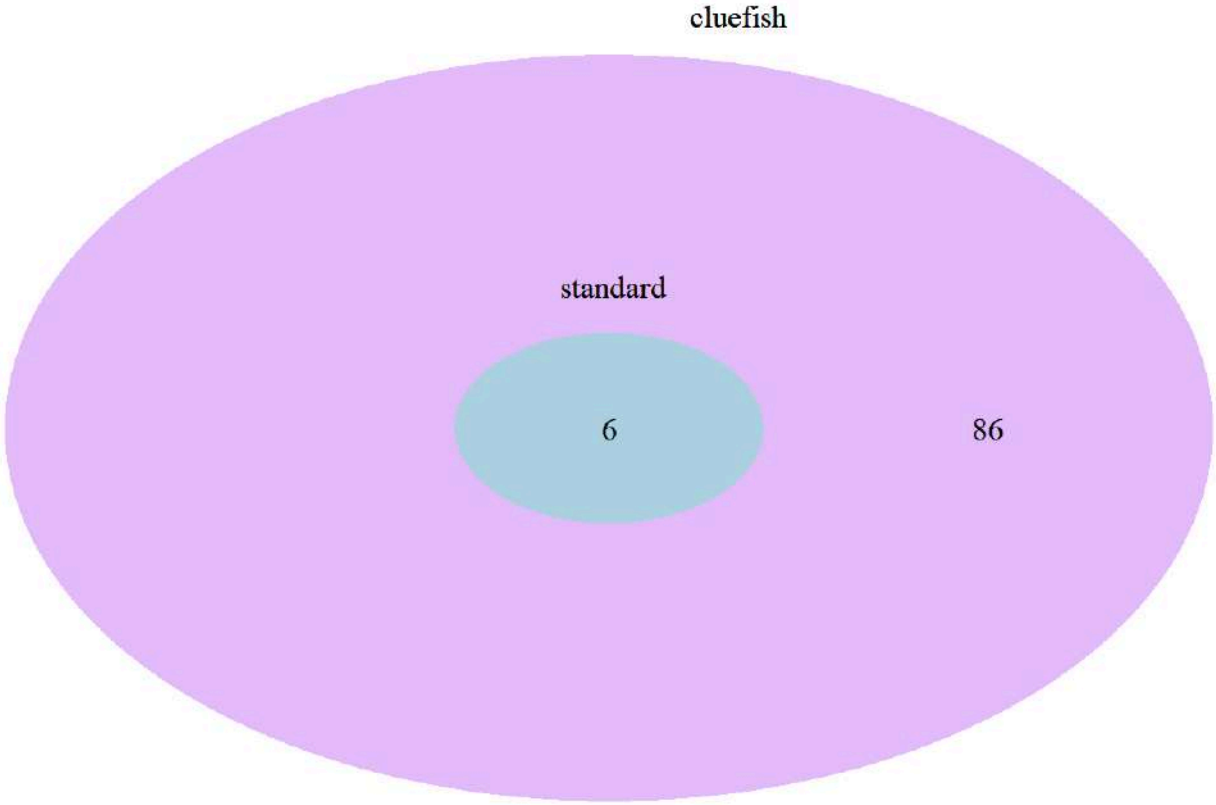

Figure 2: Venn diagram of enriched KEGG pathways between the standard and cluefish workflow

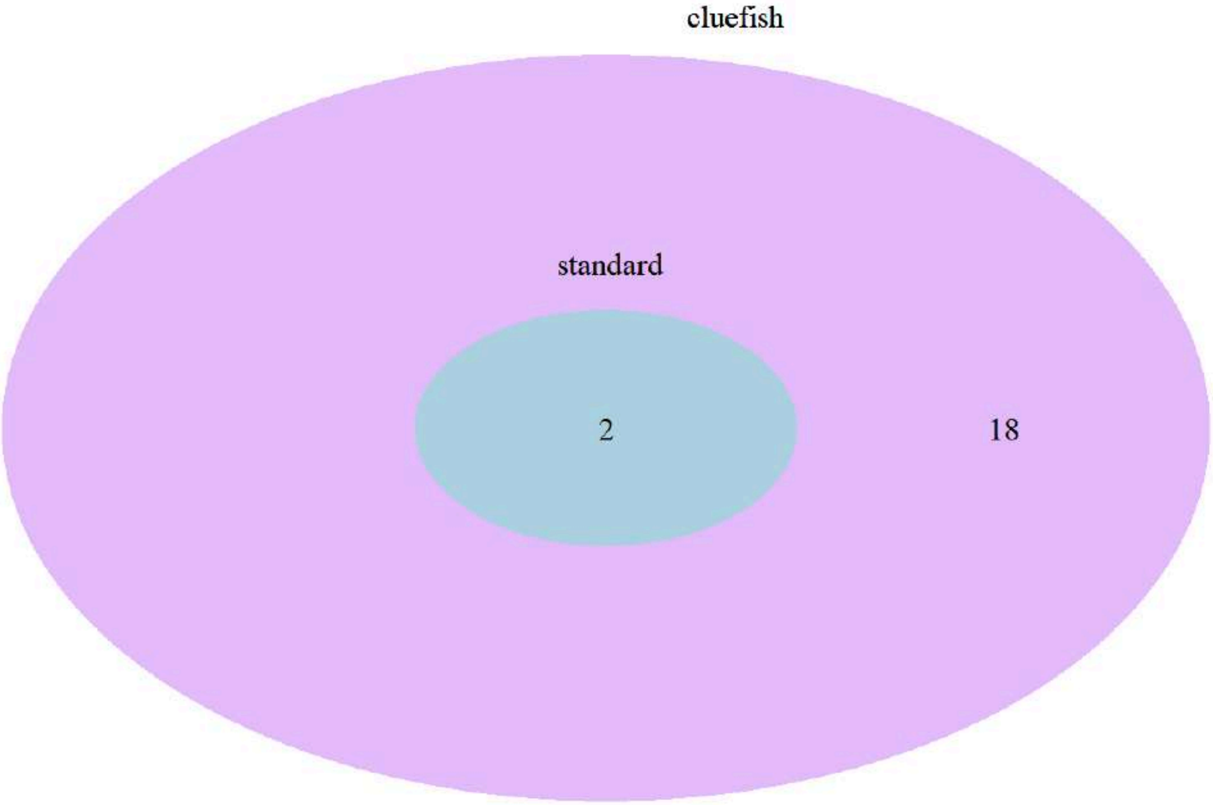

Figure 3: Venn diagram of enriched Wikipathways between the standard and cluefish workflow

3 Comparing enriched term content after filters in the cluefish workflow

With filters applied to the enriched terms within following steps of the standard and cluefish workflow, we can re-examine the discrepancies between enriched terms considered between both workflow.

Standard workflow results

Show 5 entries

Search:

	term_name	term_id	term_size	query_size	intersection_size	p_value
1	peptide biosynthetic process	GO:0043043	355	1509	113	5.555665588728927e-28
2	lens development in camera-type eye	GO:0002088	70	1509	28	4.10849912135549e-9
3	protein folding	GO:0006457	126	1509	31	0.0001116792401074228
4	DNA replication	GO:0006260	92	1509	22	0.003734667164216148
5	intracellular protein transmembrane transport	GO:0065002	14	1509	7	0.00928929320209972

Showing 1 to 5 of 20 entries

Previous 1 2 3 4 Next

Table 3: Standard workflow functional enrichment results

cluefish workflow results

Show 5 entries Search:

	query	term_name	term_id	term_size	query_size	intersection_size	p_
1	1	Cytoplasmic ribosomal proteins	WP:WP324	60	19	18	1.42532120
2	1	embryo development ending in birth or egg hatching	GO:0009792	180	31	4	0.00309650
3	1	myeloid cell differentiation	GO:0030099	89	31	3	0.004890742
4	1	ribonucleoprotein complex biogenesis	GO:0022613	175	31	8	2.18651280
5	1	Ribosome	KEGG:03010	102	21	19	6.38552598

Showing 1 to 5 of 133 entries Previous 1 2 3 4 5 … 27 Next

Table 4: cluefish workflow functional enrichment results

Venn diagrams between both workflows per source after filters in the cluefish workflow

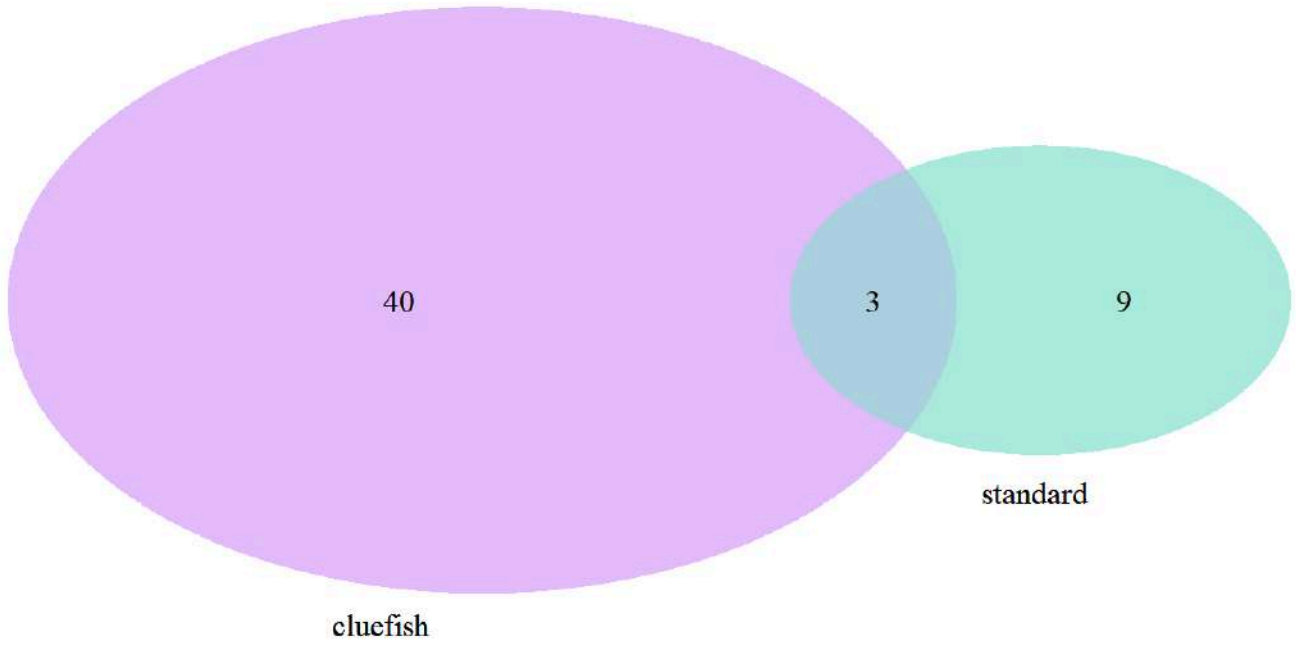

Figure 4: Venn diagram of highlighted enriched GO temrs in the standard and filtered highlighted enriched GO terms in the cluefish workflow

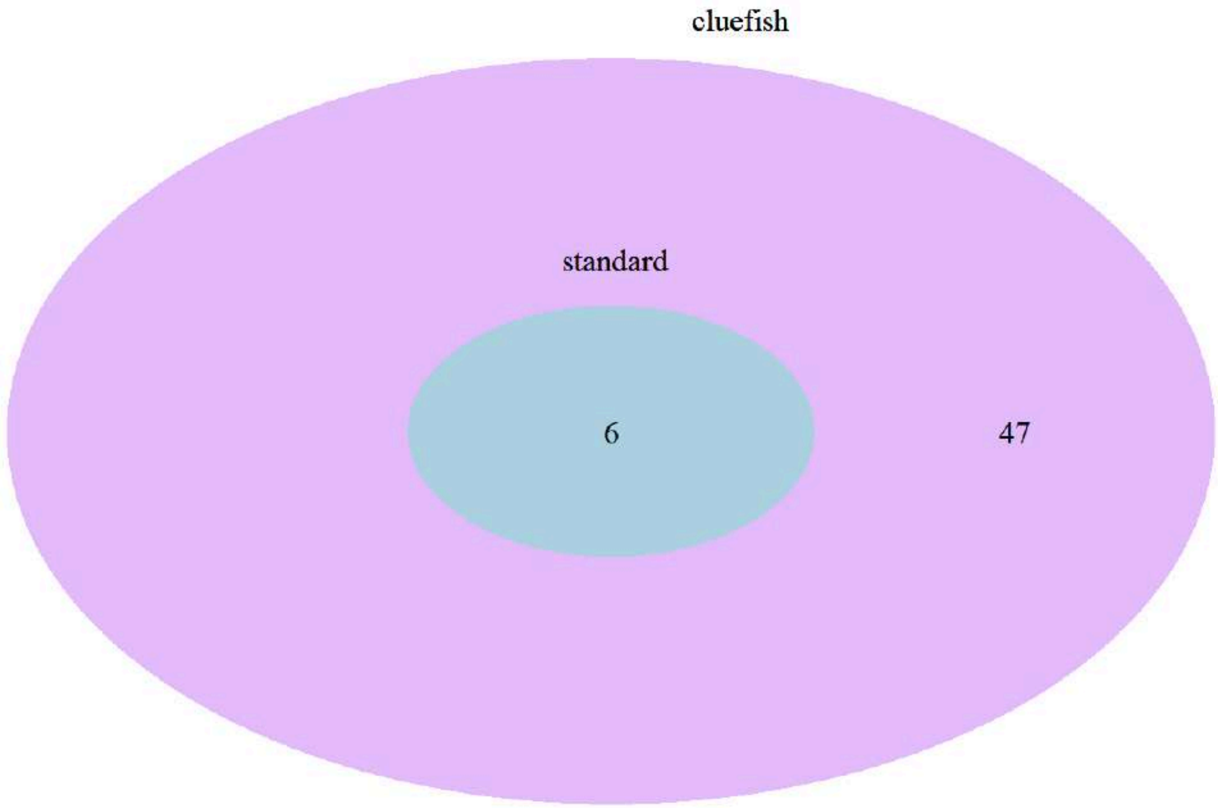

Figure 5: Venn diagram of enriched KEGG pathways in the standard and filtered enriched KEGG pathways in the cluefish workflow

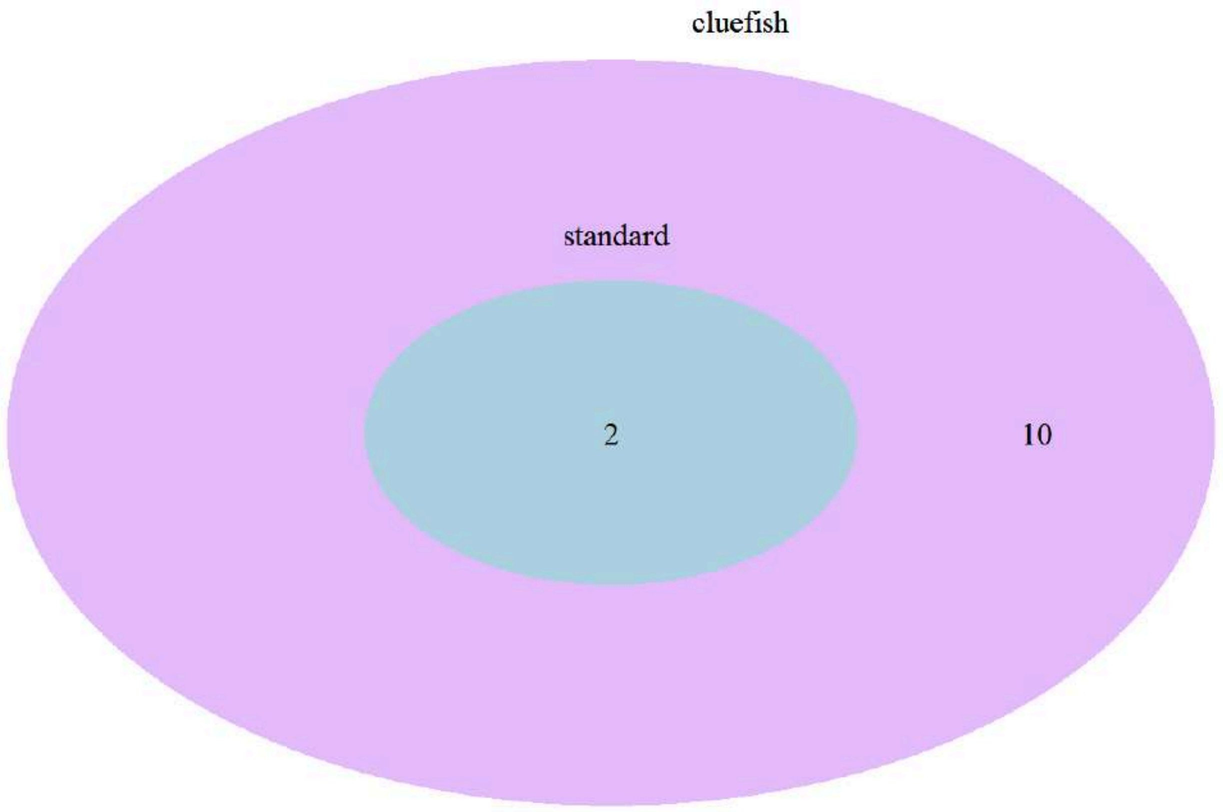

Figure 6: Venn diagram of enriched Wikipathways in the standard and filtered enriched Wikipathways in the cluefish workflow

4 A short summary

The extent of what we can gather, or *what are we working with*, is based on the following question: *how many transcript are considered in the interpretation phase?* And *what is left to be overlooked?*

Metric	Standard	cluefish
Number of transcripts considered	332	940
Number of lonely transcripts	2101	1493

Table 5: Summary of the comparison between both workflow

However, within the cluefish workflow, the lonely cluster consisting of all the lonely content (transcripts/genes) remains open for exploration. Within the **"lonely_results_report.qmd"**, an additional functional enrichment analysis is conducted using the lonely gene list as the query. This approach enables us to delve into what might be overlooked and whether there is any biological significance within this group.
